# Can blood flow restriction amplify the physiological and performance benefits of interval training in male intermittent-sport athletes? a systematic review and meta-analysis

**DOI:** 10.3389/fphys.2026.1864951

**Published:** 2026-07-08

**Authors:** Yixiang Peng, Lei He, Baiyu Liu, Kai Xu, Mingyue Yin, Min Xia

**Affiliations:** 1 Faculty of Health Sciences and Sports, Macao Polytechnic University, Macau, Macao SAR, China; 2 School of Physical Education, Shanghai Normal University, Shanghai, China; 3 School of Athletic Performance, Shanghai University of Sport, Shanghai, China; 4 School of coaching, Shanghai University of Sport, Shanghai, China; 5 School of Physical Education, Shanghai University of Sport, Shanghai, China

**Keywords:** blood flow restricted training, exercise intervention, exercise performance, interval training, physiological adaptations

## Abstract

This systematic review and meta-analysis evaluated the effects of interval training (IT) combined with blood flow restriction (BFR) on physiological adaptations and exercise performance in male intermittent-sport athletes and examined potential moderators. Several databases were searched up to March 2026. Pooled effects were calculated using Hedge’s *g* (*g*) via a three-level random-effects meta-analysis. Moderator analyses were performed based on training status, interval type, training intensity, pressure modes, and restriction mode. A meta-regression analysis was conducted to investigate the potential moderating effect of the training duration on anaerobic capacity. Thirteen studies were included (*n* = 306 males). IT combined with BFR (IT + BFR) was more effective than IT alone for improving anaerobic capacity (*g* = 0.51, 95% CI = 0.08 to 0.93), muscle fitness (*g* = 0.40, 95% CI = 0.23 to 0.57), sprint performance (*g* = 0.40, 95% CI = 0.18 to 0.62) and endurance performance (*g* = 1.17, 95% CI = 0.62 to 1.72) in male intermittent-sport athletes. Subgroup analyses indicated that the moderating effect of training intensity on muscle fitness approached significance (*p* for subgroup = 0.058). Specifically, high-intensity IT (*g* = 0.55, 95% CI = 0.29 to 0.81) appeared to be superior to moderate-intensity IT (*g* = 0.21, 95% CI = -0.04 to 0.46) in enhancing muscle fitness. Muscle fitness improved significantly following small-sided games (g = 0.49, 95% CI = 0.19 to 0.78) and among well-trained male intermittent-sport athletes (g = 0.47, 95% CI = 0.24 to 0.71), with no subgroup differences (all *p* for subgroups > 0.05). The training duration was a significant moderator of anaerobic capacity (*β* = 0.30, 95% CI = 0.04 to 0.55, *p* = 0.021). The main findings suggest that IT + BFR effectively enhances physiological adaptations (anaerobic capacity and muscle fitness) and exercise performance (sprint and endurance) in male intermittent-sport athletes. These potential benefits appeared to be more pronounced with high-intensity IT + BFR for muscle fitness. Furthermore, a possible association was found between longer training duration and greater improvements in anaerobic capacity.

**Systematic review registration:**

https://www.crd.york.ac.uk/PROSPERO/, identifier CRD420261371749.

## 1 Introduction

Intermittent sports (e.g., team, racket, and combat sports) are characterized by repeated bouts of high-intensity activity interspersed with brief recovery periods over prolonged durations (Drinkwater et al., 2008; Holway and Spriet, 2011; Edel et al., 2024). Performance in these sports relies on the combined contribution of the aerobic and anaerobic energy systems (Stølen et al., 2005; Phomsoupha and Laffaye, 2015). Accordingly, well-developed aerobic and anaerobic capacities are essential for intermittent-sport athletes (Stone and Kilding, 2009; Phomsoupha and Laffaye, 2015; Ramirez-Campillo et al., 2022). Given these demands, practitioners are continually seeking more effective training strategies that can simultaneously target these physical qualities and sustain high-level performance throughout competition.

Interval training (IT), which involves brief bouts of exercise at varying intensities separated by recovery intervals (Coates et al., 2023), is a widely used conditioning method for improving athletic performance (Buchheit and Laursen, 2013a, 2013b). However, traditional high-volume or high-intensity IT (HIIT) imposes substantial mechanical stress on the musculoskeletal system (Metcalfe et al., 2022), predisposing athletes to soft-tissue injuries and maladaptive responses over time (Shariff et al., 2009; Gabbett, 2016). Therefore, there is a need for training strategies that can maintain a strong physiological stimulus while reducing external load.

One promising approach is blood flow restriction (BFR), which partially restricts arterial inflow and substantially restricts venous outflow in the working musculature (Scott et al., 2015; Patterson et al., 2019). By increasing metabolic stress under lower mechanical tension (Hughes et al., 2017), BFR may enable athletes to achieve a comparable internal training stimulus with less external loading (Scott et al., 2023). In this context, IT combined with BFR (IT + BFR) may represent a viable strategy for improving performance-related adaptations while mitigating the injury risks associated with excessive mechanical demands (Willis et al., 2018).

To date, evidence from a systematic review by Chua et al. (2022) suggests that IT + BFR enhances aerobic capacity, muscle fitness, and anaerobic performance. However, that review did not include a meta-analysis to quantify the magnitude of changes. More recently, two additional reviews also reported favorable physiological and performance adaptations associated with IT + BFR in healthy adults (Yin et al., 2025a; Zheng et al., 2025). Nevertheless, the current literature has several important limitations. First, existing reviews have not specifically synthesized the physiological adaptations and performance outcomes of IT + BFR in male intermittent-sport athletes. Given differences in physiological and metabolic characteristics between healthy adults and athletes (Egan and Zierath, 2013; Hawley et al., 2014), these findings may not generalize to these athletes. Second, previous reviews have not examined how participant characteristics and training protocol parameters may moderate these effects in this population. Moreover, previous studies in healthy adults indicate that factors such as training status, training intensity, interval type, and training duration can significantly influence the effects of IT + BFR, with greater benefits often observed among trained individuals, during high-intensity exercise, in long-interval protocols, and over longer training periods (Yin et al., 2025a; Zheng et al., 2025). In light of several recent studies published since the most recent review (Mckee et al., 2025; Xia et al., 2025; Xu et al., 2025; Liu et al., 2026), an updated systematic review and meta-analysis is warranted to comprehensively evaluate the effects of IT + BFR on physiological adaptations and performance enhancement in male intermittent-sport athletes.

Therefore, the present study aimed to conduct a three-level meta-analysis evaluating the efficacy of IT + BFR on muscle fitness, aerobic capacity, anaerobic capacity, endurance performance, and sprint performance in male intermittent-sport athletes. This advanced modeling approach was selected to account for the statistical dependency among multiple effect sizes derived from the same study or participant sample. Additionally, we aimed to investigate potential duration-response relationships and explore how participant characteristics and training protocol parameters may moderate these outcomes.

## 2 Methods

The systematic review adhered to the 2020 Preferred Reporting Items for Systematic Reviews and Meta-Analyses (PRISMA) statement (Page et al., 2021). Additionally, this review was registered in the International Prospective Register of Systematic Reviews (PROSPERO) (CRD420261371749).

### 2.1 Literature search

Our systematic review searched PubMed, Web of Science, Cochrane Database, Scopus, and CNKI from inception to March 14, 2026. The search strategy was developed based on previously published systematic reviews. The following search terms were used: (“occlusion training” OR “occluded training” OR “blood flow restricted” OR “blood flow restriction” OR “KAATSU”) AND (“aerobic interval” OR “games” OR “interval training” OR “repeated sprint” OR “short interval” OR “long interval” OR “sprint interval”) AND (“basketball” OR “boxing” OR “badminton” OR “football” OR “rugby”) (the detailed search strategy is presented in **Appendix S2**). Additionally, PROSPERO was searched to identify any existing or ongoing protocols for related systematic reviews. Finally, to ensure comprehensive literature coverage, the reference lists of relevant reviews were manually searched.

### 2.2 Selection process

Deduplication of the retrieved records was performed manually by an independent reviewer (P.Y.X.) using EndNote X9 (Clarivate Analytics, Philadelphia, PA, USA). Then, two independent researchers (P.Y.X. and H.L.) screened the titles and abstracts. If consensus could not be reached, a third independent researcher (L.B.Y.) reviewed the article to determine its eligibility. Finally, two researchers (P.Y.X. and H.L.) independently reviewed the full texts of selected articles for final inclusion. Any discrepancies were resolved according to the predefined inclusion and exclusion criteria.

### 2.3 Selection criteria

*A priori* inclusion and exclusion criteria were defined to assess study eligibility according to the PICOS framework: (1) male intermittent-sport athletes as participants; (2) supervised interventions with clearly defined protocol variables and a duration of at least 2 weeks (Gibala et al., 2006); (3) any type of IT group as the comparator; (4) at least one outcome related to aerobic capacity (maximal oxygen uptake and aerobic power, assessed using continuous or incremental laboratory tests or estimated from field tests), anaerobic capacity (peak and mean power output measured using the 30s Wingate anaerobic test), muscle fitness (e.g., countermovement jump, bench press, back squat, muscle thickness, and integrated electromyography), sprint performance (sprint and repeated-sprint time and sprint power output), or endurance performance (time trials or time to exhaustion); and (5) randomized controlled trials published in peer-reviewed journals.

Exclusion criteria: (1) studies on therapeutic or disease-related outcomes; (2) animal models or *in vitro* studies; and (3) systematic reviews or meta-analyses, case studies, non-peer-reviewed manuscripts, and conference proceedings; (4) studies not involving male intermittent-sport athletes.

### 2.4 Assessment of methodological quality

Risk of bias was assessed using the Cochrane Risk of Bias tool in Review Manager 5.4 software (Copenhagen: The Nordic Cochrane Center, The Cochrane Collaboration, 2014) (Cohen, 1988). The tool assessed (1) random sequence generation; (2) allocation concealment; (3) blinding of participants and personnel; (4) blinding of outcome assessment; (5) incomplete outcome data; (6) selective reporting; and (7) other bias. Each domain was rated as “low risk” (“+”), “high risk” (“-”), or “unclear risk” (“?”). Quality assessment was performed independently by two investigators (P.Y.X. and H.L.), and discrepancies in the quality assessment were resolved by discussion or through consultation with a third reviewer (L.B.Y.).

Additionally, the Physiotherapy Evidence Database (PEDro) (de Morton, 2009) scale was used to assess methodological quality. The PEDro scale ranges from 0 to 10, with scores ≥ 6 considered high quality, 4–5 considered moderate quality, and ≤ 3 considered low quality.

### 2.5 Data extraction and study coding

Data extraction was independently performed by two researchers (P.Y.X. and H.L.) using Excel (Version 16.93, Microsoft, Redmond, WA, USA). Extracted data included the first author, publication year, study characteristics, participant demographics, training protocols, and relevant outcomes. Any discrepancies were resolved through discussion. Relevant data were extracted using WebPlotDigitizer 4.7 (Drevon et al., 2017) when they were presented exclusively in graphical form. Reported standard errors (SE) were converted to standard deviations (SD) following the Cochrane Handbook guidelines (Cumpston et al., 2019).

### 2.6 Statistical analyses

#### 2.6.1 Calculation of effect size and variance

In this study, between-group comparisons were performed between the IT + BFR and IT alone groups. The mean difference (MD) and the SD of the change in means ( ${\text{SD}}_{\text{pooled}}$) were calculated according to the recommendations of the Cochrane Handbook for the evaluation of intervention systems (version 6.5, 2024), using the following formula (Higgins et al., 2019). The first step involved calculating the difference in means:


$${\text{M}}_{\text{change}}={\text{M}}_{\text{post}}-{\text{M}}_{\text{pre}}$$

where ${\text{M}}_{\text{pre}}$ is the reported mean pre-intervention and ${\text{M}}_{\text{post}}$ is the reported mean post-intervention.

Then the SD of the change in means was calculated as follows (Hedges and Shymansky, 1989):


$${\text{SD}}_{\text{change}}=\sqrt{\left({\text{SD}}_{\text{pre}}^{2}+{\text{SD}}_{\text{post}}^{2}-(2\times r\times {\text{SD}}_{\text{pre}}\times {\text{SD}}_{\text{post}})\right)}$$

where ${\text{SD}}_{\text{change}}$ is the SD of the difference in means, ${\text{SD}}_{\text{pre}}$ is the SD from pre-intervention, ${\text{SD}}_{\text{post}}$ is the SD from post-intervention, and r is the correlation coefficient. Correlation coefficients for pre- and post-intervention were rarely reported in the included studies and were generally assumed to be r = 0.50, as suggested by the Cochrane Handbook (Cumpston et al., 2019). To assess the robustness of our findings, we conducted sensitivity analyses using alternative r values, specifically r = 0.20 (lower bound) and r = 0.80 (upper bound), for the overall performance outcomes.

Then the ${\text{SD}}_{\text{pooled}}$ was calculated as follows (Hedges and Olkin, 1985):


$${\text{SD}}_{\text{pooled}}=\sqrt{\frac{({\text{n}}_{\text{E}}-1){\text{SD}}_{\text{change, E}}^{2}+({\text{n}}_{\text{C}}-1){\text{SD}}_{\text{change, C}}^{2}}{{\text{n}}_{\text{E}}+{\text{n}}_{\text{C}}-2}}$$

where ${\text{n}}_{\text{E}}$ and ${\text{n}}_{\text{C}}$ are the sample sizes of IT + BFR and IT groups, and ${\text{SD}}_{\text{change, E}}$ and ${\text{SD}}_{\text{change, C}}$ are the SDs of the change scores for the respective groups.

Considering the relatively small sample sizes of most included studies, Hedge’s *g* (*g*) was used as the mean effect size point estimate in each analysis, using the following formula (Hedges, 1983):


$$\text{Hedge"s g}=\frac{{\text{M}}_{\text{change, E}}-{\text{M}}_{\text{change, C}}}{{\text{SD}}_{\text{pooled}}}\times (1-\frac{3}{4({\text{n}}_{\text{E}}+{\text{n}}_{\text{C}}-2)-1})$$

*g* was classified as *trivial* (SMD < 0.20), *small* (0.20 ≤ SMD < 0.50), *moderate* (0.50 ≤ SMD < 0.80), or *large* (SMD ≥ 0.80) (Cohen, 1988).

SE of *g* was calculated using the following formula (Hedges, 1983):


$$\text{SE}=\sqrt{\frac{1}{{\text{n}}_{\text{E}}}+\frac{1}{{\text{n}}_{\text{C}}}+\frac{{\text{g}}^{2}}{2({\text{n}}_{\text{E}}+{\text{n}}_{\text{C}})}}$$

#### 2.6.2 Meta-analysis and heterogeneity

A three-level meta-analysis was conducted following the recommendations of Assink and Wibbelink (Assink and Wibbelink, 2016) to address the double counting or missed correlations in exercise performance with nested or multiple effect sizes (e.g., the mean power output, peak power output and total work) (Kadlec et al., 2023). By preserving valuable information from multiple effects within each study, the three-level meta-analysis enhances statistical power and provides a more accurate estimate of effect sizes (Assink and Wibbelink, 2016). This approach decomposes variance into sampling variance (Level 1), within-study variance (Level 2), and between-study variance (Level 3), accounting for correlated and hierarchical effects (Cheung, 2019). For the three-level model, parameters were estimated using the restricted maximum likelihood (REML) method, and results were cross-verified using the maximum likelihood (ML) method to ensure stability. Hedge’s *g* was classified as *trivial* (SMD < 0.20), *small* (0.20 ≤ SMD < 0.50), *moderate* (0.50 ≤ SMD < 0.80), or *large* (SMD ≥ 0.80) (Cohen, 1988).

Tests of individual coefficients and their corresponding 95% confidence intervals (95% CI) were based on a t-distribution. Additionally, we computed the prediction interval (PI) based on the t-distribution, which provides useful additional information compared to the 95% CI, especially considering the use of a random-effects model (Borg et al., 2024). Heterogeneity was assessed using *I²* and PI, with *I²* values classified as 0%–25% (*low*), 25%–50% (*moderate*), 50%–75% (*substantial*), and 75%–100% (*considerable*) (Cumpston et al., 2019). The statistical significance threshold was set at *p* < 0.05.

To explore sources of heterogeneity and potential moderators, subgroup analyses and meta-regression analyses were performed. Meta-regression analyses were conducted for continuous variables (Hopkins and Batterham, 2018). Subgroup analyses were conducted when at least three studies were available (Cumpston et al., 2019), and meta-regression analyses were performed when at least ten studies were available for each model (Ruppar, 2020). The following variables were included in the subgroup analyses: (a) training status; (b) interval type; (c) training intensity; (d) pressure modes; (e) restriction mode.

Based on previous participant categorization frameworks, training status was classified as trained and well-trained (McKay et al., 2022). IT was categorized as follows based on Buchheit and Laursen (Buchheit and Laursen, 2013a): short intervals (duration < 60s per repetition), repeated short sprints (repeated sprints with duration < 10s for each sprint, recovery < 60s), small-sided games (SSG, duration of 2–3 min per repetition), and long intervals (duration > 60s per repetition). Training intensity was categorized as HIIT (repeated bouts of exercise ≥ 80% HR_max_, ≥ 60% VO_2max_, or “all-out effort”) (Gibala, 2018; Poon et al., 2024) and moderate-intensity interval training (MIIT, repeated bouts of exercise < 80% HR_max_ or < 60% VO_2max_). We categorized BFR pressure types into absolute and relative based on a previous study (Patterson et al., 2019). To provide practical insights, exploratory moderator analyses were planned for all primary outcomes regardless of overall heterogeneity, but they were conducted only when sufficient effect sizes were available within subgroups.

To assess the relationship between training duration and IT + BFR, we conducted a mixed-effects meta-regression analysis using REML estimation, which is recognized for its robustness (Zuur et al., 2009). All analyses were conducted using the *metafor* package, and results were visualized using *ggplot2* (Gustavsson et al., 2022). Additionally, the statistical power of each subgroup and primary pooled effect was calculated to prevent the possibility of false negatives due to insufficient statistical power, using the *metameta* package (Quintana, 2023).

#### 2.6.3 Publication bias and sensitivity analysis

Funnel plots (Peters et al., 2008), Egger’s asymmetry test (Egger et al., 1997), and trim-and-fill tests were used to assess publication bias (with analyses conducted only when k > 10 (Sterne et al., 2011)). A *p-*value > 0.05 was considered indicative of no publication bias.

We conducted sensitivity analyses using a leave-one-out method to assess the robustness of all primary pooled effects in three-level meta-analysis and moderator analysis. Furthermore, we conducted Cook’s distance (Viechtbauer and Cheung, 2010) and studentized residuals (Atkinson et al., 1983) to identify outliers and influential cases at both level 2 and level 3. Observations were flagged as potentially influential if their hat values or Cook’s distances exceeded three times their respective means, or if the absolute value of studentized residuals exceeded three. The three-level model was then re-estimated with outliers excluded to examine the robustness of the findings.

### 2.7 Certainty of the evidence

The risk of bias was considered in the interpretation of the results by applying the Grading of Recommendations Assessment, Development, and Evaluation (GRADE) methodology, which rates the certainty of evidence as “*high*” (further research is very unlikely to change our confidence in the estimate of effect), “*moderate*” (further research is likely to have an important impact on our confidence in the estimate of effect and may change the estimate), “*low*” (further research is very likely to have an important impact on our confidence in the estimate of effect and is likely to change the estimate) or “*very low*” (any estimate of effect is very uncertain) (Schünemann et al., 2019). GRADE assessment was evaluated by one reviewer (P.Y.X.) and reviewed by a second reviewer (H.L.).

## 3 Results

### 3.1 Study selection and characteristics

**Figure 1:**
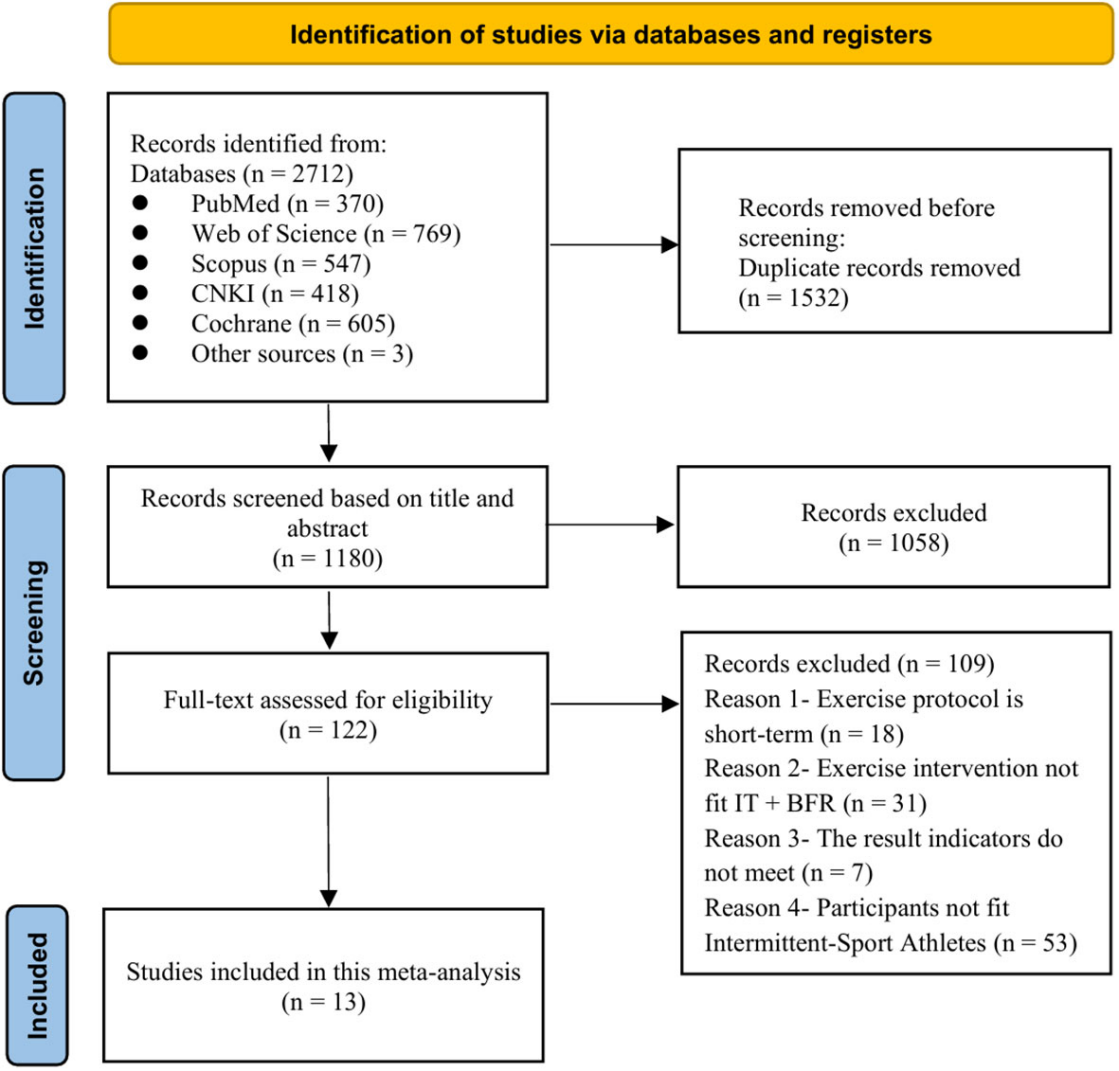
Flow diagram of study selection.

The initial search yielded 2712 publications, including 2709 from the primary database search and 3 obtained from other sources After screening and eligibility assessment, 13 studies (Park et al., 2010; Cook et al., 2014; Amani-Shalamzari et al., 2019, 2020; Elgammal et al., 2020; Zhou et al., 2021; Hosseini Kakhak et al., 2022; Mckee et al., 2024, 2025; Chen et al., 2025; Xia et al., 2025; Xu et al., 2025; Liu et al., 2026) were included in the meta-analysis. A summary of the search process is shown in **Figure 1**.

Across all studies, sample sizes ranged from 12 to 36 participants, for a total of 306 participants. Participants’ ages ranged from 16 to 23 years. Among the participants, 47% were classified as trained (k = 6), and 53% as well-trained (k = 7). The cuffs ranged in width from 4 cm to 13 cm. Pressure modes included fixed pressures (Cook et al., 2014; Zhou et al., 2021; Hosseini Kakhak et al., 2022; Mckee et al., 2024, 2025; Chen et al., 2025; Xia et al., 2025; Xu et al., 2025; Liu et al., 2026) and incremental pressures (Park et al., 2010; Amani-Shalamzari et al., 2020; Elgammal et al., 2020). For more details, please refer to **Table 1**.

**Table 1:** Study characteristics.

			Training protocols				
Study	Design	Participant	BFR combined IT sessions	IT alone sessions	Type	Fre	Week
Park et al., 2010	RCT	n = 12 M Age = 20 ± 1 years, Height = 189 ± 7 cm Body mass = 87 ± 8 kg Trained basketball player Training experience = NA VO_2max_: 48.3 ± 4.3 ml/kg/min	BFR cuff pressure = 160–220 mmHg in leg (absolute) BFR width = 11 cm BFR material: pneumatic cuffs IT protocol: 5 × 3 min at 4–6 km/h (5% grade) with 1 min recovery	IT protocol: 5 × 3 min walk (4–6 km/h on 5% grade) with 1 min recovery	LI	6	2
Cook et al., 2014	RCT	n = 20 M Age = 22 ± 1 years, Height = 184 ± 5 cm Body mass = 96 ± 10 kg well-trained basketball player Training experience ≥ 2 years VO_2max_: NA ml/kg/min	BFR cuff pressure = 180 mmHg in leg (absolute) BFR width = 10.5 cm BFR material: occlusion cuff IT protocol: 5 × 5 for three exercises at (70% 1RM) with 90s recovery, 3min inter-exercise rest	IT protocol: 5 × 5 for three exercises at (70% 1RM) with 90s recovery, 3min inter-exercise rest	SIT	3	3
Amani-Shalamzari et al., 2019	RCT	n = 12 M Age = 23 ± 2 years, Height = 174 ± 5 cm Body mass = 68 ± 7 kg well-trained futsal player Training experience ≥ 5 years VO_2max_: 44.3 ± 7.4 ml/kg/min	BFR cuff pressure = 110%–140% of SBP in leg (relative) BFR width = 13 cm BFR material: pneumatic cuff IT protocol: 4–8 × 3 min with 2 min recovery	IT protocol: 4–8 × 3 min with 2 min recovery	SSG	3–4	3
Amani-Shalamzari et al., 2020	RCT	n = 12 M Age = 23 ± 2 years, Height = 174 ± 5 cm Body mass = 68 ± 7 kg well-trained futsal player Training experience ≥ 5 years VO_2max_: 44.3 ± 7.4 ml/kg/min	BFR cuff pressure = 110%–140% of SBP in leg (relative) BFR width = 13 cm BFR material: pneumatic cuff IT protocol: 4–8 × 3 min with 2 min recovery	IT protocol: 4–8 × 3 min with 2 min recovery	SSG	3–4	3
Elgammal et al., 2020	RCT	n = 24 M Age = 22 ± 2 years, Height = 195 ± 2 cm Body mass = 81 ± 5 kg well-trained basketball player Training time ~10 hours per week VO_2max_: 38.3 ± 2.2 ml/kg/min	BFR cuff pressure = 100–160 mmHg in leg (absolute) BFR width = NA BFR material: cuffs IT protocol: 3 × 8 (30 m maximal sprints with 20s recovery), 4 min inter-exercise rest	IT protocol: 3 × 8 (30 m maximal sprints with 20s recovery), 4 min inter-exercise rest	RSS	3	4
Zhou et al., 2021	RCT	n = 34 M Age = 16 ± 1 years, Height = 171 ± 4 cm Body mass = 63 ± 8 kg trained football player Training time > 4 years VO_2max_: NA ml/kg/min	BFR cuff pressure = 210 mmHg in leg (absolute) BFR width = 5 cm BFR material: pneumatic cuff IT protocol: 6 × 3 min with 1.5 min recovery	IT protocol: 6 × 3 min with 1.5 min recovery	SSG	3	4
Hosseini Kakhak et al., 2022	RCT	n = 19 M Age = 16 ± 1 years, Height = 169 ± 8 cm Body mass = 58 ± 10 kg trained football player Training experience ≥ 3 years VO_2max_: NA ml/kg/min	BFR cuff pressure = 210 mmHg in leg (absolute) BFR width = 4 cm BFR material: pneumatic cuff IT protocol: 6 × 2 min at 75%HR_max_ with 1.5 min recovery	IT protocol: 6 × 2 min at 75%HR_max_ with 1.5 min recovery	SSG	3	6
Mckee et al., 2024	RCT	n = 26 M Age = 21 ± 5 years, Height = 182 ± 8 cm Body mass = 58 ± 10 kg trained team-sports athletes Training experience = 13 ± 3 years VO_2max_: 47.5 ± 4.2 ml/kg/min	BFR cuff pressure = 45% of AOP in leg (relative) BFR width = 10 cm BFR material: cuffs IT protocol: 3 × (4s × 5 all-out with 26s recovery), 3 min inter-exercise rest	IT protocol: 3 × (4s × 5 all-out with 26s recovery), 3 min inter-exercise rest	RSS	3	3
Xia et al., 2025	RCT	n = 30 M Age = 20 ± 1 years, Height = 179 ± 7 cm Body mass = 73 ± 3 kg trained badminton player Training experience = NA VO_2max_: NA ml/kg/min	BFR cuff pressure = 60% of AOP in leg (relative) BFR width = 5 cm BFR material: pneumatic cuff IT protocol: 4 × 8 exercises (20s with 10s recovery), 2 min inter-exercise rest	IT protocol: 4 × 8 exercises (20s work with 10s recovery), 2 min inter-exercise rest	SIT	3	6
Chen et al., 2025	RCT	n = 33 M Age = 20 ± 1 years, Height = 183 ± 4 cm Body mass = 78 ± 10 kg well-trained basketball player Training experience = NA VO_2max_: 39.6 ± 6.5 ml/kg/min	BFR cuff pressure = 180–200 mmHg in leg (absolute) BFR width = NA BFR material: cuffs IT protocol: 2 × 4 exercises (1 min work with 1 min recovery) at 70%–75% HR_max_, 2 min inter-exercises rest	IT protocol: 2 × 4 exercises (1 min work with 1 min recovery) at 85%–95% HR_max_, 2 min inter-exercises rest	LI	6	2
Xu et al., 2025	RCT	n = 36 M Age = 20 ± 1 years, Height = 174 ± 6 cm Body mass = 67 ± 10 kg well-trained boxer Training experience ≥ 10 years VO_2max_: NA ml/kg/min	BFR cuff pressure = 40% of LOP in arm (relative) BFR width = 7.5 cm BFR material: cuffs IT protocol: 3 × 14 (3s work at maximal effort with 10s recovery), 1 min inter-exercises rest	IT protocol: 3 × 14 (3s work at maximal effort with 10s recovery), 1 min inter-exercises rest	RSS	3	4
Mckee et al., 2025	RCT	n = 24 M Age = 19 ± 3 years, Height = 183 ± 7 cm Body mass = 80 ± 9 kg Trained rules footballers Training experience = 13 ± 3 years VO_2max_: NA ml/kg/min	BFR cuff pressure = 45% of AOP in leg (relative) BFR width = 10 cm BFR material: cuffs IT protocol: 3 × 5–7 (5s all-out sprint with 25 recovery), 25s inter-exercises rest	IT protocol: 3 × 5–7 (5s all-out sprint with 25 recovery), 25s inter-exercises rest	RSS	3	3
Liu et al., 2026	RCT	n = 24 M Age = 21 ± 2 years, Height = NA Body mass = NA Well-trained basketball player Training experience = NA VO_2max_: 43.3 ± 5.7 ml/kg/min	BFR cuff pressure = 100%–130% of SBP in leg (relative) BFR width = 10 cm BFR material: cuffs IT protocol: 3 × 3–4.5 min with 3 min recovery	IT protocol: 3 × 3–4.5 min with 3 min recovery	SSG	2	4

Notes: Training status: use training volume and performance metrics to classify a participant to one of the following: Tier 0: Sedentary; Tier 1: Recreationally Active; Tier 2: Trained/Developmental; Tier 3: Highly Trained/National Level; Tier 4: Elite/International Level; or Tier 5: World Class.

AOP, arterial occlusion pressure; BFR, blood flow restriction; BMI, body mass index; Fre, training frequency (sessions/week); HR_max_, maximum heart rate; IT, interval training; LI, long intervals; LOP, individualized lowest occlusion pressure; NA, not available; RCT, randomized controlled trial; RPE, rating of perceived exertion; RSS, repeated short sprints; SBP, systolic blood pressure; SIT, sprints interval training; SSG, small-side games; VO_2max_, maximum oxygen uptake; Wk, training intervention weeks.

### 3.2 Quality of study methods

**Figure 2:**
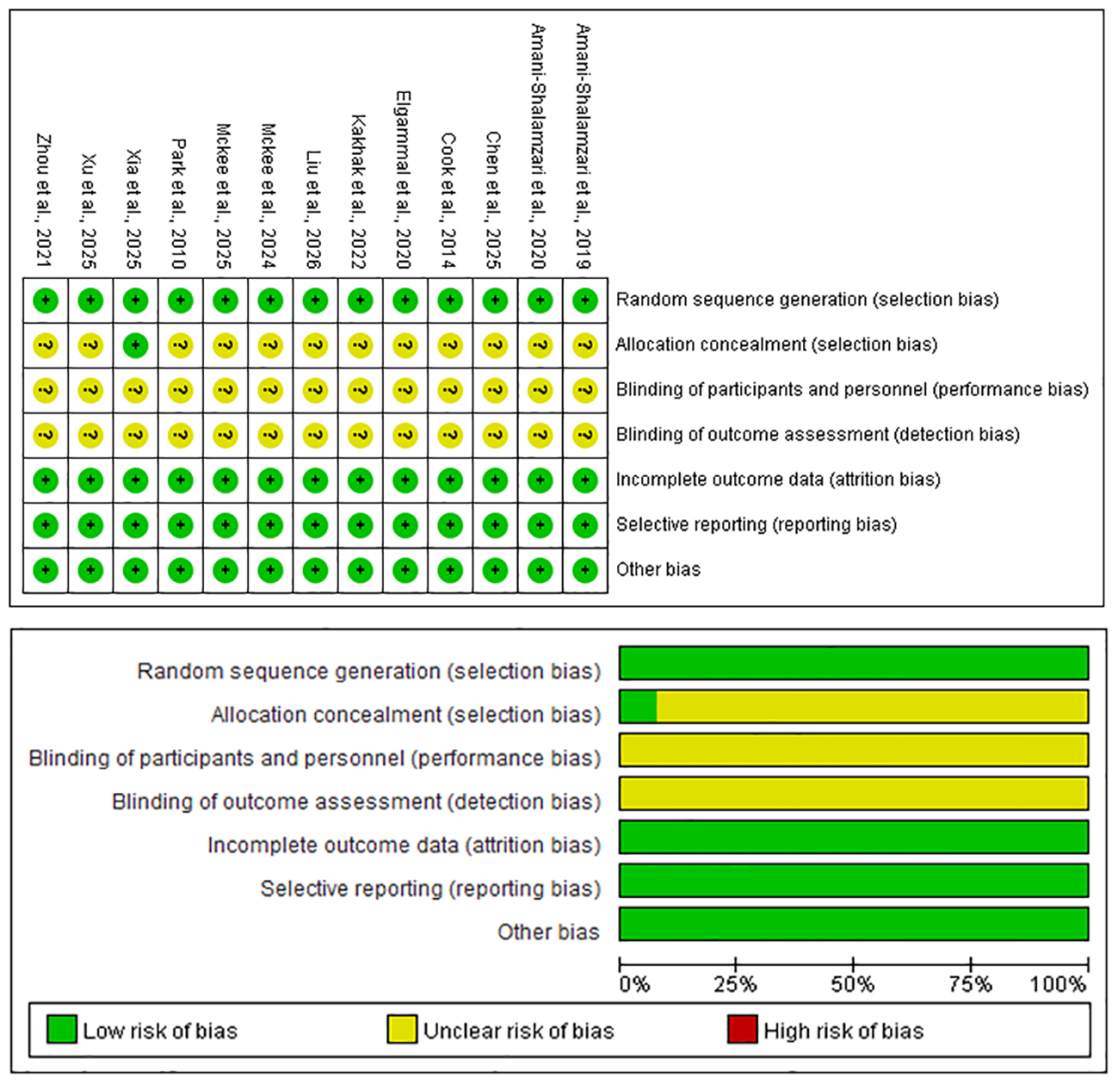
Risk of bias summary of included studies. (+) low risk of bias; ()? unclear risk of bias; (-) high risk of bias.

Risk of bias was assessed for the 13 included studies (Park et al., 2010; Cook et al., 2014; Amani-Shalamzari et al., 2019, 2020; Elgammal et al., 2020; Zhou et al., 2021; Hosseini Kakhak et al., 2022; Mckee et al., 2024, 2025; Chen et al., 2025; Xia et al., 2025; Xu et al., 2025; Liu et al., 2026) (**Figure 2**). One trial had a *low* risk of bias, while twelve were deemed to have a *moderate* risk of bias.

PEDro scores for the 13 included studies ranged from 5 to 7 (**Appendix S3**). The mean rating was 5.9, indicating that the overall study quality was fair. Out of these, two studies were rated as good and eleven studies were rated as fair. Notably, only Xia et al. (2025) reported details of allocation concealment, and none of the studies provided information regarding the blinding procedure.

The publication bias was investigated using a funnel plot combined with Egger’s test and trim-and-fill tests. Egger’s regression suggested potential publication bias for the primary pooled effect of muscle fitness, as well as for the well-trained athlete and HIIT subgroups (*p* < 0.05). However, trim-and-fill analyses showed that the pooled effects remained significant (**Appendix S7**), suggesting that the main findings were not substantially affected by publication bias.

### 3.3 Primary analysis

**Figure 3:**
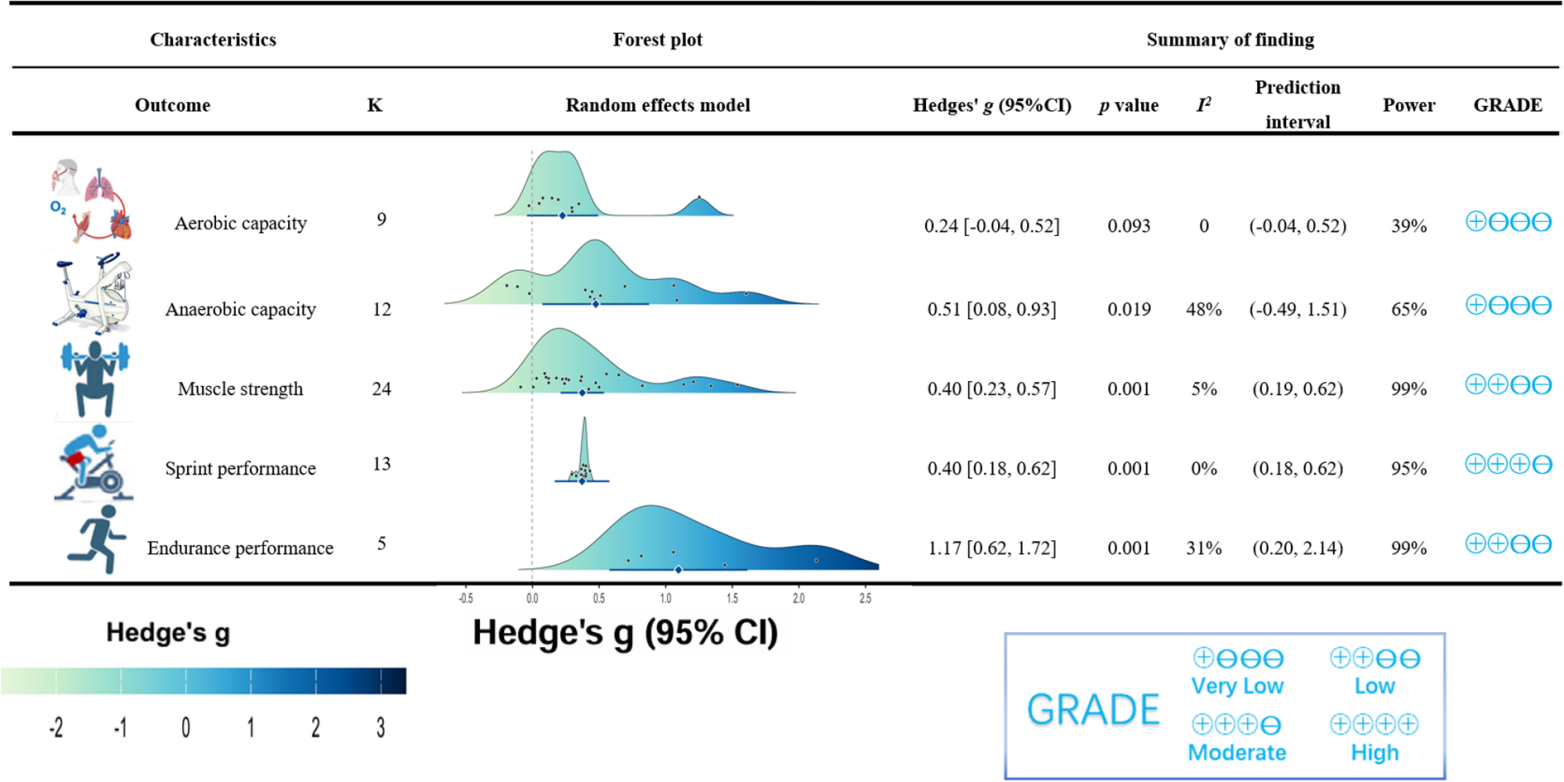
Primary pooled effect sizes for the outcomes. 95% CI, 95% confidence interval; Hedge’s *g*, the effect size indicators used in the pooled; *I^2^*, quantitative indicators of heterogeneity; K, the total number of included studies; *p* value, statistically significant *p* values for pooled results; Power, statistical power for pooled effect size.

The three-level meta-analysis showed that IT + BFR significantly improved anaerobic capacity (*g* = 0.51, 95% CI = 0.08 to 0.93, *I^2^* = 48% [*moderate*], PI: 0.08 to 0.93, *p* = 0.019, *very low* GRADE) compared with IT alone in male intermittent-sport athletes (**Figure 3**). However, there was no statistically significant difference between IT + BFR and IT alone in aerobic capacity (*g* = 0.24, 95% CI = -0.04 to 0.52, *I^2^* = 0% [*low*], PI: -0.04 to 0.52, *p* = 0.093, *very low* GRADE). Furthermore, the meta-analysis revealed a significant improvement for IT + BFR compared to IT alone in muscle fitness (*g* = 0.40, 95% CI = 0.23 to 0.57, *I^2^* = 5% [*low*], PI: -0.19 to 0.62, *p* < 0.001, *low* GRADE), sprint performance (*g* = 0.40, 95% CI = 0.18 to 0.62, *I^2^* = 0% [*low*], PI: 0.18 to 0.62, *p* < 0.001, *moderate* GRADE), and endurance performance (*g* = 1.17, 95% CI = 0.62 to 1.72, *I^2^* = 31% [*moderate*], PI: 0.20 to 2.14, *p* < 0.001, *low* GRADE).

Variance distribution across the three-level model revealed no within-study variance (Level 2: 0%) for anaerobic capacity, with heterogeneity entirely attributable to between-study differences (Level 3: 48%). In contrast, muscle fitness displayed low but non-zero variance at both the within-study (Level 2: 1%) and between-study (Level 3: 4%) levels (**Appendix S6**). Therefore, moderator analyses were conducted for anaerobic capacity to explore substantial heterogeneity and for muscle fitness to assess the potential influence of training-related factors.

### 3.4 Moderator analysis

**Figure 4:**
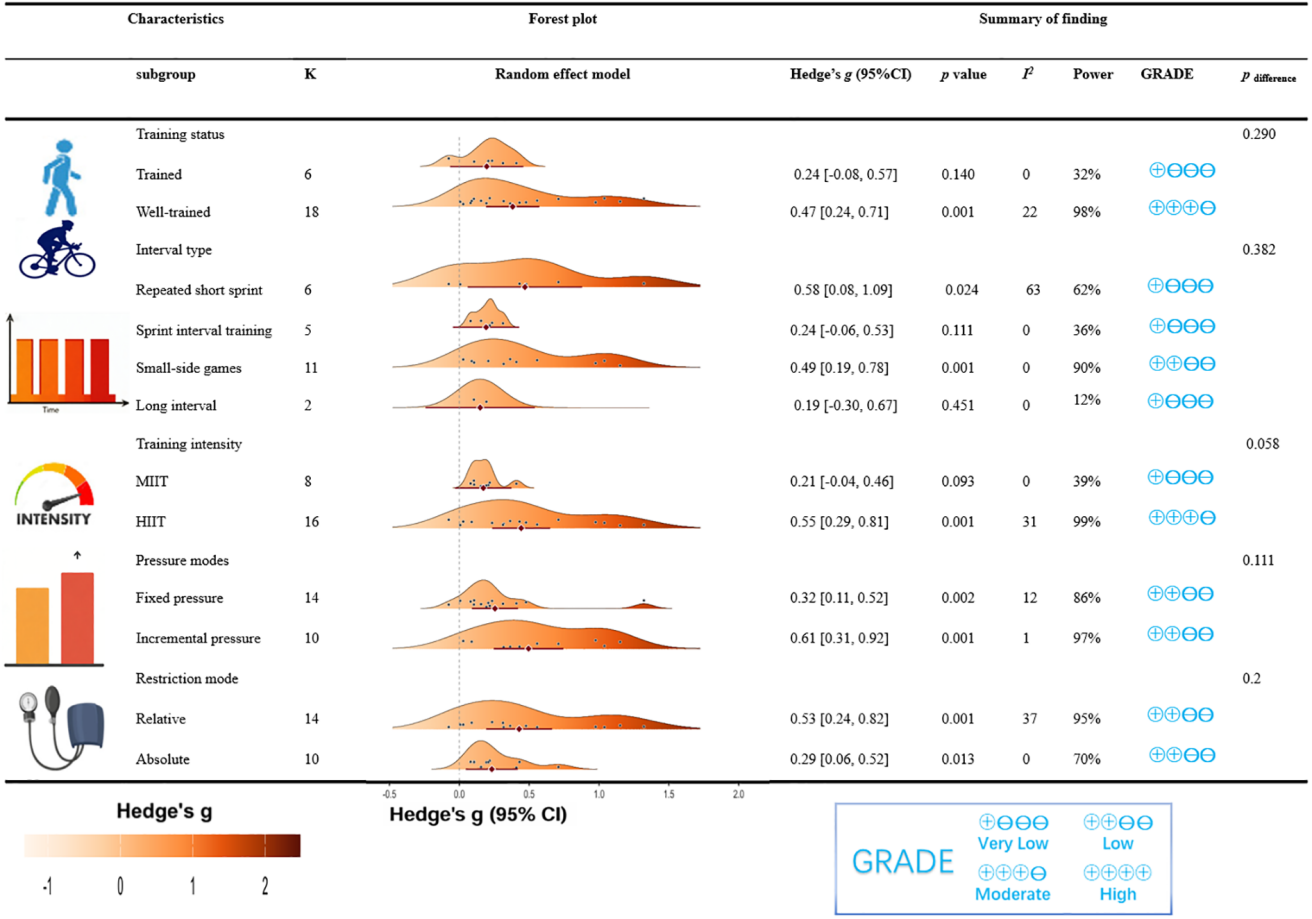
Subgroup analysis based on muscle fitness. 95% CI, 95% confidence interval; Hedge’s *g*, the effect size indicators used in the pooled; *I^2^*, quantitative indicators of heterogeneity; K, the total number of effects included in the pooled effect size; *p* value, statistically significant *p* values for pooled results; *p*_difference_, *p* value of the difference between subgroups; HIIT, high-intensity interval training; MIIT, moderate-intensity interval training.

We conducted moderator analyses to explore the modifying effects of participant characteristics and training protocol parameters on anaerobic capacity (**Appendix S4**) and muscle fitness in male intermittent-sport athletes (**Figure 4**).

#### 3.4.1 Potential moderators of anaerobic capacity

No statistically significant differences were observed in anaerobic capacity among all subgroups (all *p* for subgroup > 0.05) (**Appendix S4**). For pressure modes, IT + BFR demonstrated a significant advantage only in incremental pressure (k = 6, *g* = 0.58, 95% CI = 0.16 to 0.99, *p* = 0.007), but not in fixed pressure (k = 6, *g* = 0.44, 95% CI = -0.28 to 1.17, *p* = 0.231). In terms of restriction mode, only relative (k = 10, *g* = 0.61, 95% CI = 0.07 to 1.15, *p* = 0.028) showed significant benefits with IT + BFR, not in absolute (k = 2, *g* = 0.22, 95% CI = -0.33 to 0.76, *p* = 0.439).

**Figure 5:**
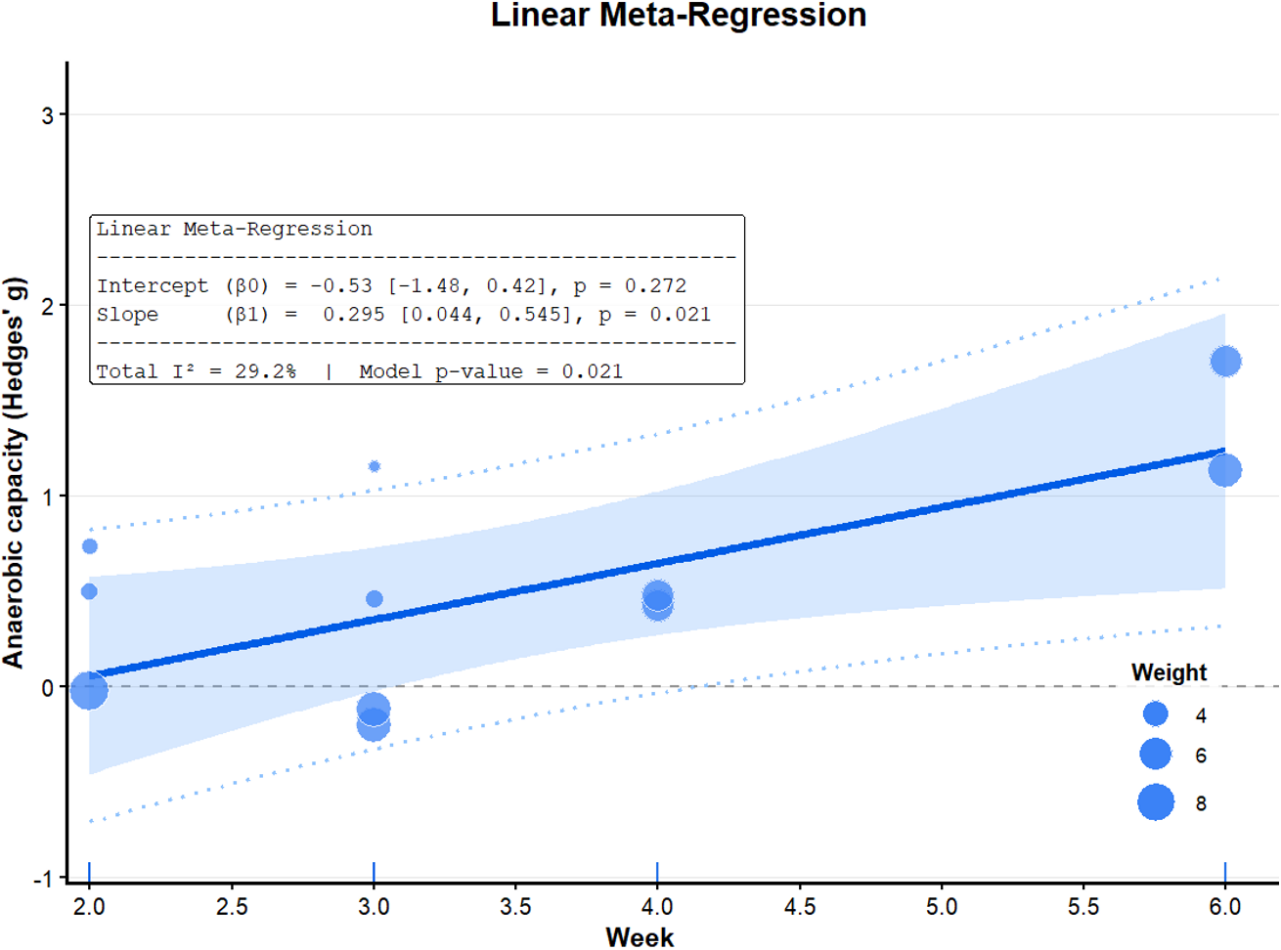
Regression analysis of total week. *β0*, the regression intercept; *β_1_*, the regression slopes; *I^2^*, heterogeneity; the blue shaded part represents the 95% confidence interval; the outermost blue dotted line represents the prediction interval; *p* value, statistically significant *p* values for regression analysis results.

The meta-regression analysis indicated that training duration significantly moderated anaerobic capacity improvements. Specifically, longer training duration was associated with greater increases in anaerobic capacity (*β* = 0.30, 95% CI = 0.04 to 0.55, *p* = 0.021) (**Figure 5**).

#### 3.4.2 Potential moderators of muscle fitness

No statistically significant differences were observed in muscle fitness among all subgroups (all *p* for subgroup > 0.05) (**Figure 4**). Regarding training status, IT + BFR was significantly superior to IT alone for muscle fitness only in well-trained athletes (k = 18, *g* = 0.47, 95% CI = 0.24 to 0.71, *p* < 0.001), but not in trained athletes (k = 6, *g* = 0.24, 95% CI = -0.08 to 0.57, *p* = 0.140). For interval type, IT + BFR was significantly superior to IT alone for muscle fitness in repeated short sprints (k = 6, *g* = 0.58, 95% CI = 0.08 to 1.09, *p* = 0.024) and SSG (k = 11, *g* = 0.49, 95% CI = 0.19 to 0.78, *p* < 0.001), but not in SIT (k = 5, *g* = 0.24, 95% CI = -0.06 to 0.53, *p* = 0.111) and long intervals (k = 2, *g* = 0.19, 95% CI = -0.39 to 0.67, *p* = 0.451). Regarding training intensity, significant improvement was observed during HIIT (k = 16, *g* = 0.55, 95% CI = 0.29 to 0.81, *p* < 0.001), but not in MIIT (k = 8, *g* = 0.21, 95% CI = -0.04 to 0.46, *p* = 0.093).

### 3.5 Sensitivity analysis

#### 3.5.1 Sensitivity analysis for primary effect

**Figure 6:**
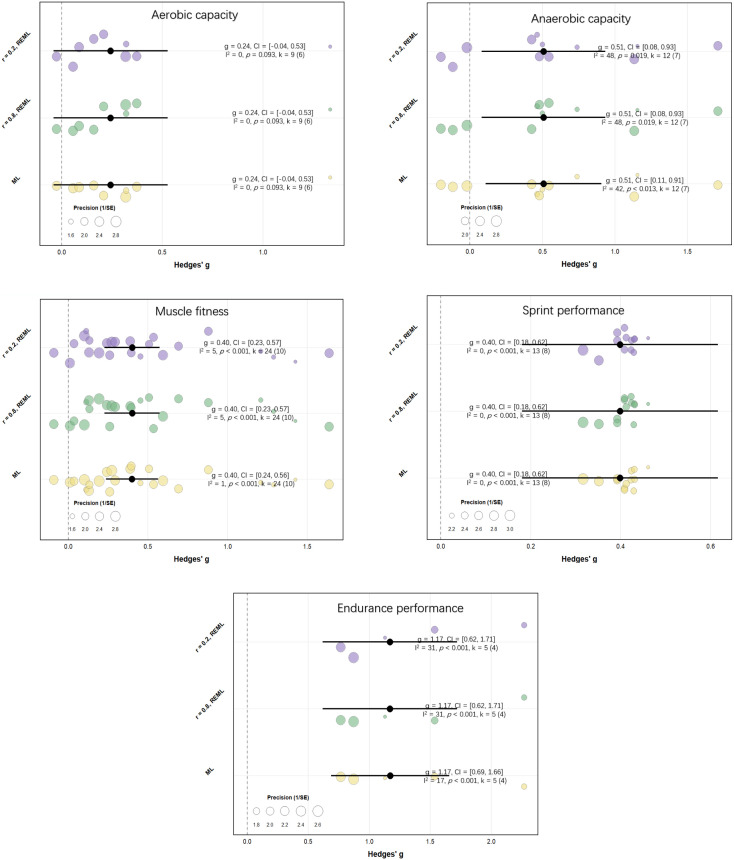
Sensitivity analysis.

Firstly, to assess the robustness of the main effect, we conducted a sensitivity analysis using two distinct values of the within-subject correlation coefficient (r) for all three-level pooled effects: a relatively low value (r = 0.2) and a relatively high value (r = 0.8). The overall effect size remained consistent in direction. The within-study correlation assumption had no impact on heterogeneity estimates, further indicating that different correlation assumptions exerted limited influence on heterogeneity estimation. Additionally, the results of the ML model were also not significantly different from those of the REML model (**Figure 6**).

We conducted sensitivity analyses using a leave-one-out method for three-level meta-analysis. The results indicated that the findings were robust across all pooled effects (**Appendix S5**). Then, our meta-analysis analyzing abnormal values using Cook’s distance and studentized residuals in the three-level meta-analysis. Regarding anaerobic capacity, Xia et al. (2025) were flagged as outliers based on Cook’s distance and studentized residuals, however, removing this study did not significantly alter the re-pooled results (*g* = 0.42, 95% CI = 0.07 to 0.78, *I^2^* = 29% [*moderate*], *p* = 0.018). Outliers identified through Cook’s distance and studentized residuals did not substantially alter the pooled results across the analyzed outcomes.

#### 3.5.2 Sensitivity analysis for moderator effect

Leave-one-out sensitivity analyses were performed for all moderator analyses, and the exclusion of certain studies led to notable changes in the pooled estimates for some outcomes (**Appendix S5**). For anaerobic capacity, removing Chen et al. (2025) (*g* = 0.56, 95% CI = 0.08 to 1.04, *p* = 0.022) changed the well-trained results from non-significant to significant. Furthermore, the removal of Mckee et al. (2024) (*g* = 0.65, 95% CI = 0.02 to 1.29, *p* = 0.043) shifted the HIIT results from non-significant to significant, whereas removing Amani-Shalamzari et al. (2020) (*g* = 0.56, 95% CI = 0.01 to 1.12, *p* = 0.053) changed the relative results from significant to non-significant. Regarding muscle fitness, excluding Xu et al. (2025) (*g* = 0.35, 95% CI = -0.43 to 1.12, *p* = 0.380) and Elgammal et al. (2020) (*g* = 0.53, 95% CI = -0.23 to 1.29, *p* = 0.172) altered the repeated short sprints results from non-significant to significant.

## 4 Discussion

The main findings suggest that IT + BFR effectively enhances physiological adaptations (anaerobic capacity and muscle fitness) and exercise performance (sprint and endurance) in male intermittent-sport athletes. Moderator analyses indicate that these effects may be influenced by training status and several training protocol parameters, including interval type, training intensity, and training duration. These factors provide valuable insights for optimizing IT + BFR protocols in male intermittent-sport athletes.

### 4.1 IT + BFR for physiological adaptations

#### 4.1.1 Aerobic capacity

Our meta-analysis showed that IT + BFR revealed no significant advantage in improving aerobic capacity compared with IT alone (*g* = 0.24, 95% CI = -0.04 to 0.52) in male intermittent-sport athletes (**Figure 3**). Although the point estimate suggested a small positive effect, the confidence interval crossed zero, indicating uncertainty regarding the true effect. This nonsignificant finding may be partly explained by the fact that most included studies employed HIIT combined with BFR (Amani-Shalamzari et al., 2020; Mckee et al., 2024, 2025; Liu et al., 2026). Previous research suggests that BFR may be particularly effective when combined with low-intensity exercise (Patterson et al., 2019), whereas HIIT combined with BFR may provide limited additional benefits for aerobic capacity when cardiovascular strain is already high (Bennett and Slattery, 2019). In support of this, studies have shown that BFR does not significantly augment the adaptive responses induced by high-intensity training (Laurentino et al., 2008; Biazon et al., 2019; Teixeira et al., 2021). Moreover, combining BFR with high-intensity exercise may increase discomfort and fatigue, potentially reducing adherence and impairing muscle coordination (Spitz et al., 2022).

Several systematic reviews have evaluated the effects of IT + BFR on aerobic capacity. For instance, Yin et al. (2025a) included 24 studies and demonstrated that IT + BFR enhanced aerobic capacity (*g* = 0.63, 95% CI = 0.28 to 0.97), particularly in trained individuals (*g* = 0.76, 95% CI = 0.44 to 1.08). Likewise, Zheng et al. (2025) reviewed 24 studies and found that IT + BFR significantly improved aerobic capacity compared to IT alone in healthy adults (*g* = 0.33, 95% CI = 0.14 to 0.51). Although previous reviews have distinguished participants based on training status, no previous meta-analysis has explored the specific effect of IT + BFR on the aerobic capacity of male intermittent-sport athletes. Our study builds on this by incorporating newer and more comprehensive literature, indicating that the addition of BFR to IT does not provide significant additional benefits for aerobic capacity within this population.

However, these findings should be viewed cautiously due to the limited statistical power of the analysis (39%) and the presence of protocol heterogeneity. Future well-designed studies with larger sample sizes and longer intervention durations are warranted to validate these preliminary results.

#### 4.1.2 Anaerobic capacity

Consistent with previous findings (Chua et al., 2022), our meta-analysis suggested that IT + BFR may improve anaerobic capacity compared with IT alone (*g* = 0.51, 95% CI = 0.08 to 0.93) in male intermittent-sport athletes (**Figure 3**). However, Zheng et al. (2025) found no significant improvement in anaerobic capacity with IT + BFR compared with IT alone in healthy adults (*g* = -0.07, 95% CI = -0.45 to 0.32). Overall, this estimate should be interpreted cautiously, given the moderate between-study heterogeneity (*I²* = 48%) and the very low certainty of the evidence. This discrepancy may be attributable to differences in participant characteristics. In particular, Zheng et al. also hypothesized that the ergogenic effects of IT + BFR may be more pronounced in trained individuals compared with untrained individuals. Mechanistically, BFR may enhance anaerobic capacity by increasing local hypoxia and metabolic stress within skeletal muscle, thereby promoting anaerobic metabolic adaptations (Christiansen, 2019). In addition, BFR optimizes substrate utilization and the intramuscular metabolic environment through enhanced GLUT4-mediated glucose uptake, expanded muscle glycogen storage, and upregulated ion transport and acid–base regulation (Cartee et al., 1991; Duncker and Bache, 2008; Christiansen et al., 2019a). Specifically, BFR increases the density of Na^+^/K^+^ pump isoforms and enhances K^+^ reuptake, thereby facilitating H^+^ efflux via Na^+^/H^+^ exchange (Christiansen et al., 2019b). Collectively, these proposed mechanisms provide a theoretical framework that warrants further investigation, but they do not constitute a robust rationale for the tentative findings of our current analysis.

Regarding training status and training intensity, neither significantly moderated the effects of IT + BFR (**Appendix S4**), suggesting that its effects may be broadly comparable across trained and well-trained intermittent-sport athletes, as well as across HIIT and MIIT combined with BFR interventions. However, the subgroup results were non-significant across all comparisons, which may be attributable to the limited sample size for anaerobic capacity and the consequently low statistical power. Notably, sensitivity analysis indicated that the findings for the well-trained and HIIT subgroups were unstable (**Appendix S5**). Therefore, these results should be interpreted with caution, and further research is warranted to confirm these findings.

Regarding pressure modes, subgroup analysis revealed no significant moderating effect (*p* for subgroup > 0.05), suggesting that both modes may be applicable across protocols. Although incremental pressure demonstrated a significant positive effect (*g* = 0.58, 95% CI = 0.16 to 0.99), no significant effect was observed for fixed pressure (*g* = 0.44, 95% CI = −0.28 to 1.17) (**Appendix S4**). However, these findings should be interpreted with caution. Despite the difference in statistical significance, the effect sizes were comparable in magnitude between the two modes. The nonsignificant result for fixed pressure was accompanied by a wide confidence interval and substantial heterogeneity, suggesting limited statistical power and the possibility of a type II error.

For restriction modes, a significant effect was observed in the relative pressure (*g* = 0.61, 95% CI = 0.07 to 1.15), whereas absolute pressure showed no significant effect (*g* = 0.22, 95% CI = -0.33 to 0.76) (**Appendix S4**). Subgroup analyses have indicated that the relative pressure may be beneficial for enhancing individual anaerobic capacity. Prescribing uniform absolute pressures fails to account for inter-individual physiological variability (Shinohara et al., 1998; Takarada et al., 2000). For example, current evidence suggests that individuals with larger limb circumferences generally require higher occlusion pressures to achieve a comparable level of vascular restriction (Loenneke et al., 2015a). In contrast, relative pressure may offer a theoretical advantage by accommodating individual differences, thereby potentially enhancing the overall efficacy of the training intervention. However, sensitivity analyses indicated that the relative pressure results were not robust. Therefore, these current findings should be interpreted with caution, and future studies are needed to definitively substantiate this comparison.

Training duration (in weeks) has been consistently identified as a critical factor influencing adaptations to both IT and BFR training (Slysz et al., 2016; MacInnis and Gibala, 2017). Similarly, Loenneke et al. (2012) observed a significant positive correlation between the effect size for strength gains and training duration in BFR protocols. Additionally, Bacon et al. (2013) found that the total number of training weeks was the strongest predictor of aerobic capacity improvement. Despite these findings, few studies have investigated the effect of different training durations on anaerobic capacity following combined IT + BFR interventions. In our study, meta-regression revealed a significant linear association between training duration and improvements in anaerobic capacity over 2–6 weeks (**Figure 5**). Physiological evidence suggests that adaptations related to anaerobic metabolism, such as enhanced blood lactate clearance and muscle glycogen utilization, require sufficient training duration to fully develop during IT (Buchheit and Laursen, 2013b; MacInnis and Gibala, 2017). Similarly, BFR-induced adaptations in strength and skeletal muscle hypertrophy may emerge as early as 3 weeks (Abe et al., 2006) but appear to be more effective after at least 6 weeks of training (Slysz et al., 2016). Nevertheless, it is important to recognize that results from meta-regression analyses are inherently observational, not causal. Therefore, randomized controlled trials with sufficient statistical power are necessary to verify these findings.

#### 4.1.3 Muscle fitness

Our meta-analysis found evidence suggesting that IT + BFR is associated with improvements in muscle fitness (*g* = 0.40, 95% CI = 0.23 to 0.57) in male intermittent-sport athletes (**Figure 3**). These findings align with a previous meta-analysis (Zheng et al., 2025), which demonstrated that IT + BFR significantly increases muscle fitness (*g* = 0.61, 95% CI = 0.36 to 0.86). Furthermore, previous reviews have indicated that BFR combined with aerobic exercise, including interval training, may enhance muscle fitness, although specific strength outcomes and pooled effect sizes were not reported (Silva et al., 2019; Chua et al., 2022). Mechanistically, these improvements are primarily driven by the amplified internal training load and profound metabolic stress induced by IT + BFR (Abe et al., 2006; Hjortshoej et al., 2023). Specifically, BFR-induced localized hypoxia, lactate accumulation, and protein breakdown stimulate the secretion of anabolic hormones, such as growth hormone (GH) and insulin-like growth factor-1 (IGF-1) (Martin et al., 2022; Hjortshoej et al., 2023). This cascade consequently activates the phosphoinositide 3-kinase/protein kinase B/mechanistic target of rapamycin (PI3K/AKT/mTOR) pathway, promoting muscle protein synthesis and satellite cell differentiation to facilitate muscle hypertrophy (Loenneke et al., 2010). Furthermore, BFR-induced local hypoxia has been proposed to stimulate angiogenesis via pathways such as VEGF, potentially enhancing muscle capillarization and thereby improving microvascular perfusion and metabolite clearance during intense exercise. Although a recent systematic review and meta-analysis supports BFR-related vascular adaptations (Maga et al., 2023), their contribution within IT + BFR protocols remains to be clarified.

Regarding training status, no significant moderating effect was observed (*p* for subgroup > 0.05), although the within-subgroup effects appeared inconsistent. Well-trained athletes (*g* = 0.47, 95% CI = 0.24 to 0.71) demonstrated significant improvements, whereas trained athletes did not (*g* = 0.24, 95% CI = -0.08 to 0.57) (**Figure 4**). To date, few meta-analyses have compared the effects of IT + BFR between trained and well-trained populations. However, several meta-analyses have primarily focused on comparisons between trained and sedentary individuals. For example, Yin et al. (2025a) reported larger effects of IT + BFR on muscle fitness in highly trained athletes (*g* = 0.96, *p* < 0.01) than in untrained individuals (*g* = 0.35, *p* = 0.54). Furthermore, Zheng et al. (2025) found that IT + BFR was less effective in improving muscle fitness among untrained individuals (*g* = 0.30, *p* = 0.230) than trained athletes (*g* = 0.79, *p* < 0.001). They further suggested that IT + BFR may effectively challenge the physiological limits of athletes with higher fitness levels, promoting physical performance gains (Zheng et al., 2025). This rationale may partly explain the differences between the two groups in the present study.

Regarding interval type, significant advantages were observed only for SSG (*g* = 1.59, *p* < 0.01) or repeated short sprints (*g* = 0.79, *p* < 0.01) when combined with BFR, but not for sprint interval training and long intervals. Compared with traditional interval training, SSG more closely reflect the competitive demands of intermittent-sport. Therefore, the stimulus induced by SSG may be more readily translated into improvements in muscle fitness. Furthermore, SSG generally involve longer durations and greater overall activity volume, allowing BFR to be maintained for longer periods. This may promote the preferential recruitment of fast-twitch (Type II) muscle fibers under elevated metabolic stress, thereby contributing to greater improvements in muscle fitness (Christiansen, 2019; Christiansen et al., 2019b; Wang et al., 2023). Repeated short sprints combined with BFR also significantly improved muscle fitness. Repeated short sprints typically consist of brief maximal sprints interspersed with short recovery intervals and therefore relies heavily on anaerobic metabolism, neuromuscular activation, and fast-twitch fiber recruitment (Yin et al., 2023). These adaptations may also be supported by an optimized local microenvironment, including enhanced thigh glucose uptake, improved muscle buffering capacity (Takarada et al., 2002; Wei et al., 2025), and optimized K^+^ regulation, which may collectively contribute to these beneficial effects (Hargreaves and Spriet, 2020). However, the point estimate in the repeated short sprints subgroup shifted from positive to negative after outlier exclusion, suggesting that this result should be interpreted with caution. By contrast, SIT combined with BFR did not show a significant advantage. SIT is characterized by ultra-short sprints and prolonged recovery intervals (Yin et al., 2025b), which may primarily stimulate central cardiopulmonary and metabolic adaptations, with limited direct effects on peripheral muscle fitness. For long-interval training, only two trials were included, which severely limits the reliability of the conclusions for this subgroup. Overall, these findings preliminarily suggest that SSG combined with BFR may be a more suitable training modality for team-sport athletes. Further high-quality studies with adequate sample sizes are needed to clarify the interaction between interval type and BFR on strength adaptations.

Training intensity is vital for stimulating muscle fitness (Yang et al., 2024; Ratamess et al., 2009). It is noteworthy that the between-subgroup difference approached statistical significance (*p* = 0.058) (**Figure 4**). Our findings indicate that HIIT significantly improved muscle fitness (*g* = 0.55, 95% CI = 0.29 to 0.81), whereas no such effect was observed at MIIT + BFR (*g* = 0.21, 95% CI = -0.04 to 0.46). This suggests that training intensity may be an important moderating factor (Yin et al., 2024). Notably, most of the included studies involved well-trained athletes (Cook et al., 2014; Amani-Shalamzari et al., 2019; Elgammal et al., 2020; Chen et al., 2025; Xu et al., 2025; Liu et al., 2026). Intermittent-sport athletes generally already have a high level of muscle fitness and a well-developed training background (Bangsbo et al., 2006), have a relatively high threshold for training-induced adaptation (Taylor et al., 2017). Therefore, conventional HIIT may be insufficient to induce further improvements in the short term, but the addition of BFR could potentially enhance training adaptations. In comparison, moderate-intensity training exerts a minimal effect on high-level athletes (Laursen and Jenkins, 2002; Iaia and Bangsbo, 2010). Therefore, even when combined with BFR, the overall additive effect may remain insufficient to overcome the athletes’ pre-existing adaptation level. Preliminary evidence suggests that high-intensity interval training combined with BFR may produce superior effects. However, as the result only approached statistical significance, it should be interpreted with caution. Future high-quality studies with adequate statistical power are needed to confirm this finding.

### 4.2 IT + BFR for exercise performance

#### 4.2.1 Sprint performance

Faster sprint speed and better repeated-sprint ability are important fitness requirements in intermittent sports (Girard et al., 2011). Our findings indicate that IT + BFR can significantly improve sprint performance (*g* = 0.40, 95% CI = 0.18 to 0.62) in intermittent-sport athletes (**Figure 3**). Importantly, these benefits do not appear to be limited to interval training alone. Individual trials indicate that integrating BFR into resistance training protocols (e.g., 5 × 5 squats at 70% 1RM for 3 weeks) provides superior enhancements in sprint times (a reduction of 0.03 s vs. 0.01s) among team-sport athletes (Cook et al., 2014). Interestingly, Chen et al. (2025) demonstrated that compared to HIIT, BFR combined with MIIT can achieve equivalent improvements. At present, proposed physiological mechanisms suggest that BFR induces the early recruitment of type II muscle fibers and increases metabolic stress. Metabolite accumulation during BFR training likely enhances type II fiber activation and accelerates fatigue (Loenneke et al., 2015b). This increased metabolic stress may elevate motor unit recruitment and muscle activation to sustain force output and compensate for reduced muscle conduction capacity, ultimately facilitating adaptations in jump and sprint performance (Wernbom et al., 2009).

#### 4.2.2 Endurance performance

IT + BFR significantly improved male intermittent-sport athletes’ endurance performance (*g* = 1.17, 95% CI = 0.62 to 1.72) compared with IT alone (**Figure 3**). Notably, this improvement was consistently observed across all included studies (Amani-Shalamzari et al., 2020; Elgammal et al., 2020; Hosseini Kakhak et al., 2022; Xia et al., 2025), suggesting that the finding is robust. This aligns with previous evidence showing that IT + BFR can enhance endurance-related outcomes in both healthy young and older adults (Bennett and Slattery, 2019). The enhancement could stem from local muscular adaptations, such as increased red cell mass and improved skeletal muscle buffer capacity (Barnes and Kilding, 2015). Importantly, all endurance tests included in the present review utilized TTE protocols (Amani-Shalamzari et al., 2020; Elgammal et al., 2020; Hosseini Kakhak et al., 2022; Xia et al., 2025). However, TTE tests employ open-loop designs, which may exaggerate effect sizes and exhibit large coefficients of variation (Wang et al., 2022). Furthermore, TTE is not a performance measure commonly used in mainstream sports. Therefore, caution is warranted when interpreting the practical significance of these improvements in endurance performance.

### 4.3 Practical applications

For coaches and practitioners working with intermittent-sport athletes, the present findings suggest that IT + BFR may be a useful strategy for enhancing anaerobic capacity, muscle fitness, sprint performance, and endurance performance when compared with IT. Regarding the target population, IT + BFR may be recommended for well-trained athletes in improving muscle fitness, as this approach may provide additional metabolic and mechanical overload to overcome their relatively high adaptive thresholds and promote further performance gains. Our analysis indicates that combining BFR with HIIT may confer greater advantages for improving muscle fitness. From a training-protocol perspective, SSG combined with BFR may represent a practically feasible option for improving muscle fitness due to its specificity to match-play demands and ease of integration into routine training. Practitioners should also consider that training duration appears to be an important factor, as longer duration was associated with greater improvements in anaerobic capacity.

### 4.4 Future research directions

Based on the findings of the present meta-analysis, several key directions for future research are warranted. First, since all included studies involved male athletes, future research should investigate IT + BFR in female intermittent-sport athletes, as sex-related physiological differences, particularly females’ greater reliance on aerobic metabolism (Ansdell et al., 2020), may result in different physiological adaptations. Second, as physiological demands vary significantly across intermittent sports (e.g., soccer vs. basketball) (Spencer et al., 2005), further research should investigate the chronic effects of IT + BFR on sport-specific performance outcomes. Third, the inconsistent reporting of BFR methodological variables, such as cuff pressure and application mode, limited our ability to fully evaluate moderating effects. Importantly, cuff pressure and width must be considered concurrently for a comprehensive understanding of their physiological impact. Therefore, future studies should aim to standardize BFR implementation in intermittent-sport athletes. Finally, although IT + BFR appears promising for intermittent-sport athletes, its safety and practical feasibility in applied training settings require further evaluation.

### 4.5 Strength and limitations

This is the first systematic review and three-level meta-analysis to comprehensively examine all performance-related outcomes from studies investigating IT + BFR on physical performance in male intermittent-sport athletes. A comprehensive literature search was conducted across four databases to ensure the inclusion of all relevant studies. Methodologically, we employed a robust three-level meta-analytic model to account for the dependency of multiple effect sizes within studies and avoid statistical double counting. Furthermore, both the overall and moderator-level results were evaluated through sensitivity analyses to assess the stability of findings. Finally, we applied the GRADE and PEDro scale to evaluate the certainty of each outcome, providing a transparent assessment of evidence quality and support decision-making.

Despite the strengths of this study, several limitations should be acknowledged. First, although meta-analysis was used to enhance statistical precision, the limited number of available studies and participants may still have reduced the power to detect small but meaningful effects, particularly in subgroup analyses. Second, sensitivity analyses showed that the pooled estimates in several subgroups changed markedly after the exclusion of individual studies, suggesting that some subgroup findings were not robust. Third, although our meta-regression identified a significant relationship, general conclusions regarding the effects of IT on male intermittent-sport athletes cannot be drawn without considering all components of the IT prescription, including exercise modality, intensity, work-to-rest structure, and participant characteristics (Viana et al., 2018). Fourth, the restriction of our search to English-language studies may have resulted in potential language bias (Afonso et al., 2024). Finally, while IT + BFR demonstrated overall ergogenic benefits for male intermittent-sport athletes, the limited number of eligible studies precluded subgroup analyses by specific sport (e.g., soccer, basketball), thereby preventing the exploration of sport-specific variations. Future studies with a larger number of trials may enable more refined, sport-specific analyses.

## 5 Conclusions

The meta-analysis shows that IT + BFR is more effective than IT alone for improving anaerobic capacity, muscle fitness, sprint performance, and endurance performance in male intermittent-sport athletes. Subgroup analyses indicated that HIIT might be associated with greater improvements in muscle fitness than MIIT, and that muscle fitness gains may be more apparent following SSG and in well-trained male intermittent-sport athletes. Furthermore, longer duration may be related to larger improvements in anaerobic capacity. Although these findings provide preliminary support for the application of IT + BFR in male intermittent-sport athletes, several moderator results were based on limited evidence or showed limited robustness. Therefore, future high-quality trials with adequate statistical power are needed to further refine individualized training prescriptions.

## Data Availability

The original contributions presented in the study are included in the article/**Supplementary Material**. Further inquiries can be directed to the corresponding author.
