# Supplementary material for: Can blood flow restriction amplify the physiological and performance benefits of interval training in male intermittent-sport athletes? a systematic review and meta-analysis
Source: Front Physiol. 2026 Jul 8;17:1864951. doi: 10.3389/fphys.2026.1864951 (PMC13388091; doi:10.3389/fphys.2026.1864951)
Supplement: Supplementary file 1 [file DataSheet1.docx]

**Can Blood Flow Restriction Amplify the Physiological and Performance benefits of Interval Training in Male Intermittent-Sport Athletes? A Systematic Review and Meta-Analysis**

Yixiang Peng^1^^†^, Lei He^1†^, Baiyu Liu^2^, Kai Xu^3^, Mingyue Yin^4^, Min Xia^5*^

^1^ Faculty of Health Sciences and Sports, Macao Polytechnic University, Macao, China.

^2^ School of Physical Education, Shanghai Normal University, Shanghai, China.

^3^ School of Athletic Performance, Shanghai University of Sport, Shanghai, China.

^4^ School of coaching, Shanghai University of Sport, Shanghai, China.

^5^ School of Physical Education, Shanghai University of Sport, Shanghai, China.

^†^ These authors contributed equally to this work.

***Correspondence:**

E-mail: [xiamin1118@163.com](mailto:xiamin1118@163.com)

**Material Contents**

| **Number** | **Material** |
| --- | --- |
| 1 | Supplementary Material Appendix S1 |
| 2 | Supplementary Material Appendix S2 (Search strategy) |
| 3 | Supplementary Material Appendix S3 (PEDro assessment) |
| 4 | Supplementary Material Appendix S4 (Moderator analysis for anaerobic capacity) |
| 5 | Supplementary Material Appendix S5 (A sensitivity analysis) |
| 6 | Supplementary Material Appendix S6 (Three-level meta-analysis) |
| 7 | Supplementary Material Appendix S7 (Funnel plot) |

**Supplementary Material Appendix S1**

| **Section/topic** | **#** | **Checklist item** | **Reported on page #** |
| --- | --- | --- | --- |
| **Can Blood Flow Restriction Amplify the Physiological and Performance Benefits of Interval Training in Male Intermittent-Sport Athletes? A Systematic Review and Meta-Analysis** | | |  |
| Title | 1 | Identify the report as a systematic review, meta-analysis, or both. | Yes |
| **ABSTRACT** | | |  |
| Structured summary | 2 | Provide a structured summary including, as applicable: background; objectives; data sources; study eligibility criteria, participants, and interventions; study appraisal and synthesis methods; results; conclusions and implications of key findings. | Yes |
| **INTRODUCTION** | | |  |
| Rationale | 3 | Describe the rationale for the review in the context of what is already known. | Yes |
| Objectives | 4 | Provide an explicit statement of questions being addressed with reference to participants, interventions, comparisons, outcomes, and study design (PICOS). | Yes |
| **METHODS** | | |  |
| Protocol and registration | 5 | Indicate if a review protocol exists, if and where it can be accessed (e.g., Web address), and, if available, provide registration information including registration number. | Yes |
| Eligibility criteria | 6 | Specify study characteristics (e.g., PICOS, length of follow-up) and report characteristics (e.g., years considered, language, publication status) used as criteria for eligibility, giving rationale. | Yes |
| Information sources | 7 | Describe all information sources (e.g., databases with dates of coverage) in the search. | Yes |
| Search | 8 | Present full electronic search strategy for at least one database, including any limits used, such that it could be repeated. | Yes |
| Study selection | 9 | State the process for selecting studies (i.e., screening, eligibility, included in systematic review, and, if applicable, included in the meta-analysis). | Yes |
| Data collection process | 10 | Describe method of data extraction from reports (e.g., piloted forms, independently, in duplicate) and any processes for obtaining and confirming data from investigators. | Yes |
| Data items | 11 | List and define all variables for which data were sought (e.g., PICOS) and any assumptions and simplifications made. | Yes |
| Risk of bias in individual studies | 12 | Describe methods used for assessing risk of bias of individual studies (including specification of whether this was done at the study), and how this information is to be used in any data synthesis. | Yes |
| Summary measures | 13 | State the principal summary measures (e.g., risk ratio, difference in means). | Yes |
| Synthesis of results | 14 | Describe the methods of handling data and combining results of studies, if done, including measures of consistency (e.g., I^2^) for each meta-analysis. | Yes |

Page 1 of 2

| **Section/topic** | **#** | **Checklist item** | **Reported on page #** |
| --- | --- | --- | --- |
| Risk of bias across studies | 15 | Specify any assessment of risk of bias that may affect the cumulative evidence (e.g., publication bias, selective reporting within studies). | Yes |
| Additional analyses | 16 | Describe methods of additional analyses (e.g., sensitivity or subgroup analyses, meta-regression), if done, indicating which were pre-specified. | Yes |
| **RESULTS** | | |  |
| Study selection | 17 | Give numbers of studies screened, assessed for eligibility, and included in the review, with reasons for exclusions at each stage, ideally with a flow diagram. | Yes |
| Study characteristics | 18 | For each study, present characteristics for which data were extracted (e.g., study size, PICOS, follow-up period) and provide the citations. | Yes |
| Risk of bias within studies | 19 | Present data on risk of bias of each study and, if available, any outcome level assessment. | Yes |
| Results of individual studies | 20 | For all outcomes considered (benefits or harms), present, for each study: (a) simple summary data for each intervention group (b) effect estimates and confidence intervals, ideally with a forest plot. | Yes |
| Synthesis of results | 21 | Present results of each meta-analysis done, including confidence intervals and measures of consistency. | Yes |
| Risk of bias across studies | 22 | Present results of any assessment of risk of bias across studies. | Yes |
| Additional analysis | 23 | Give results of additional analyses, if done (e.g., sensitivity or subgroup analyses, meta-regression [see Item 16]). | Yes |
| **DISCUSSION** | | |  |
| Summary of evidence | 24 | Summarize the main findings including the strength of evidence for each main outcome; consider their relevance to key groups (e.g., healthcare providers, users, and policy makers). | Yes |
| Limitations | 25 | Discuss limitations at study and outcome level (e.g., risk of bias), and at review-level (e.g., incomplete retrieval of identified research, reporting bias). | Yes |
| Conclusions | 26 | Provide a general interpretation of the results in the context of other evidence, and implications for future research. | Yes |
| **FUNDING** | | |  |
| Funding | 27 | Describe sources of funding for the systematic review and other support (e.g., supply of data); role of funders for the systematic review. | Yes |

*From:*  Moher D, Liberati A, Tetzlaff J, Altman DG, The PRISMA Group (2009). Preferred Reporting Items for Systematic Reviews and Meta-Analyses: The PRISMA Statement. PLoS Med 6(7): e1000097. doi:10.1371/journal.pmed1000097

**Supplementary Material Appendix S2 (Search strategy)**

| Data | Query | Results |
| --- | --- | --- |
| PubMed | ("blood flow restriction"[Title/Abstract] OR "blood-flow restriction"[Title/Abstract] OR "blood flow restricted"[Title/Abstract] OR "blood-flow restricted"[Title/Abstract] OR BFR[Title/Abstract] OR BFRT[Title/Abstract] OR BFRE[Title/Abstract] OR "occlusion training"[Title/Abstract] OR "vascular occlusion"[Title/Abstract] OR "partial vascular occlusion"[Title/Abstract] OR "vascular occlusion training"[Title/Abstract] OR kaatsu[Title/Abstract] OR "ischemic training"[Title/Abstract] OR "ischaemic training"[Title/Abstract] OR tourniquet*[Title/Abstract]) AND ("interval training"[Title/Abstract] OR "high-intensity interval"[Title/Abstract] OR "high intensity interval"[Title/Abstract] OR HIIT[Title/Abstract] OR HIIE[Title/Abstract] OR "sprint interval"[Title/Abstract] OR SIT[Title/Abstract] OR "repeated sprint*"[Title/Abstract] OR "repeated-sprint*"[Title/Abstract] OR RST[Title/Abstract] OR RSE[Title/Abstract] OR "intermittent training"[Title/Abstract] OR "intermittent exercise"[Title/Abstract] OR "intermittent running"[Title/Abstract] OR "intermittent sport*"[Title/Abstract] OR "match-play"[Title/Abstract] OR "team sport*"[Title/Abstract] OR "racket sport*"[Title/Abstract] OR "combat sport*"[Title/Abstract] OR soccer[Title/Abstract] OR football[Title/Abstract] OR basketball[Title/Abstract] OR rugby[Title/Abstract] OR futsal[Title/Abstract] OR handball[Title/Abstract] OR volleyball[Title/Abstract] OR tennis[Title/Abstract] OR badminton[Title/Abstract] OR hockey[Title/Abstract] OR running[Title/Abstract] OR cycling[Title/Abstract] OR sprinting[Title/Abstract] OR rowing[Title/Abstract]) | 370 |
| WOS | TS = ("blood flow restriction" OR "blood-flow restriction" OR "blood flow restricted" OR "blood-flow restricted" OR BFR OR BFRT OR BFRE OR "occlusion training" OR "vascular occlusion" OR "partial vascular occlusion" OR "vascular occlusion training" OR kaatsu OR "ischemic training" OR "ischaemic training" OR tourniquet*) AND TS = ("interval training" OR "high-intensity interval" OR "high intensity interval" OR HIIT OR HIIE OR "sprint interval" OR SIT OR "repeated sprint*" OR "repeated-sprint*" OR RST OR RSE OR "intermittent training" OR "intermittent exercise" OR "intermittent running" OR "intermittent sport*" OR "match-play" OR "team sport*" OR "racket sport*" OR "combat sport*" OR soccer OR football OR basketball OR rugby OR futsal OR handball OR volleyball OR tennis OR badminton OR hockey OR running OR cycling OR sprinting OR rowing) | 769 |
| Scopus | TITLE-ABS-KEY ("blood flow restriction" OR "blood-flow restriction" OR "blood flow restricted" OR "blood-flow restricted" OR BFR OR BFRT OR BFRE OR "occlusion training" OR "vascular occlusion" OR "partial vascular occlusion" OR "vascular occlusion training" OR kaatsu OR "ischemic training" OR "ischaemic training" OR tourniquet*) AND TITLE-ABS-KEY ("interval training" OR "high-intensity interval" OR "high intensity interval" OR HIIT OR HIIE OR "sprint interval" OR SIT OR "repeated sprint*" OR "repeated-sprint*" OR RST OR RSE OR "intermittent training" OR "intermittent exercise" OR "intermittent running" OR "intermittent sport*" OR "match-play" OR "team sport*" OR "racket sport*" OR "combat sport*" OR soccer OR football OR basketball OR rugby OR futsal OR handball OR volleyball OR tennis OR badminton OR hockey OR running OR cycling OR sprinting OR rowing) | 547 |
| Cochrane | ("blood flow restriction" OR "blood-flow restriction" OR "blood flow restricted" OR "blood-flow restricted" OR BFR OR BFRT OR BFRE OR "occlusion training" OR "vascular occlusion" OR "partial vascular occlusion" OR "vascular occlusion training" OR kaatsu OR "ischemic training" OR "ischaemic training" OR tourniquet*):ti,ab,kw AND ("interval training" OR "high-intensity interval" OR "high intensity interval" OR HIIT OR HIIE OR "sprint interval" OR SIT OR "repeated sprint*" OR "repeated-sprint*" OR RST OR RSE OR "intermittent training" OR "intermittent exercise" OR "intermittent running" OR "intermittent sport*" OR "match-play" OR "team sport*" OR "racket sport*" OR "combat sport*" OR soccer OR football OR basketball OR rugby OR futsal OR handball OR volleyball OR tennis OR badminton OR hockey OR running OR cycling OR sprinting OR rowing):ti,ab,kw | 605 |
| CNKI | SU=("血流限制" + "加压训练" + "血液阻断" + "缺血训练" + "止血带" + "BFR" + "KAATSU") * SU=("间歇训练" + "高强度间歇" + "HIIT" + "冲刺间歇" + "SIT" + "反复冲刺" + "重复冲刺" + "间歇运动" + "间歇性运动" + "团队项目" + "同场对抗" + "隔网对抗" + "重竞技" + "格斗" + "足球" + "篮球" + "排球" + "橄榄球" + "手球" + "网球" + "羽毛球" + "曲棍球" + "跑步" + "短跑" + "骑行" + "赛艇") | 418 |

| **Supplementary Material Appendix S3 (PEDro assessment)** | | | | | | | | | | | | | |  |
| --- | --- | --- | --- | --- | --- | --- | --- | --- | --- | --- | --- | --- | --- | --- |
| Study | Item 1 | Item 2 | Item 3 | Item 4 | Item 5 | Item 6 | Item 7 | Item 8 | Item 9 | Item 10 | Item 11 | Total | Rating | |
| Park et al. 2010 | YES | 1 | 0 | 1 | 0 | 0 | 0 | 1 | 1 | 1 | 1 | 6 | Fair | |
| Cook et al. 2014 | NO | 1 | 0 | 1 | 0 | 0 | 0 | 1 | 1 | 1 | 1 | 6 | Fair | |
| Amani-Shalamzari et al. 2019 | NO | 1 | 0 | 1 | 0 | 0 | 1 | 1 | 1 | 1 | 1 | 7 | Good | |
| Amani-Shalamzari et al. 2020 | NO | 1 | 0 | 1 | 0 | 0 | 0 | 1 | 1 | 0 | 1 | 5 | Fair | |
| Zhou et al. 2021 | YES | 1 | 0 | 1 | 0 | 0 | 0 | 1 | 1 | 1 | 1 | 6 | Fair | |
| Kakhak et al. 2022 | YES | 1 | 0 | 1 | 0 | 0 | 0 | 1 | 1 | 0 | 1 | 5 | Fair | |
| Elgammal et al. 2020 | YES | 1 | 0 | 1 | 0 | 0 | 0 | 1 | 1 | 1 | 1 | 6 | Fair | |
| Mckee et al. 2023 | YES | 1 | 0 | 1 | 0 | 0 | 0 | 1 | 1 | 1 | 1 | 6 | Fair | |
| Xia et al. 2025 | NO | 1 | 1 | 1 | 0 | 0 | 0 | 1 | 1 | 1 | 1 | 7 | Good | |
| Chen et al. 2025 | YES | 1 | 0 | 1 | 0 | 0 | 0 | 1 | 1 | 0 | 1 | 5 | Fair | |
| Xu et al. 2025 | NO | 1 | 0 | 1 | 0 | 0 | 0 | 1 | 1 | 0 | 1 | 5 | Fair | |
| Mckee et al. 2025 | NO | 1 | 0 | 1 | 0 | 0 | 0 | 1 | 1 | 1 | 1 | 6 | Fair | |
| Liu et al. 2025 | NO | 1 | 0 | 1 | 0 | 0 | 0 | 1 | 1 | 1 | 1 | 6 | Fair | |

Note: “excellent” (10–11 points); “good” (7–9 points); “fair” (5–6 points); and “poor” (0–4 points)

1. eligibility criteria were specified

2. subjects were randomly allocated to groups (in a crossover study, subjects were randomly allocated an order in which treatments were received)

3. allocation was concealed

4. the groups were similar at baseline regarding the most important prognostic indicators

5. there was blinding of all subjects

6. there was blinding of all therapists who administered the therapy

7. there was blinding of all assessors who measured at least one key outcome

8. measures of at least one key outcome were obtained from more than 85% of the subjects initially allocated to groups

9. all subjects for whom outcome measures were available received the treatment or control condition as allocated or, where this was not the case, data for at least one key outcome was analyzed by “intention to treat”

10. the results of between-group statistical comparisons are reported for at least one key outcome

11. the study provides both point measures and measures of variability for at least one key outcome

**
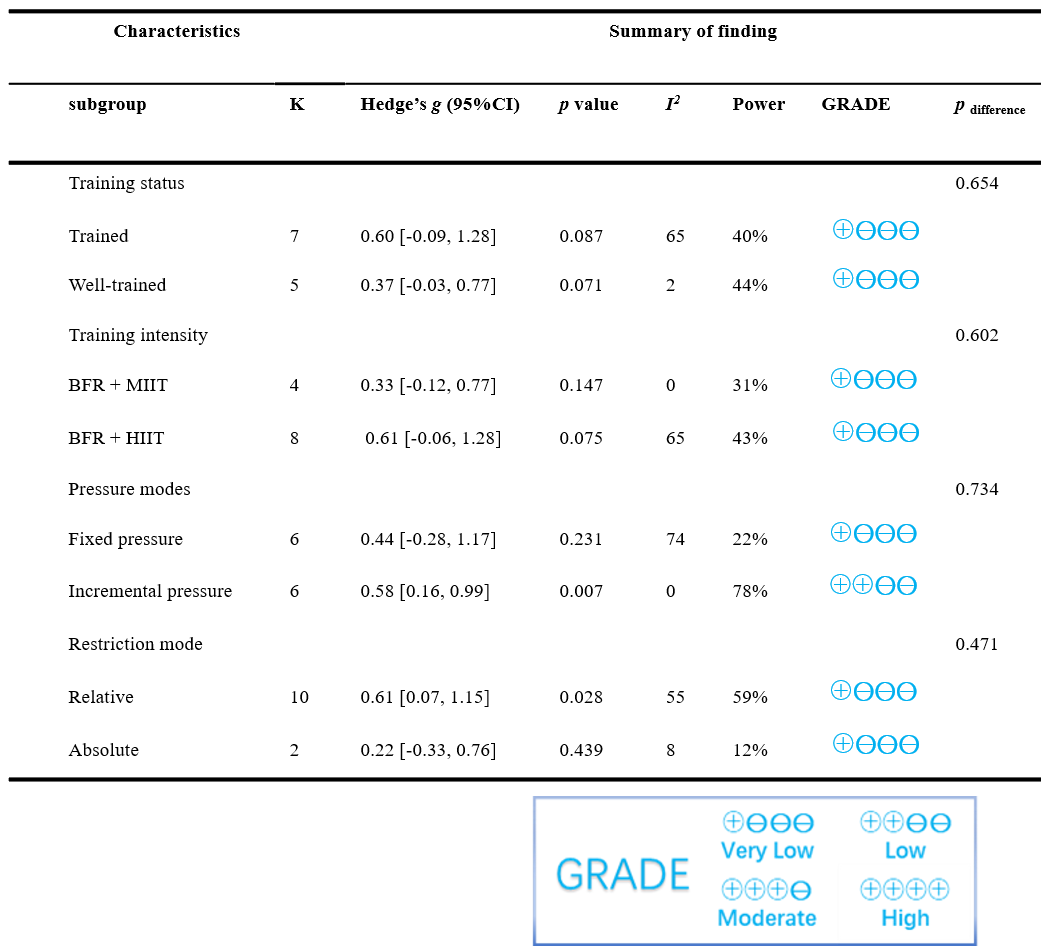
Supplementary Material Appendix S4 (Moderator analysis for anaerobic capacity)**

**Supplementary Material Appendix** **S5 (A sensitivity analysis)**

| 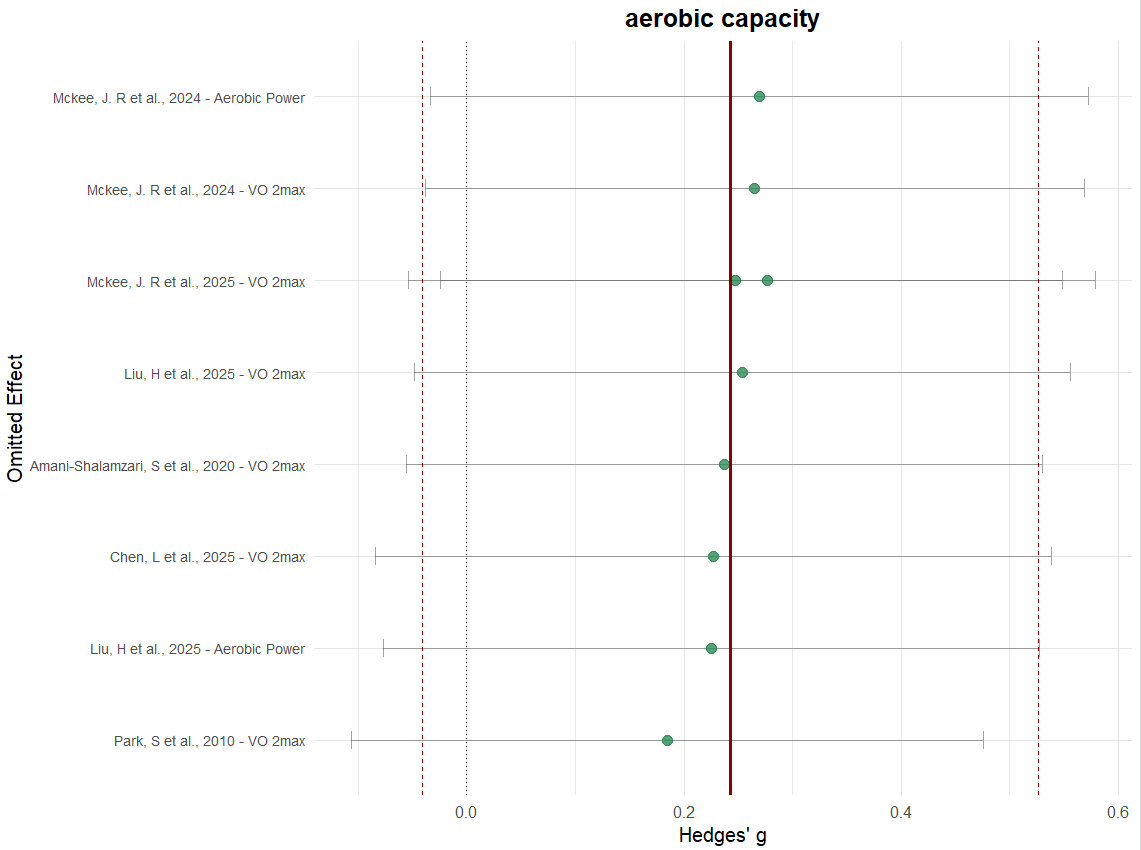 | 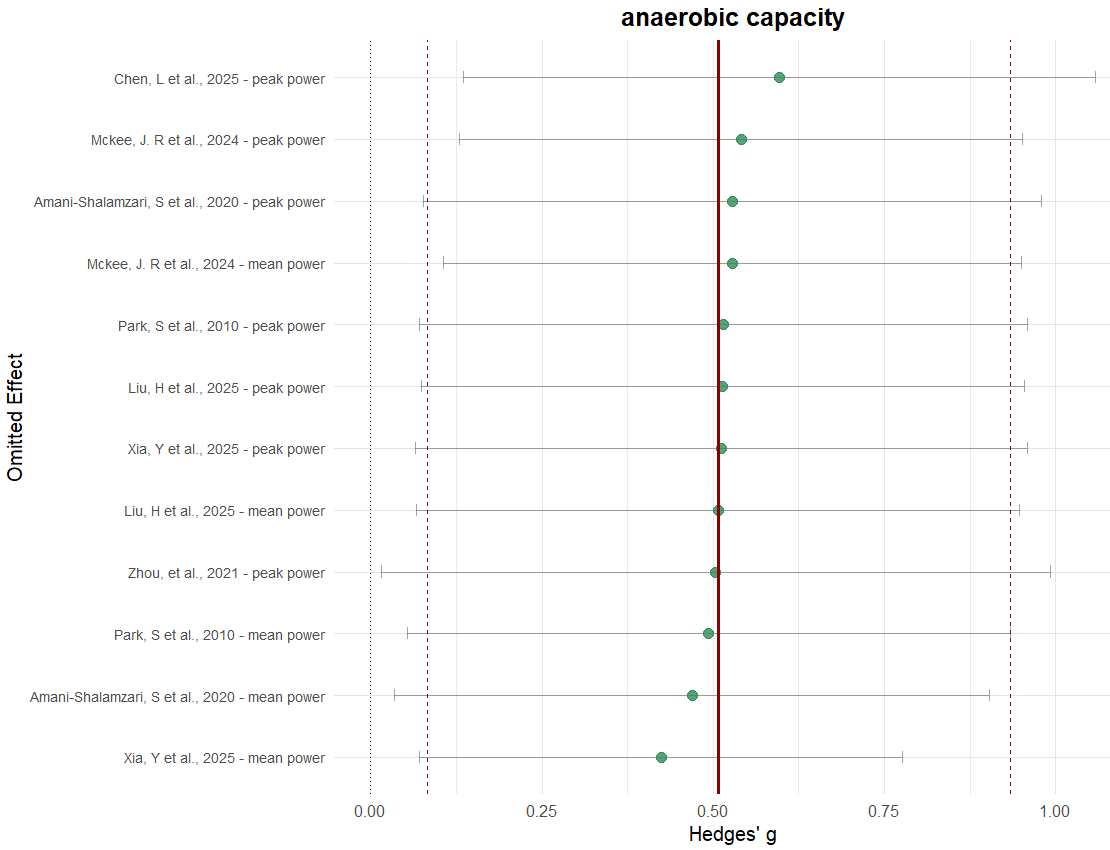 | 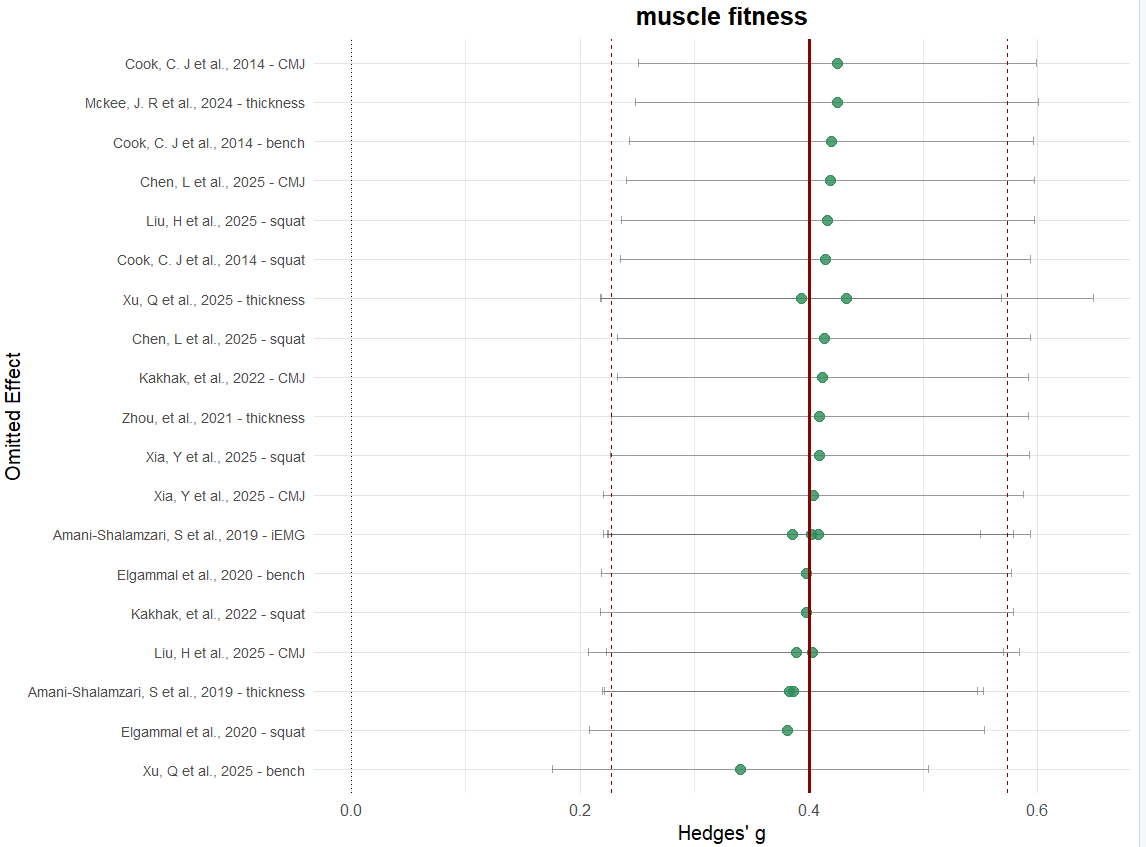 | 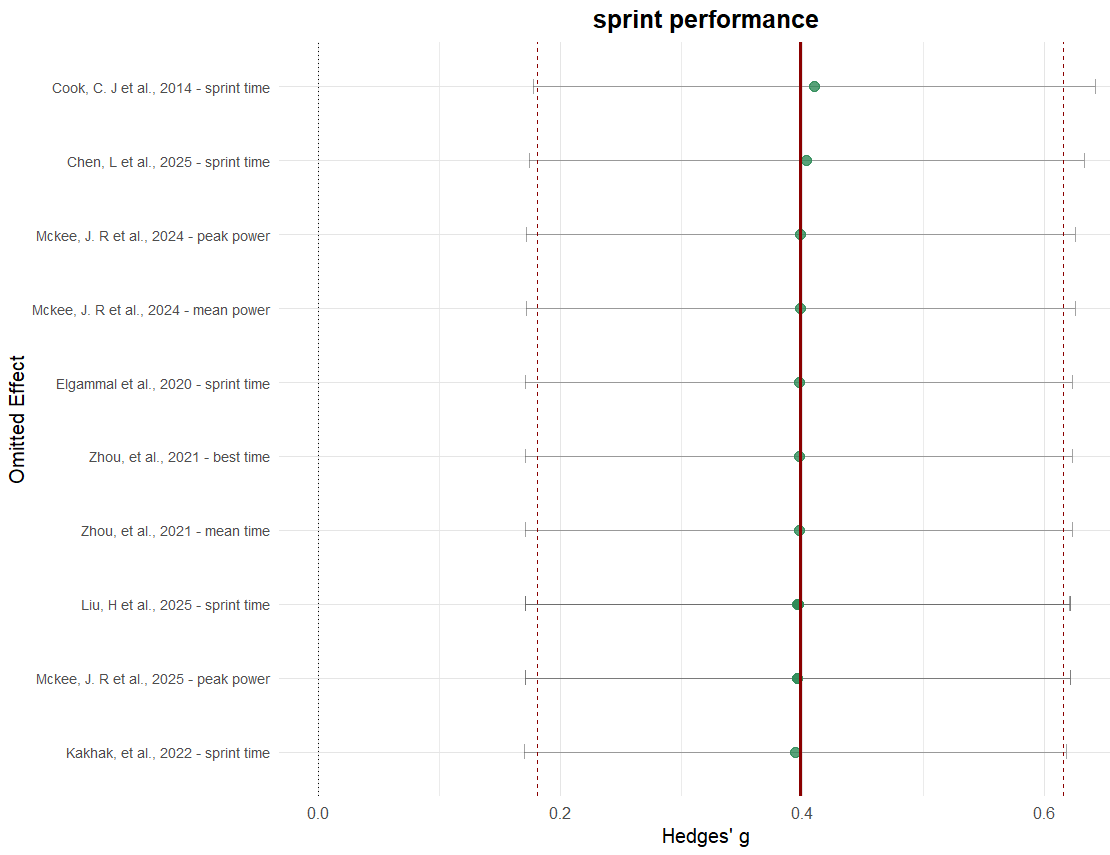 | 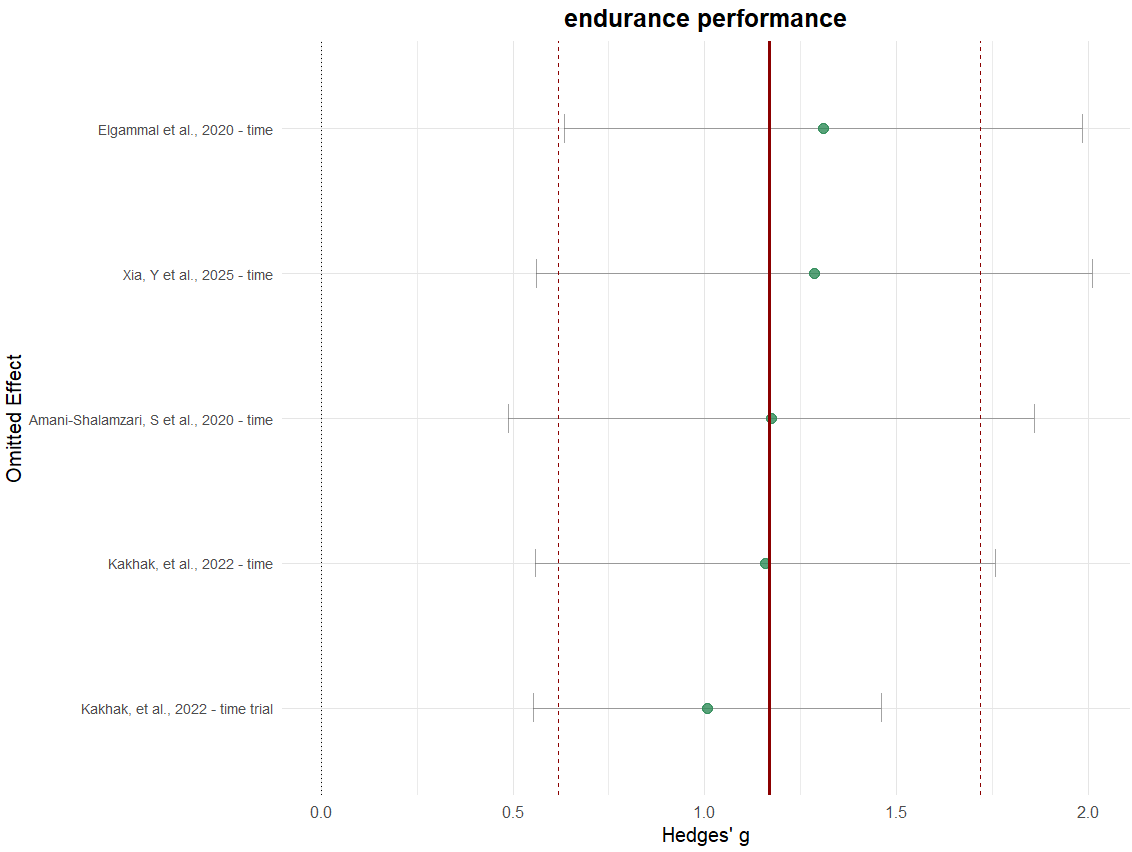 |
| --- | --- | --- | --- | --- |
| 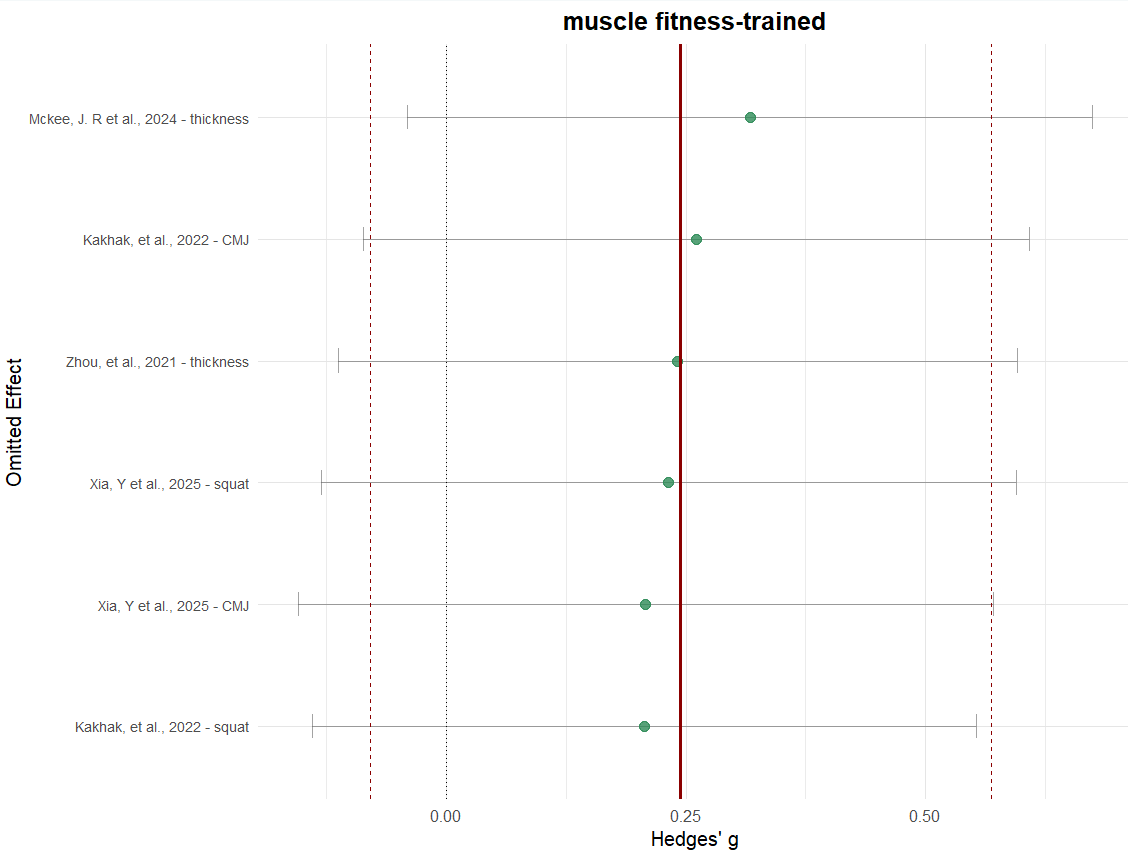 | 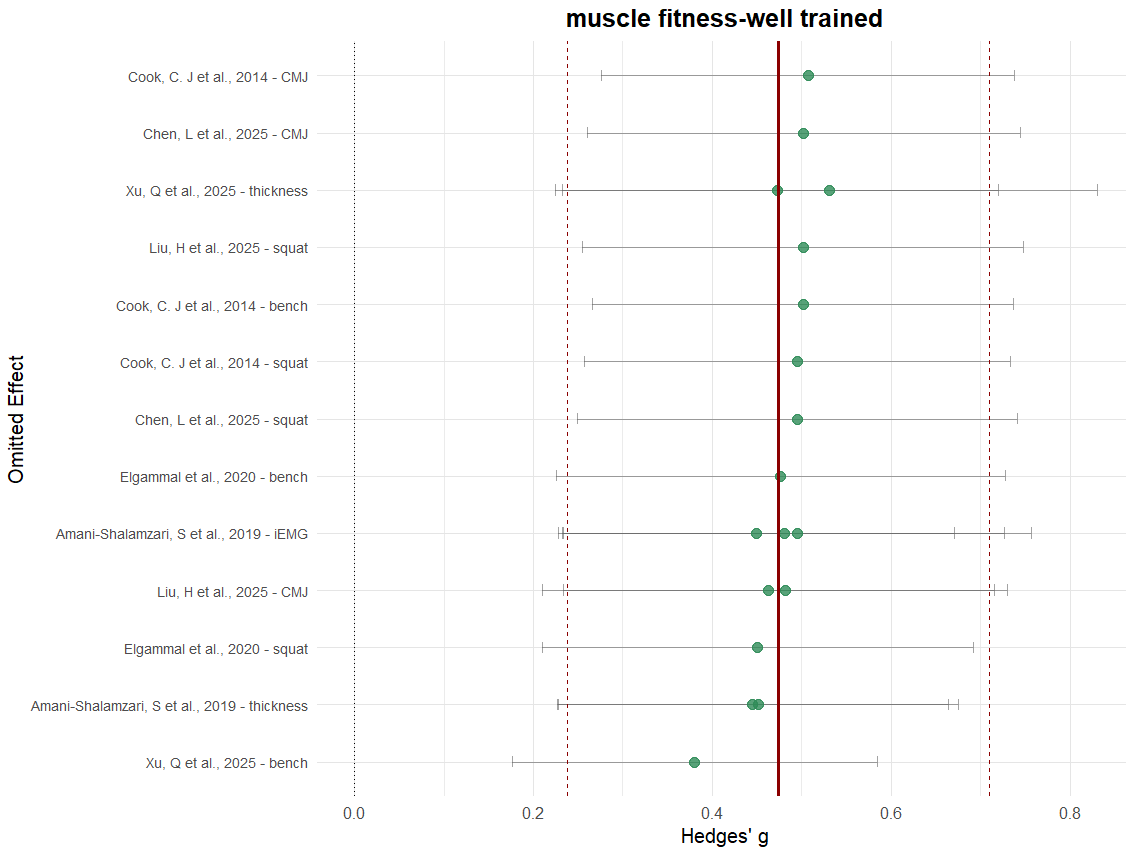 | 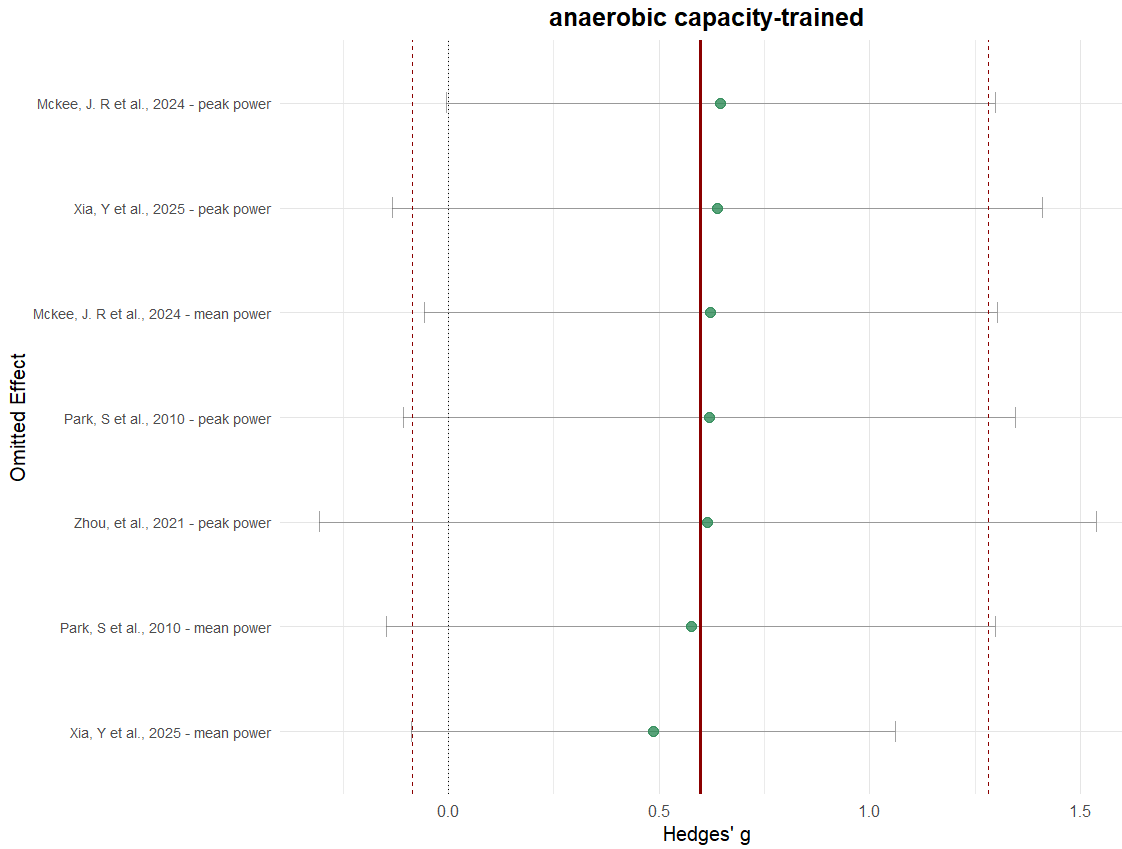 | 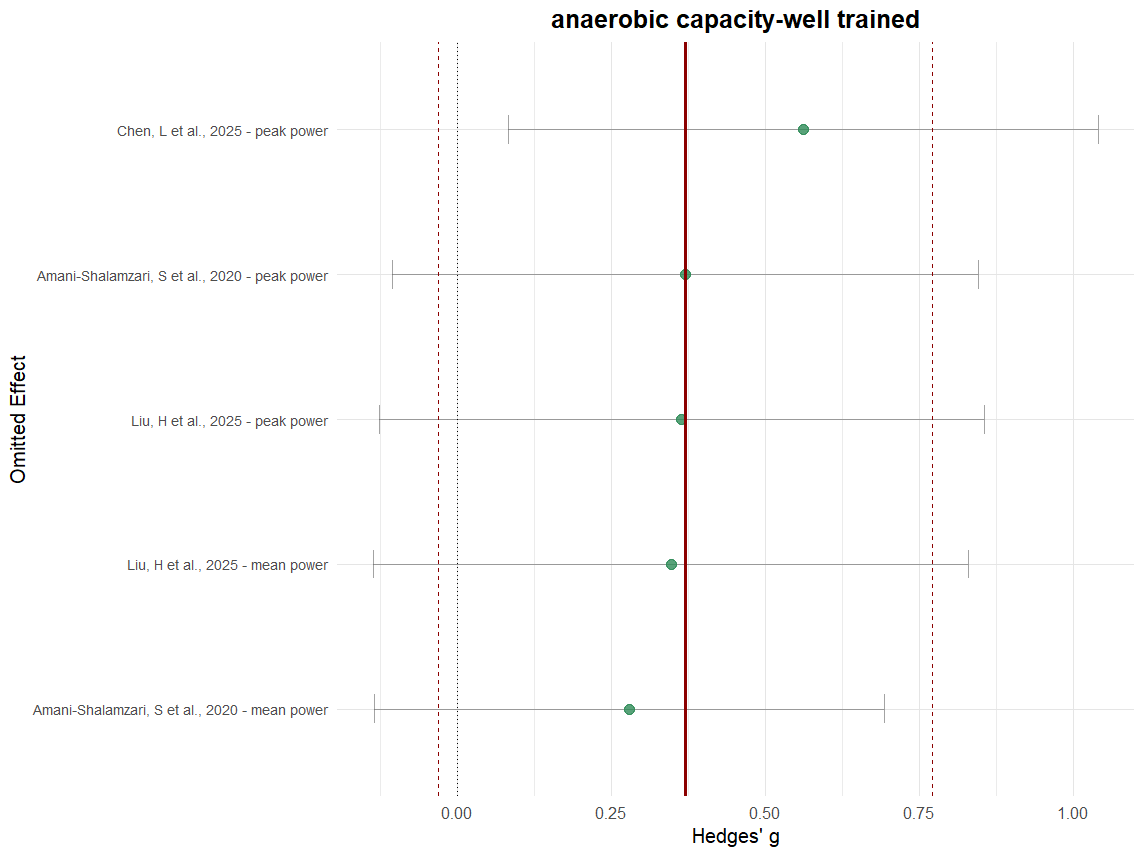 |  |
| 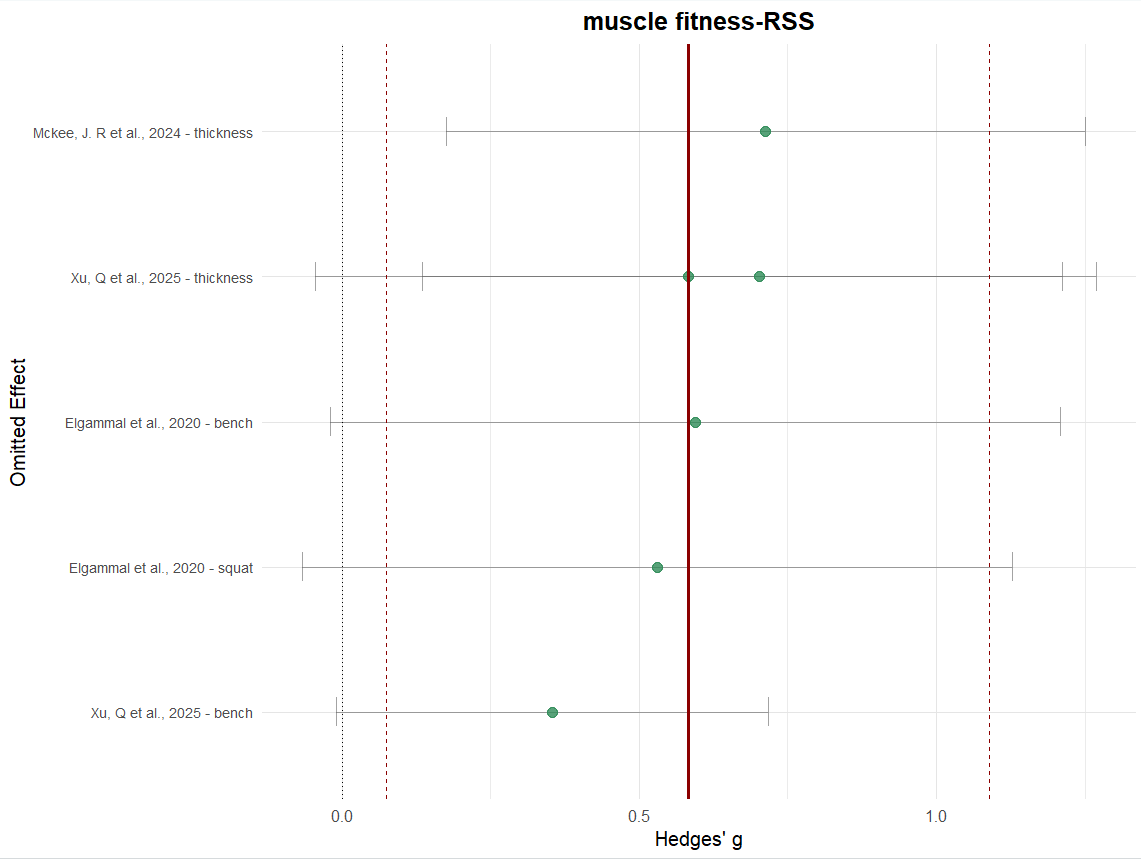 | 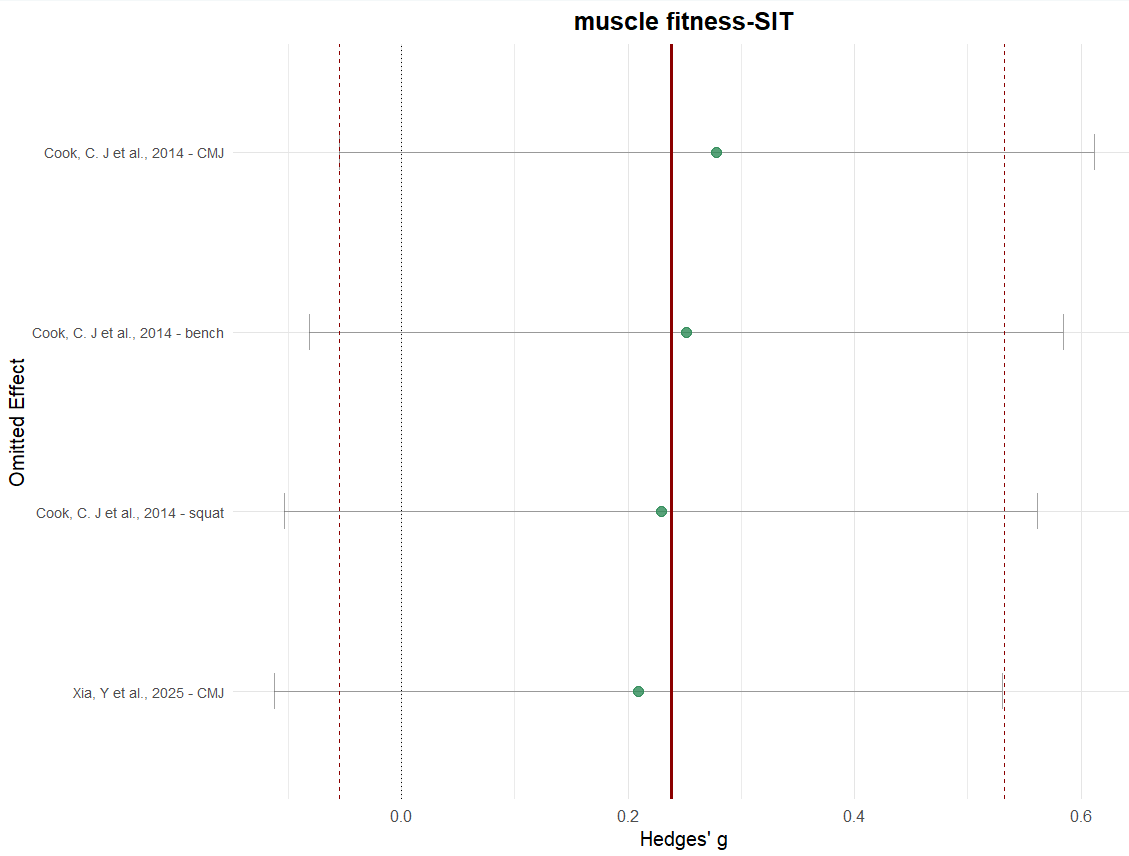 | 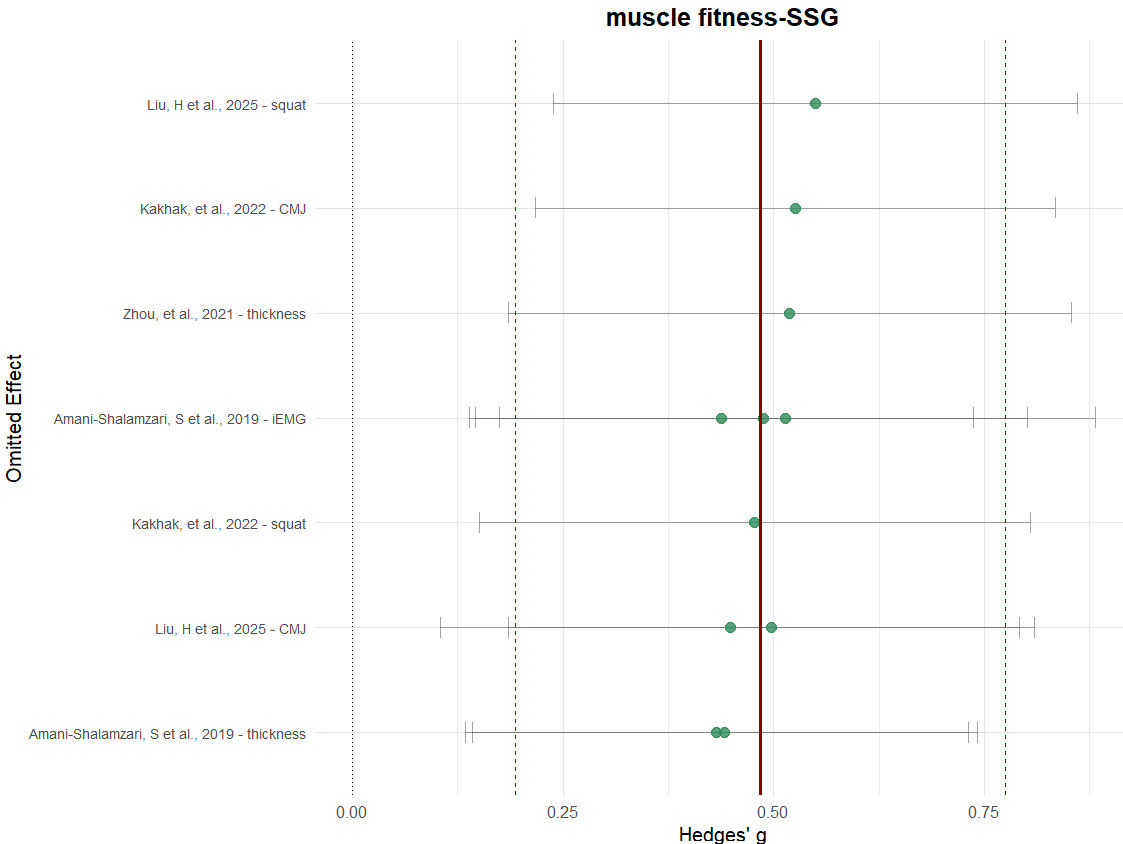 | 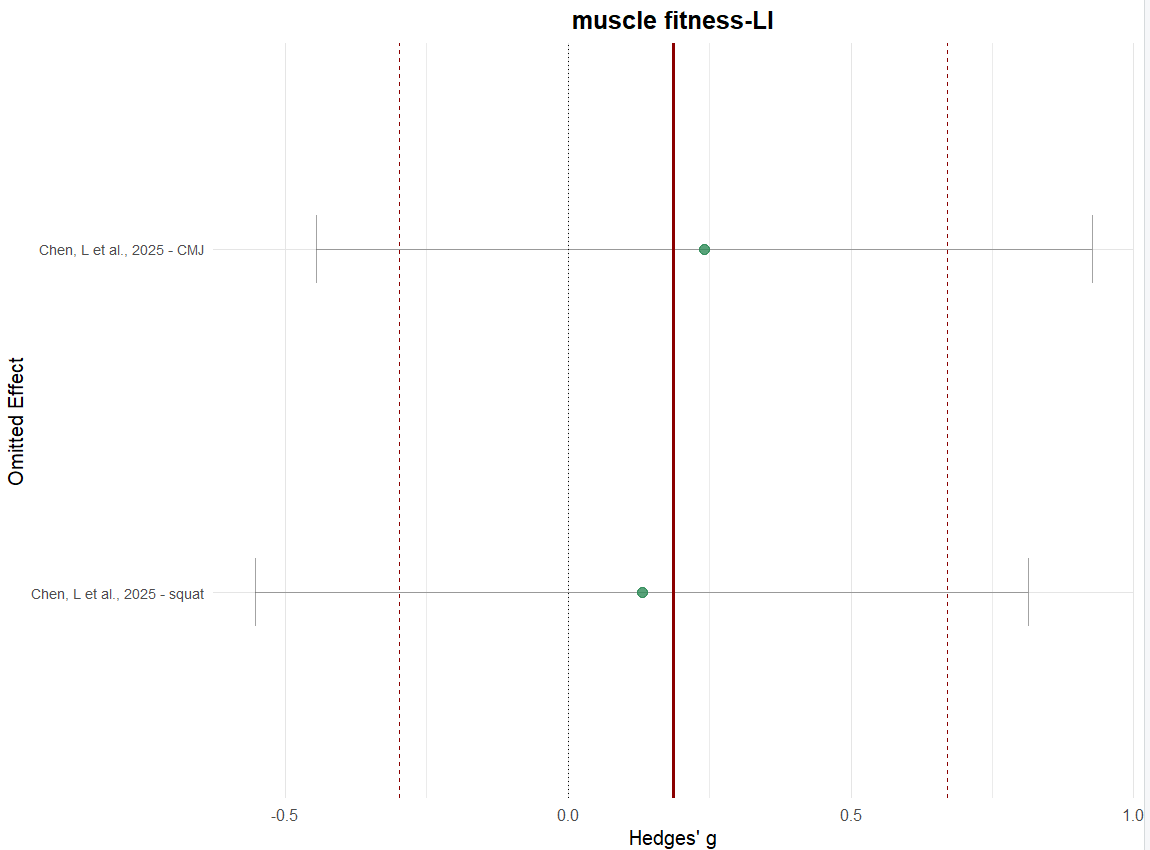 |  |
| 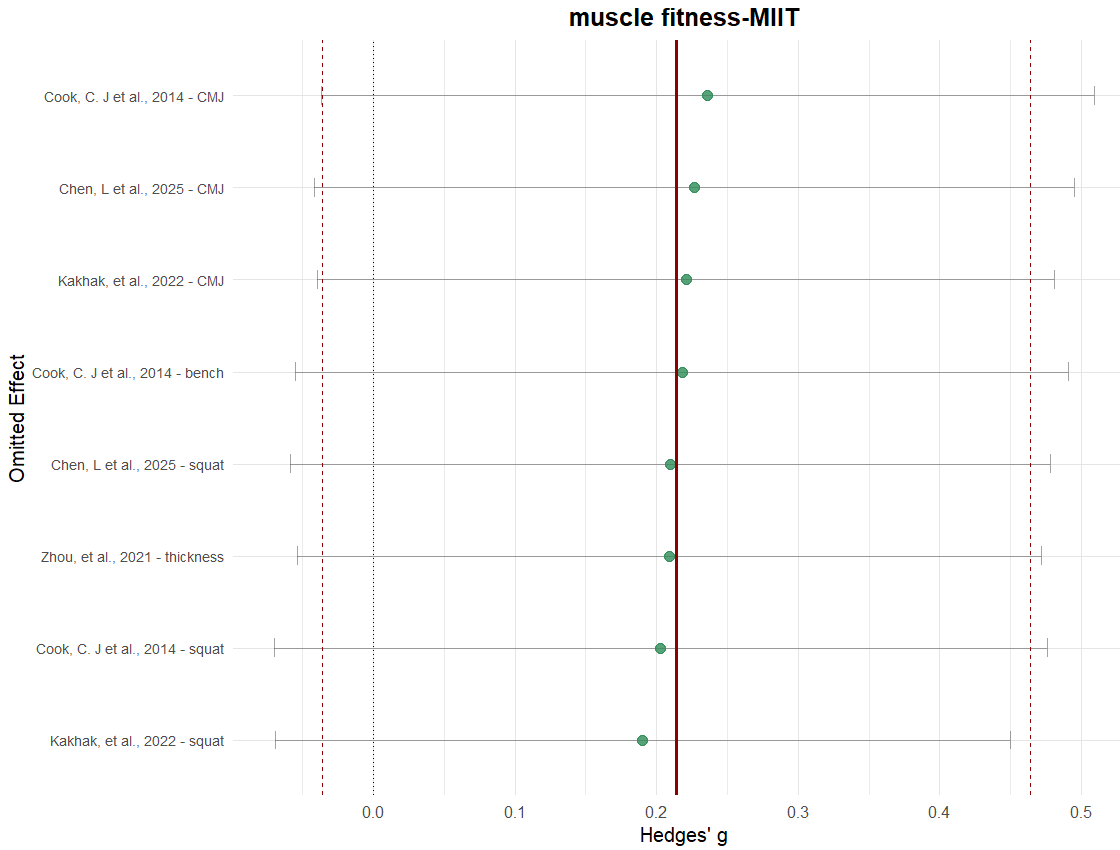 | 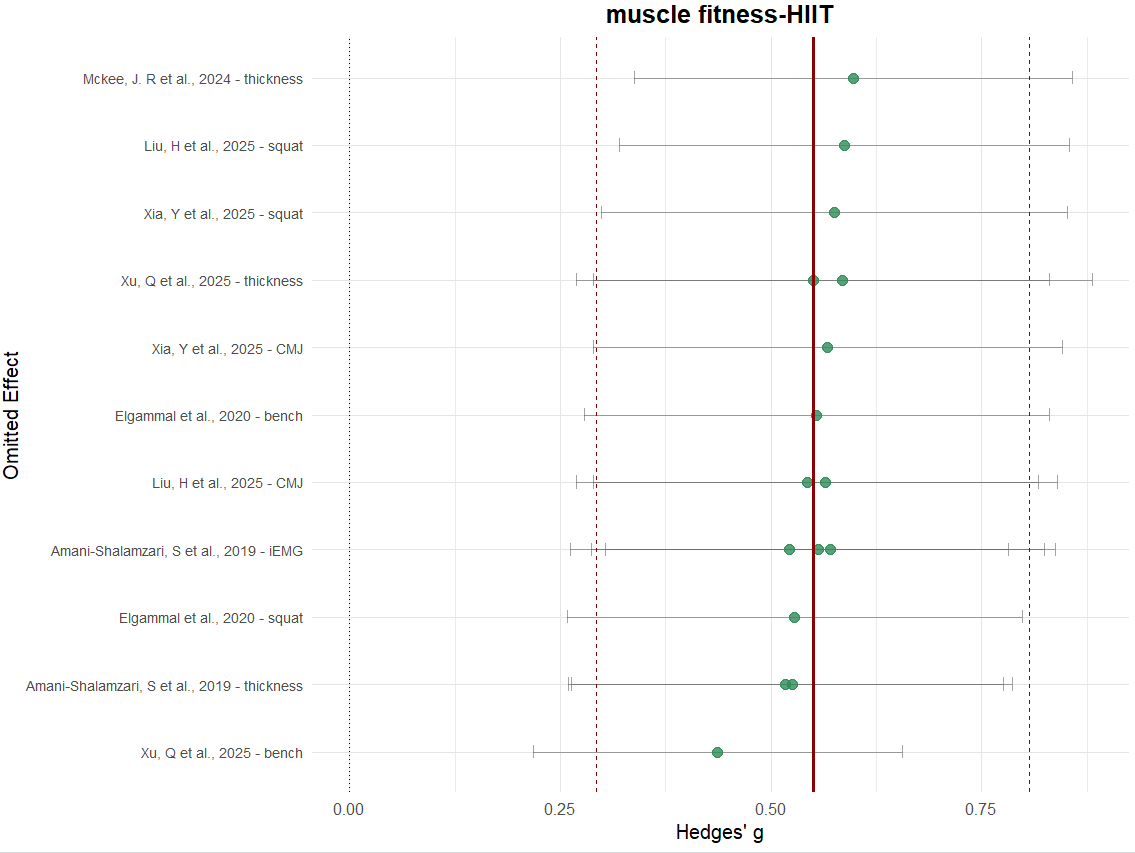 | 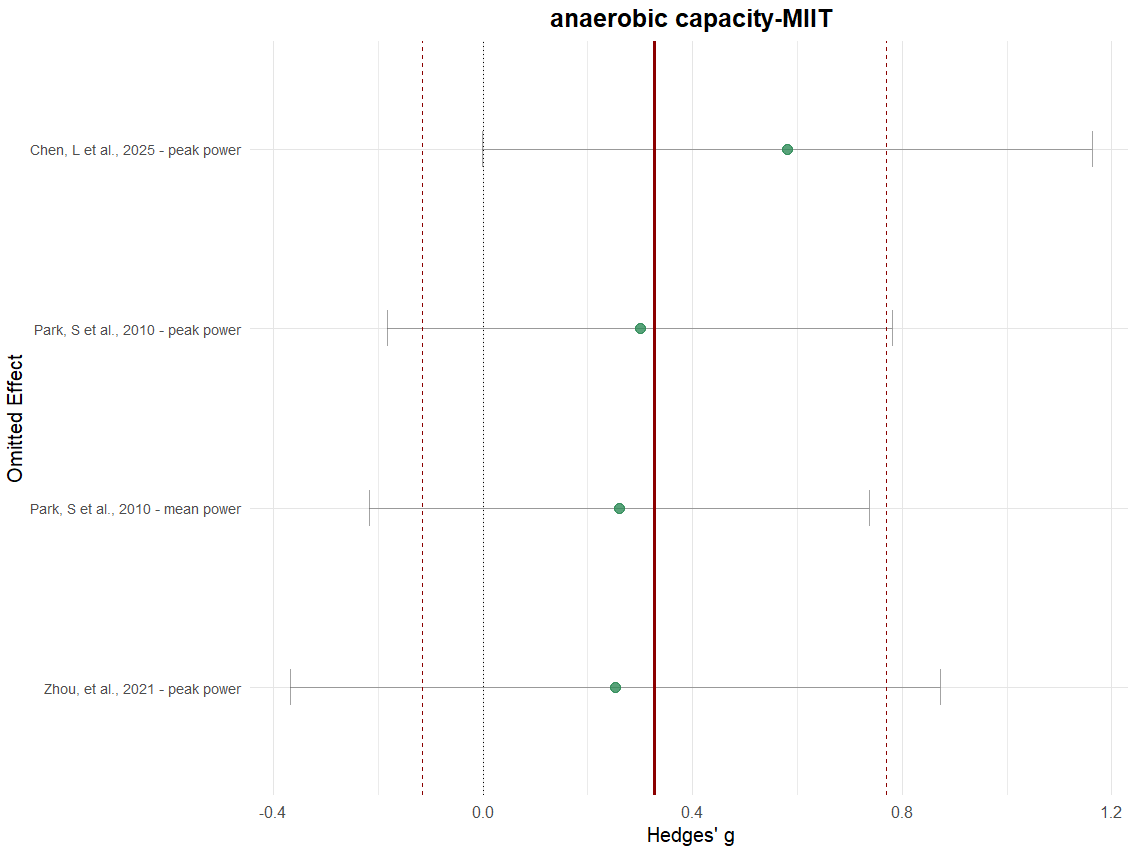 | 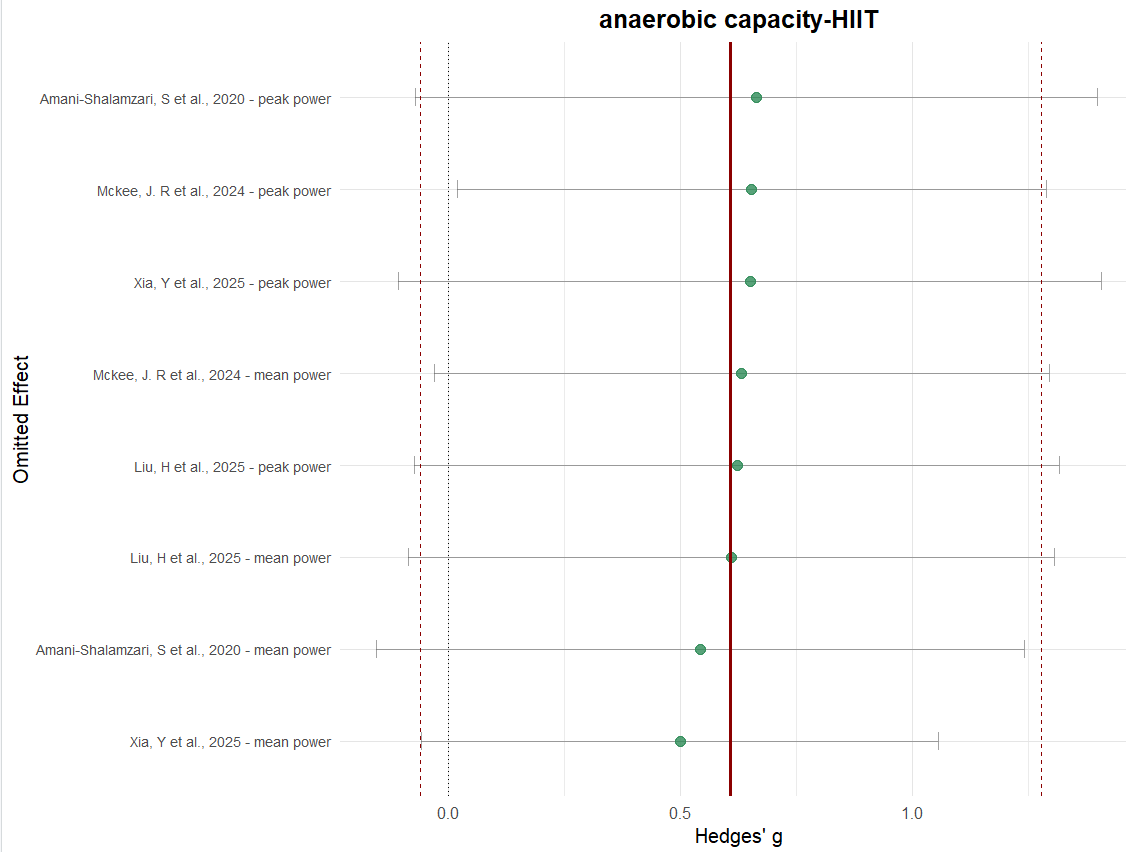 |  |
| 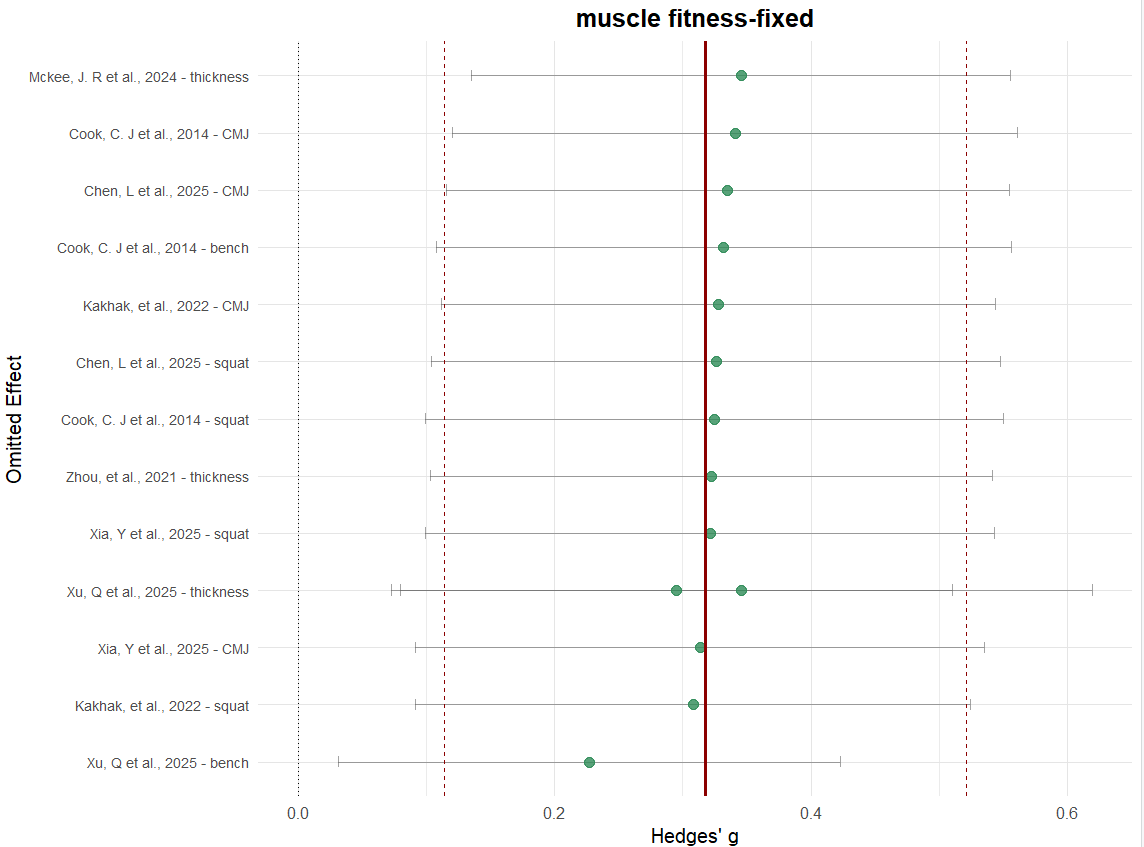 | 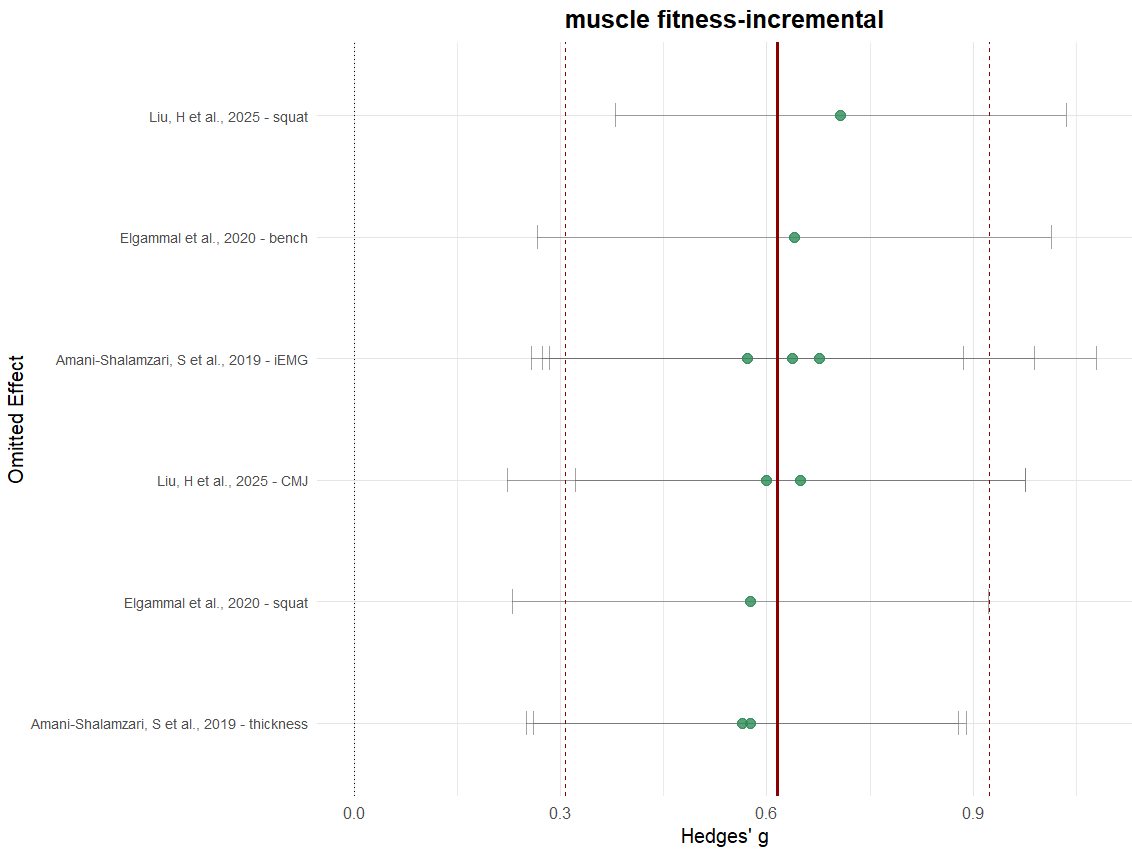 | 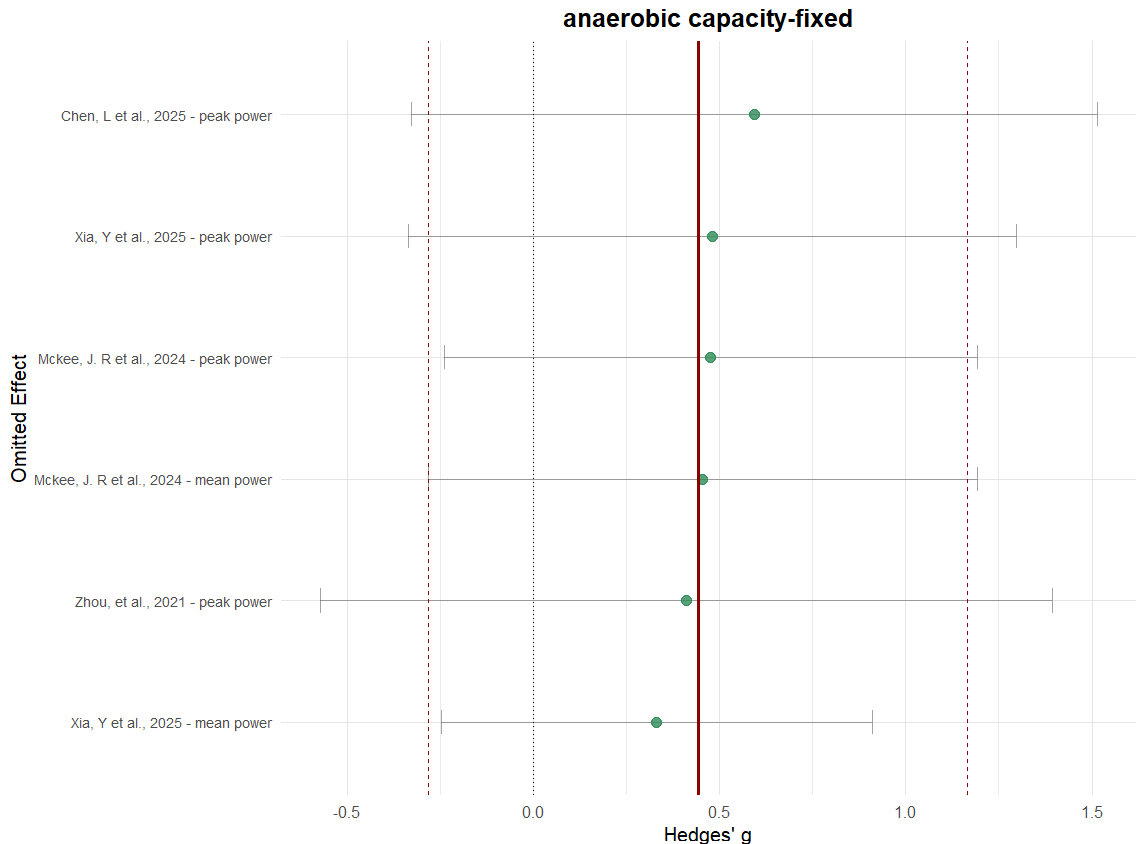 | 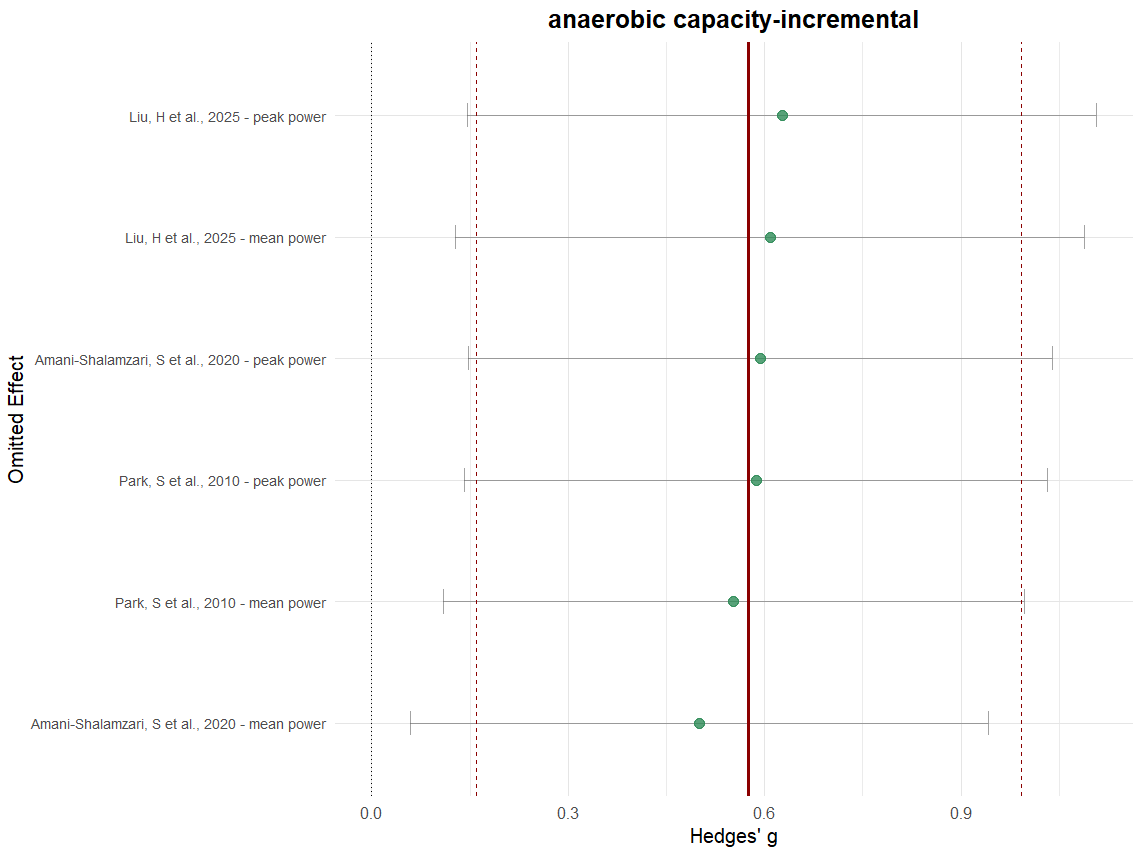 |  |
| 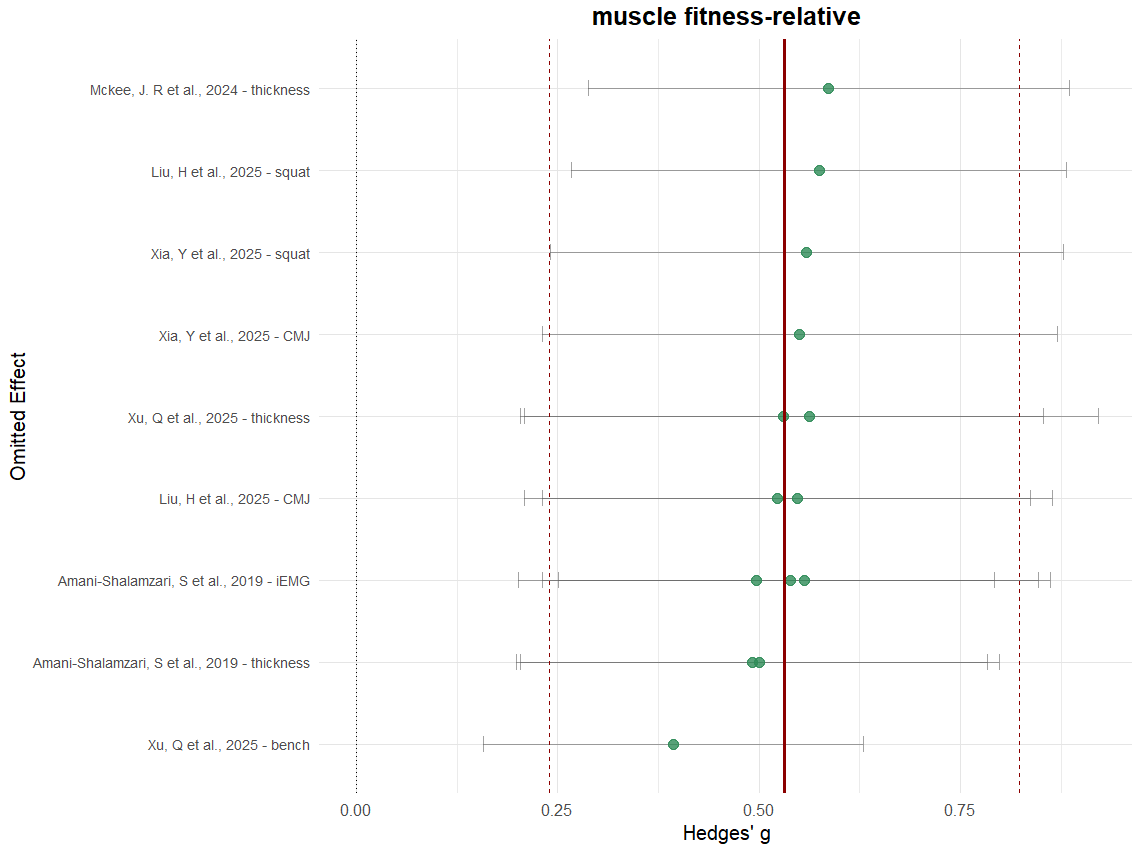 | 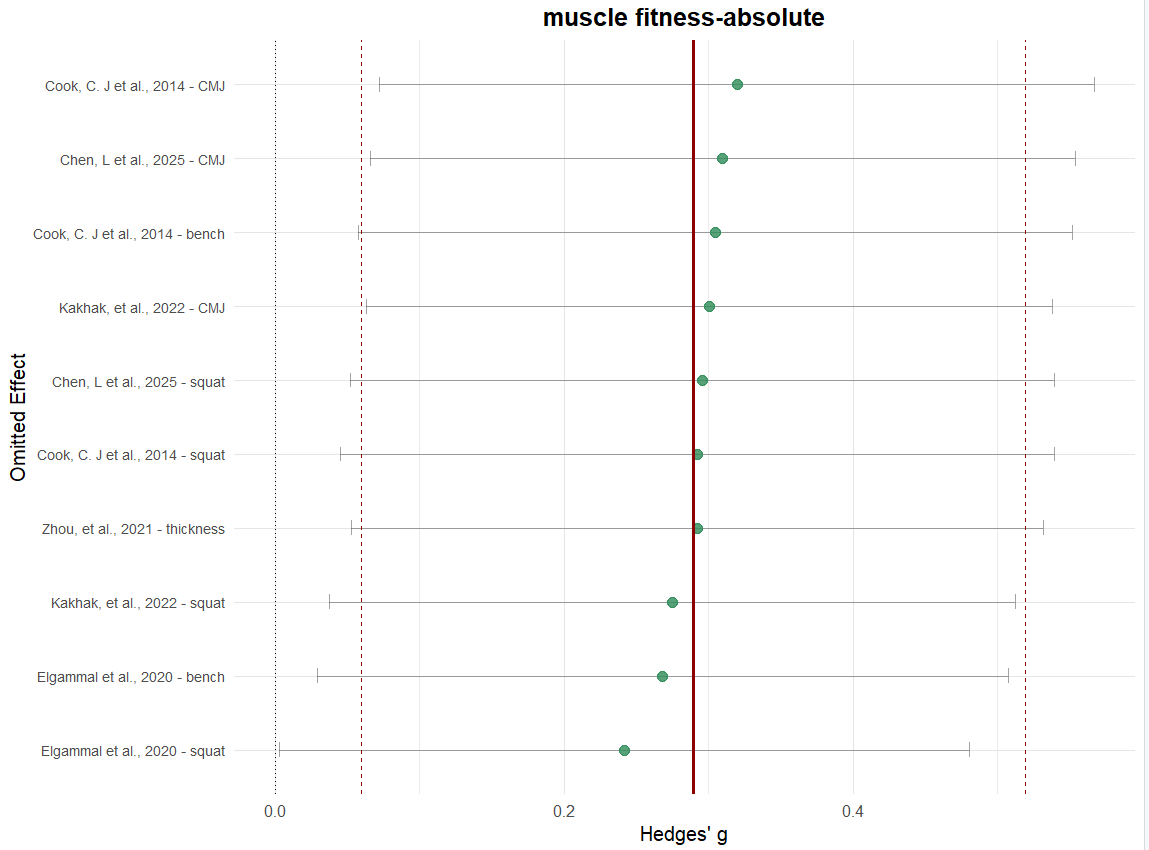 | 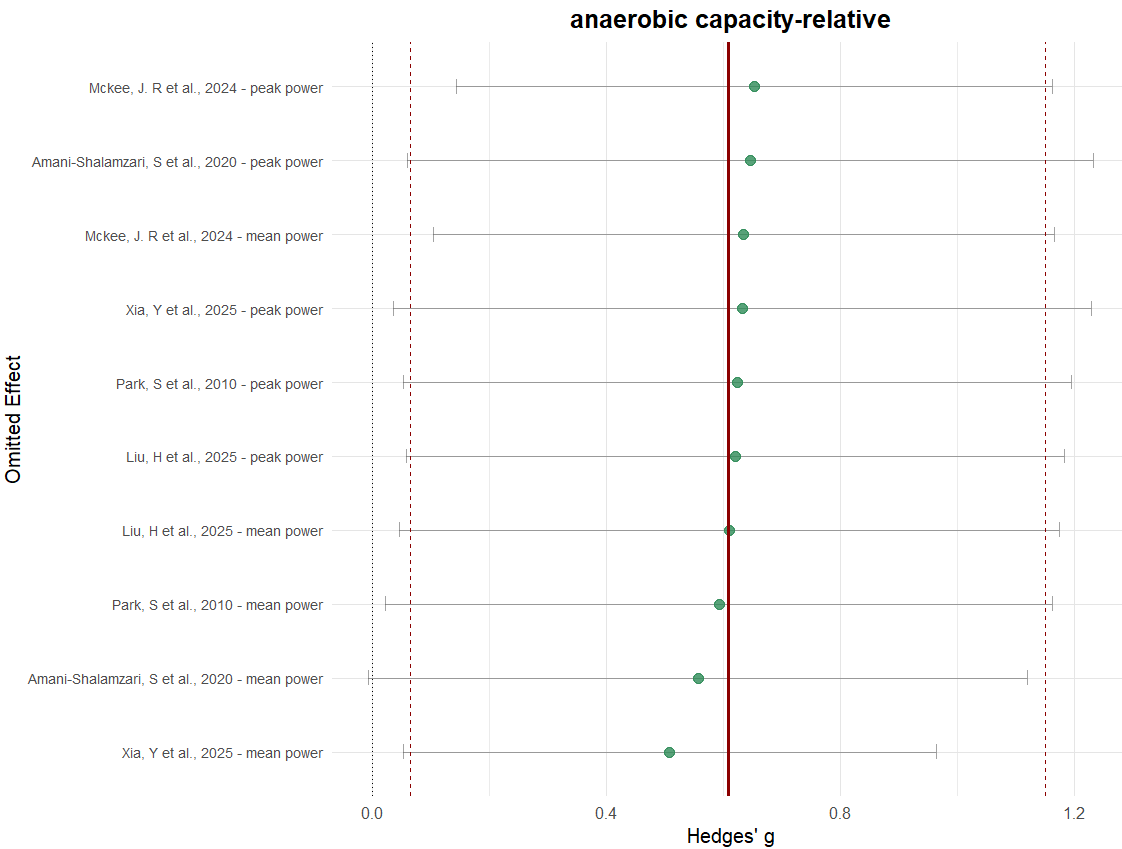 | 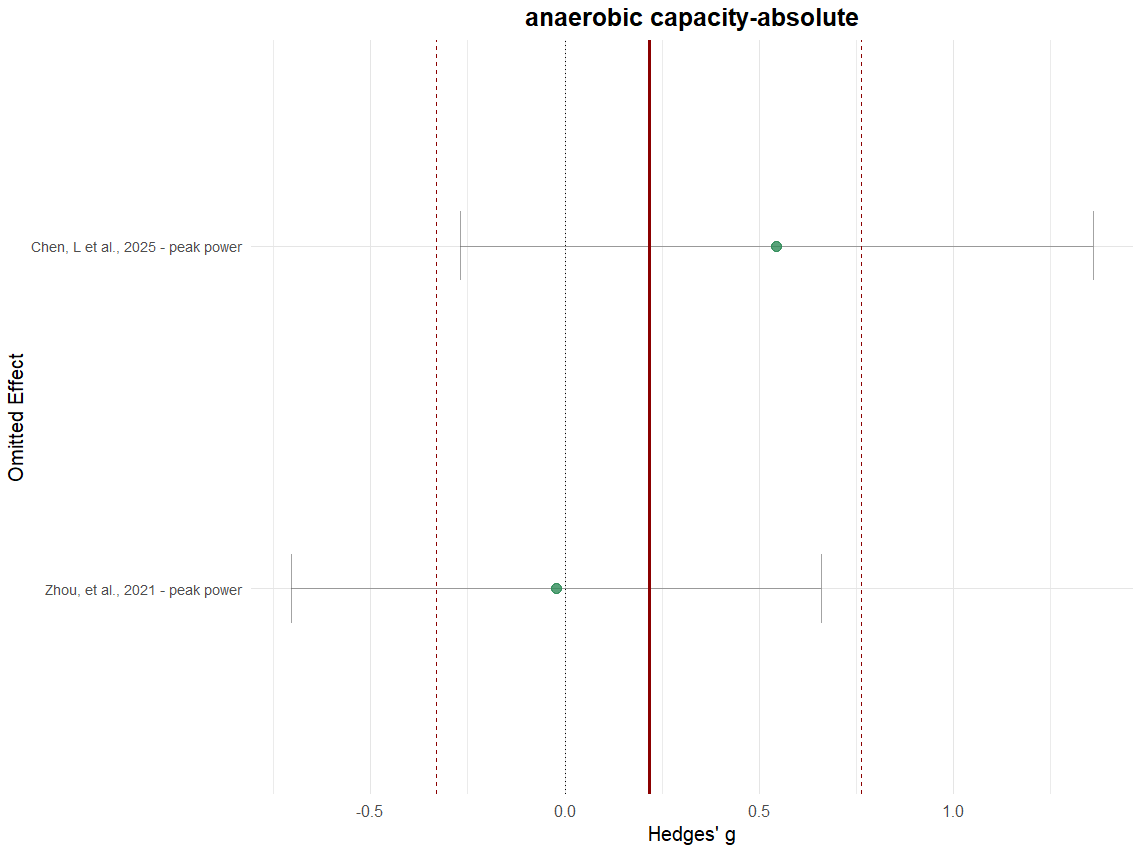 |  |

**Supplementary Material Appendix S6 (Three-level meta-analysis)**


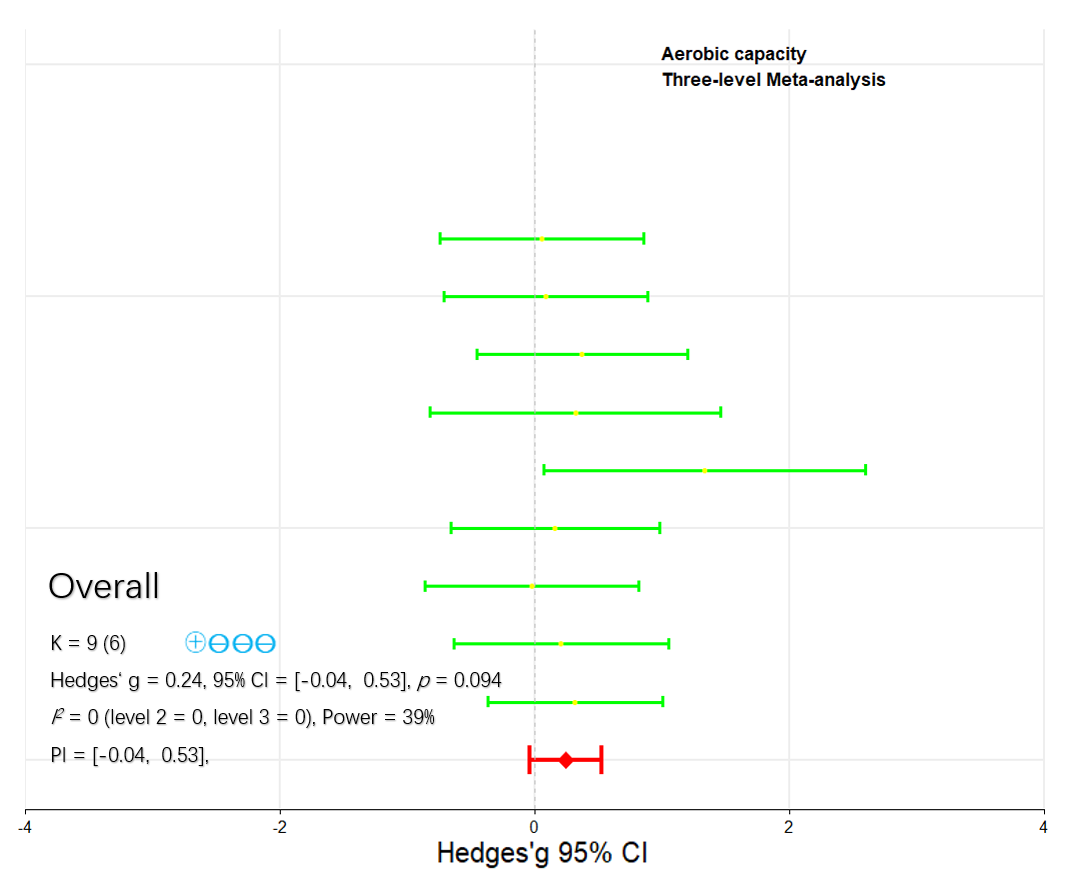

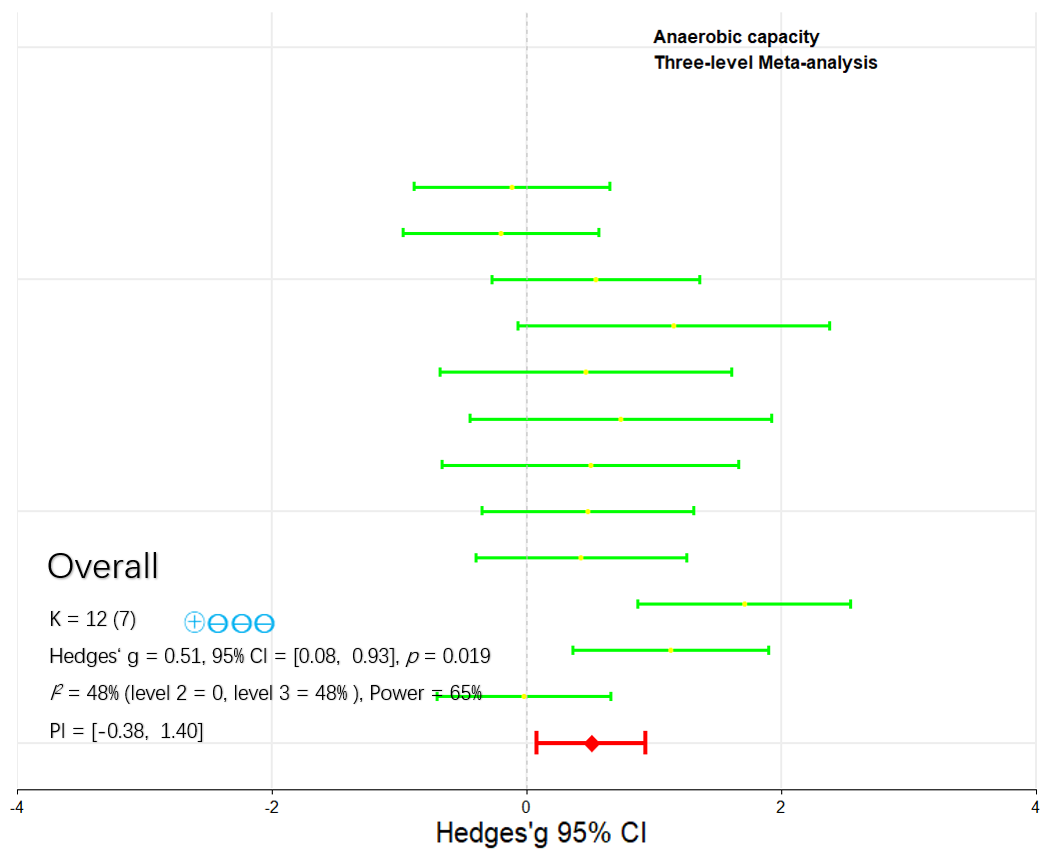


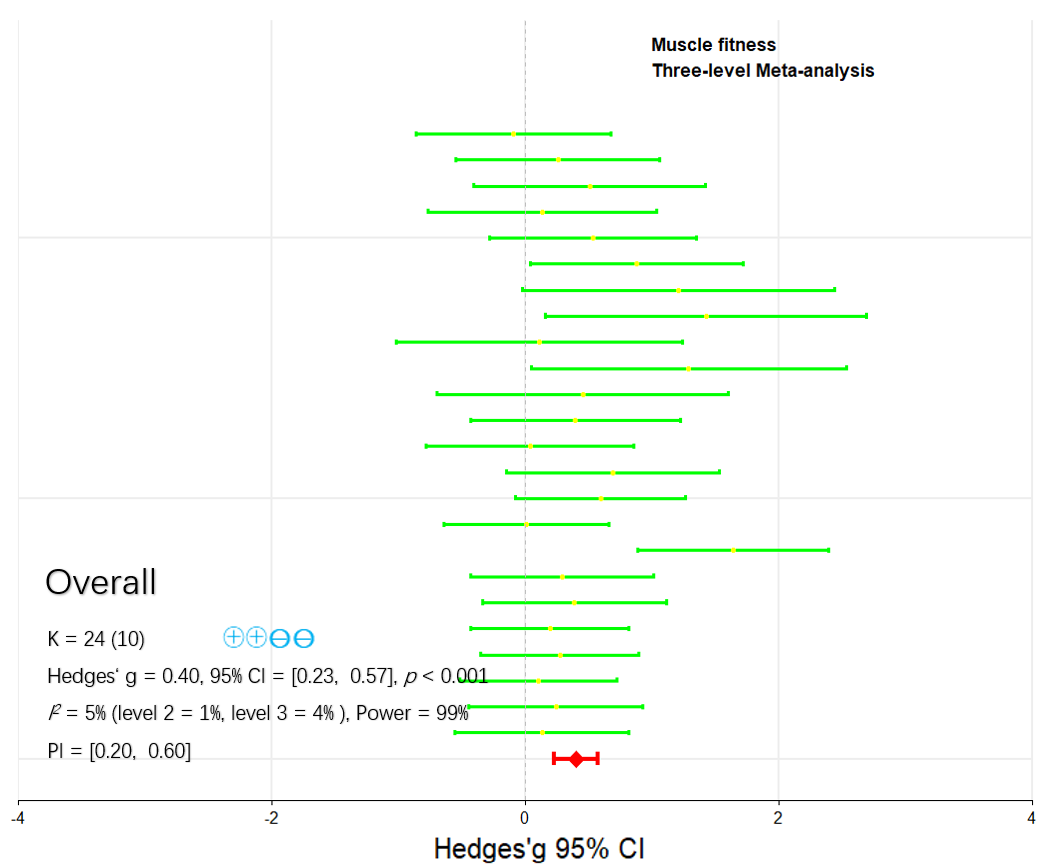

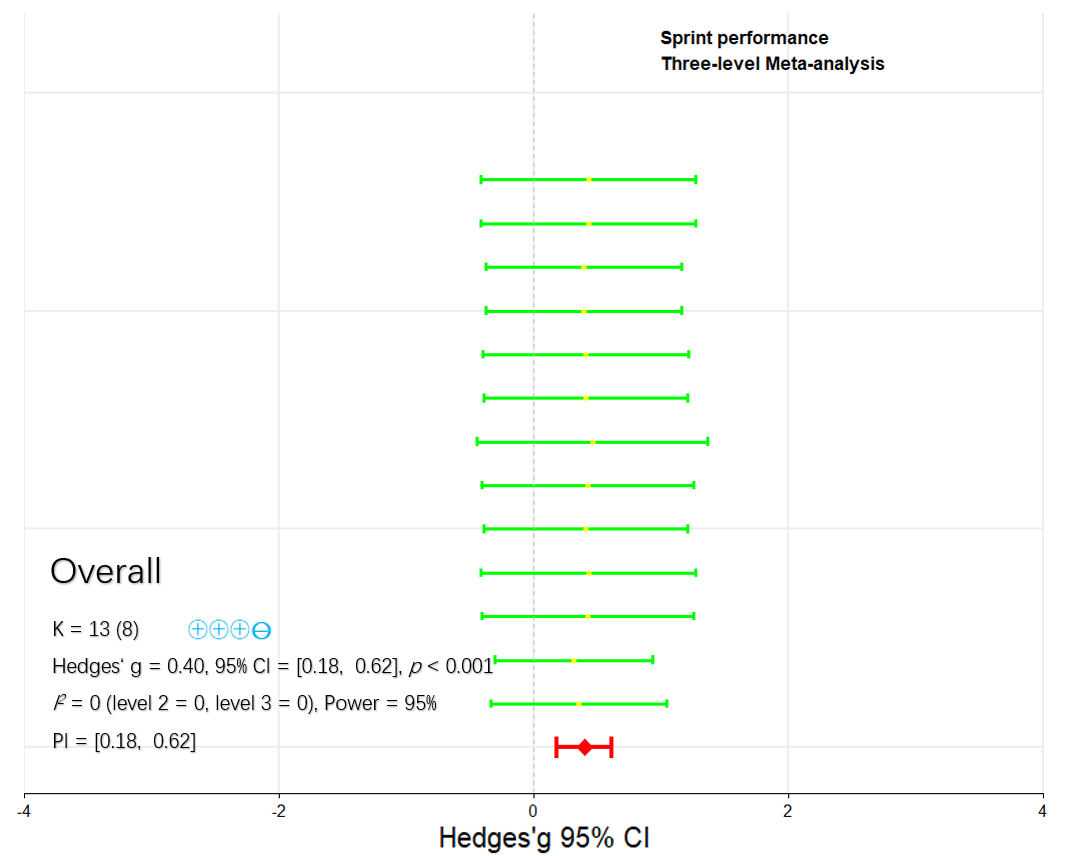


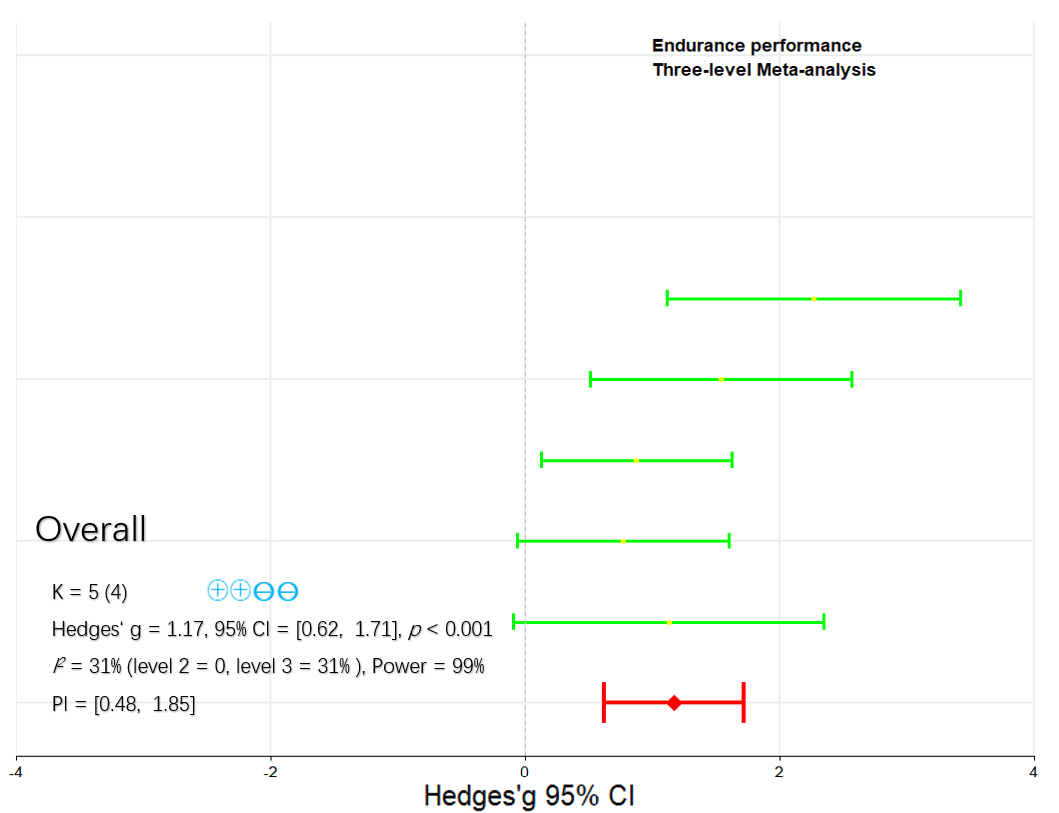


**Supplementary Material Appendix S7 (Funnel plot)**

| 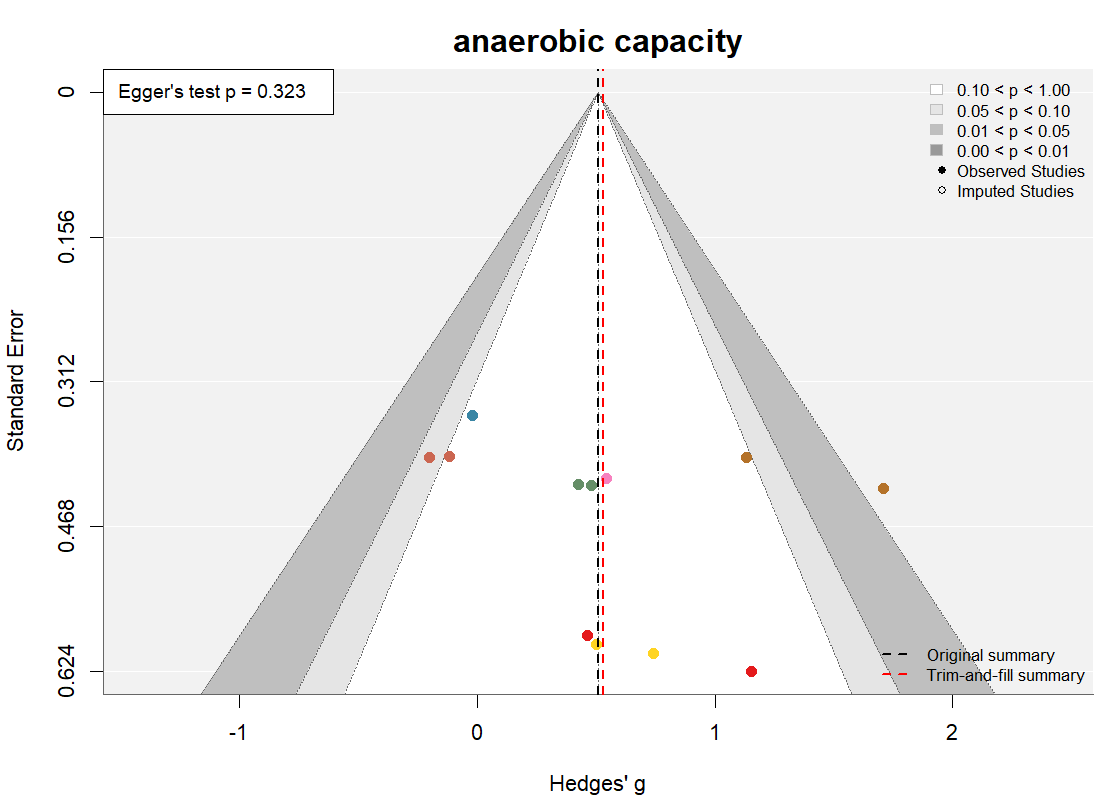 | 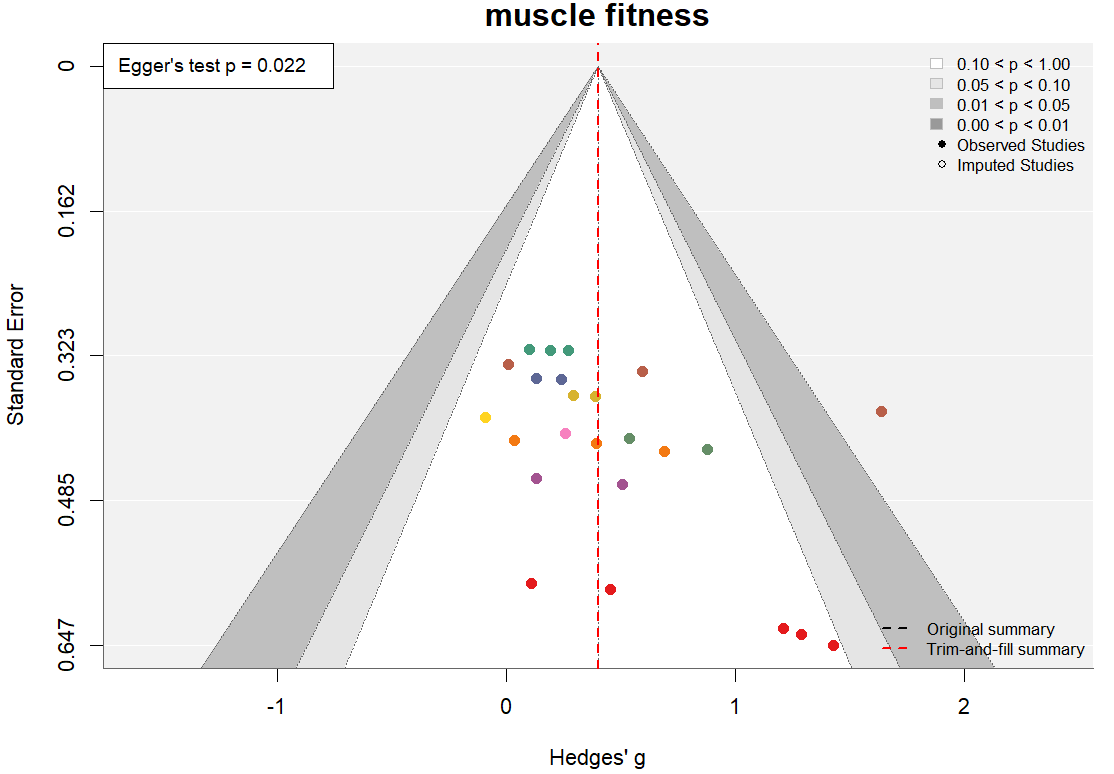 | 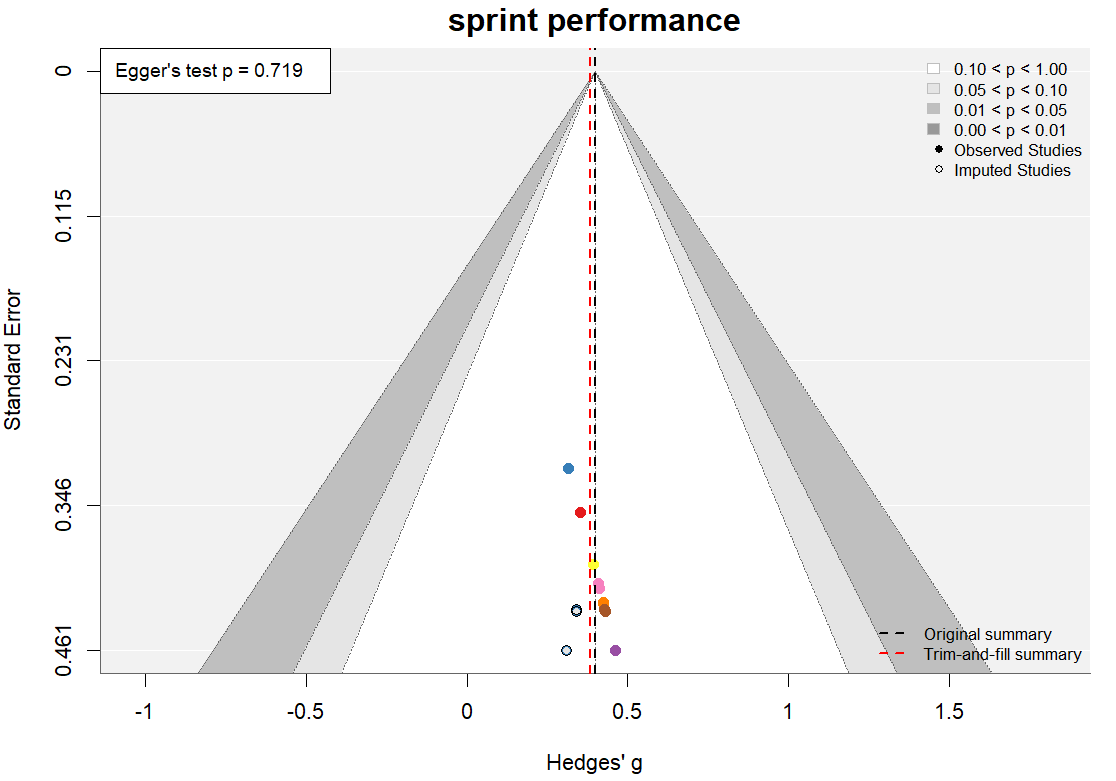 |
| --- | --- | --- |
| 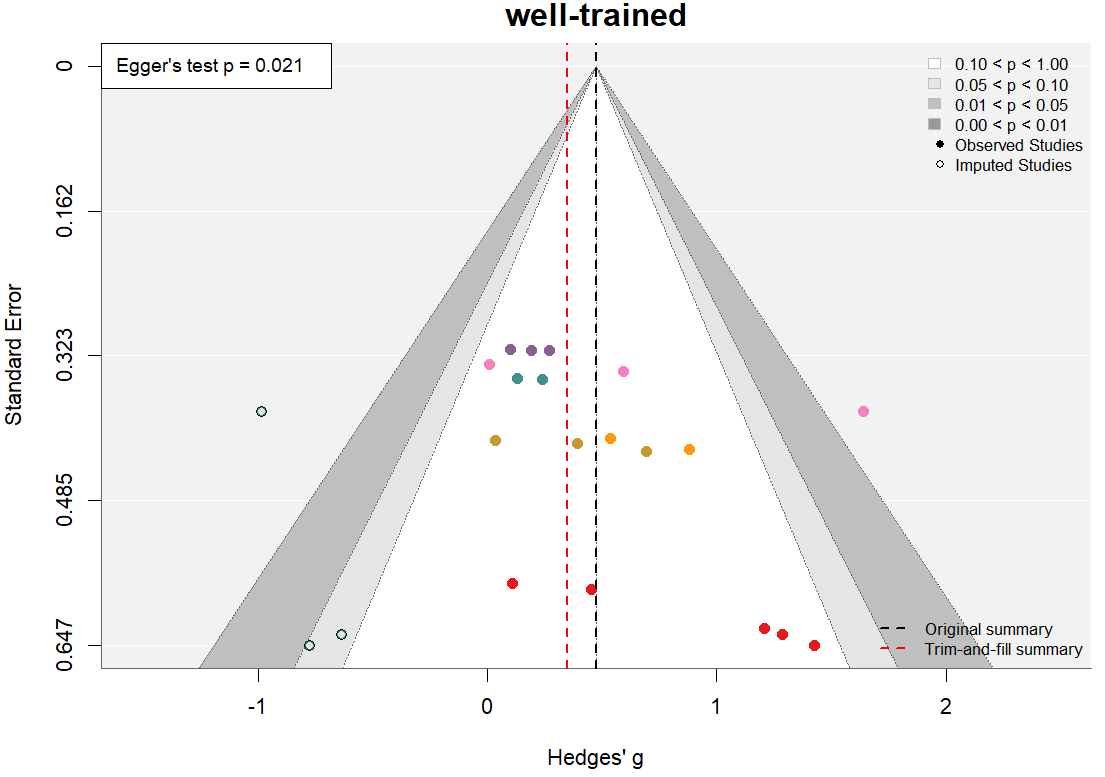 | 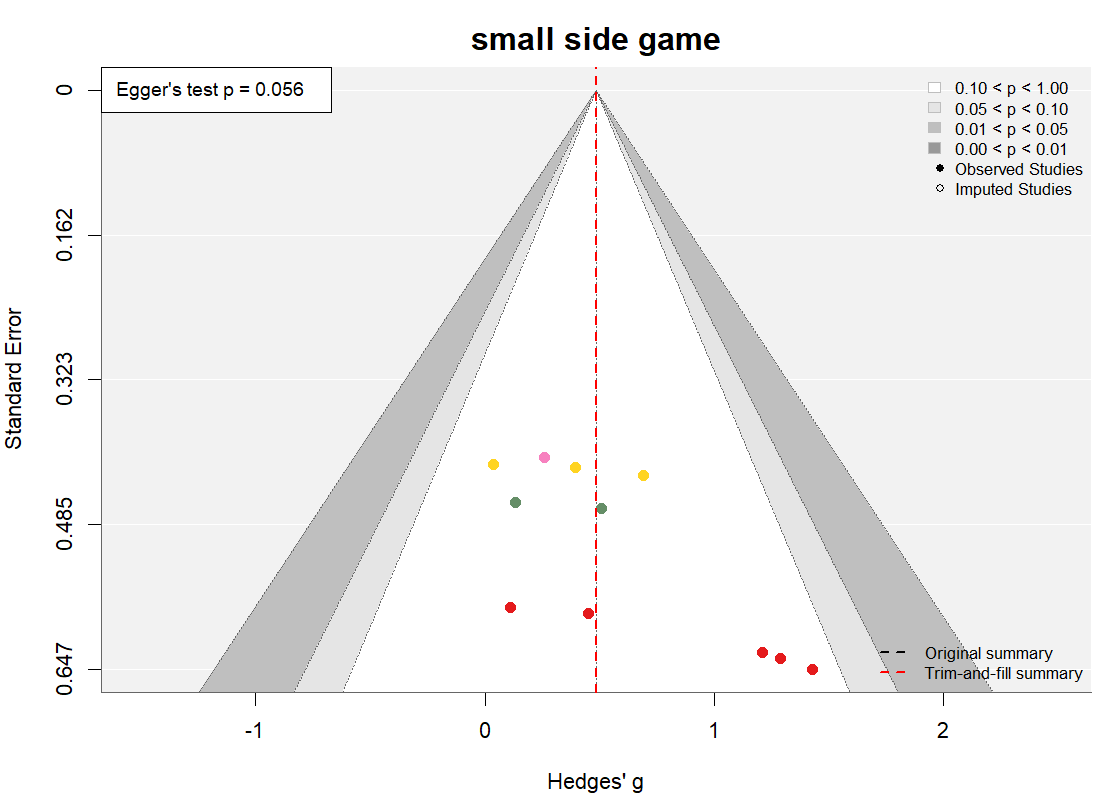 | 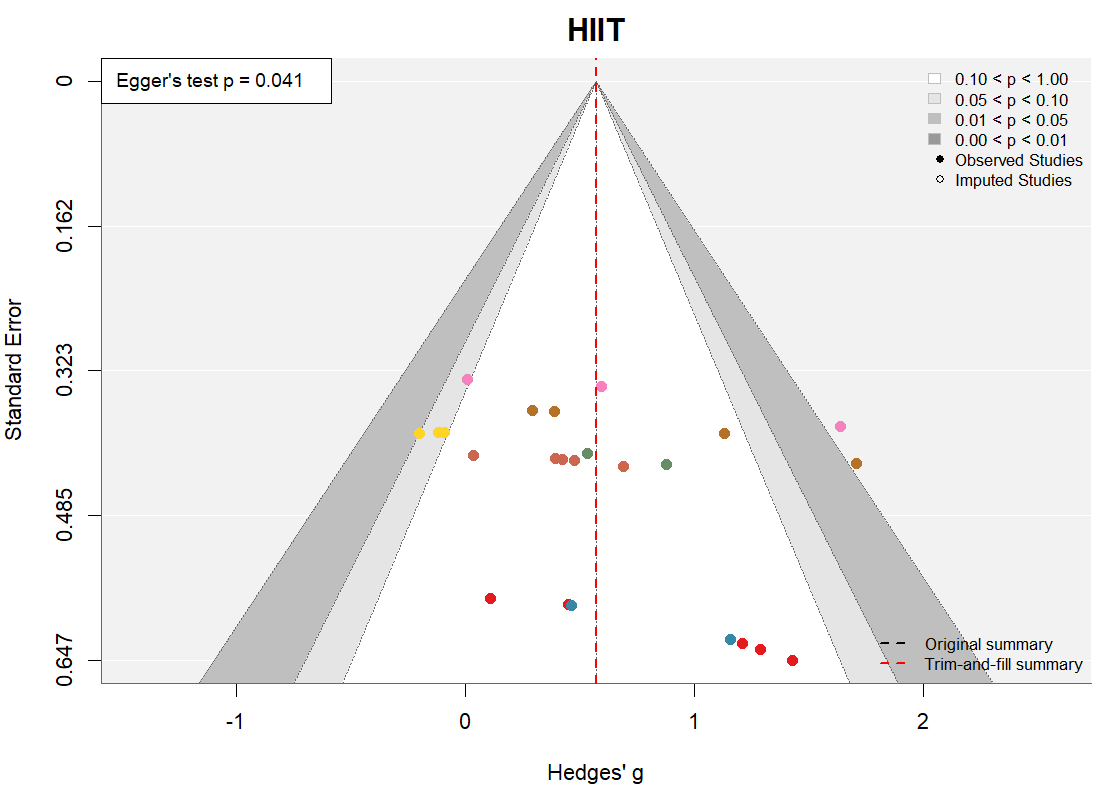 |
| 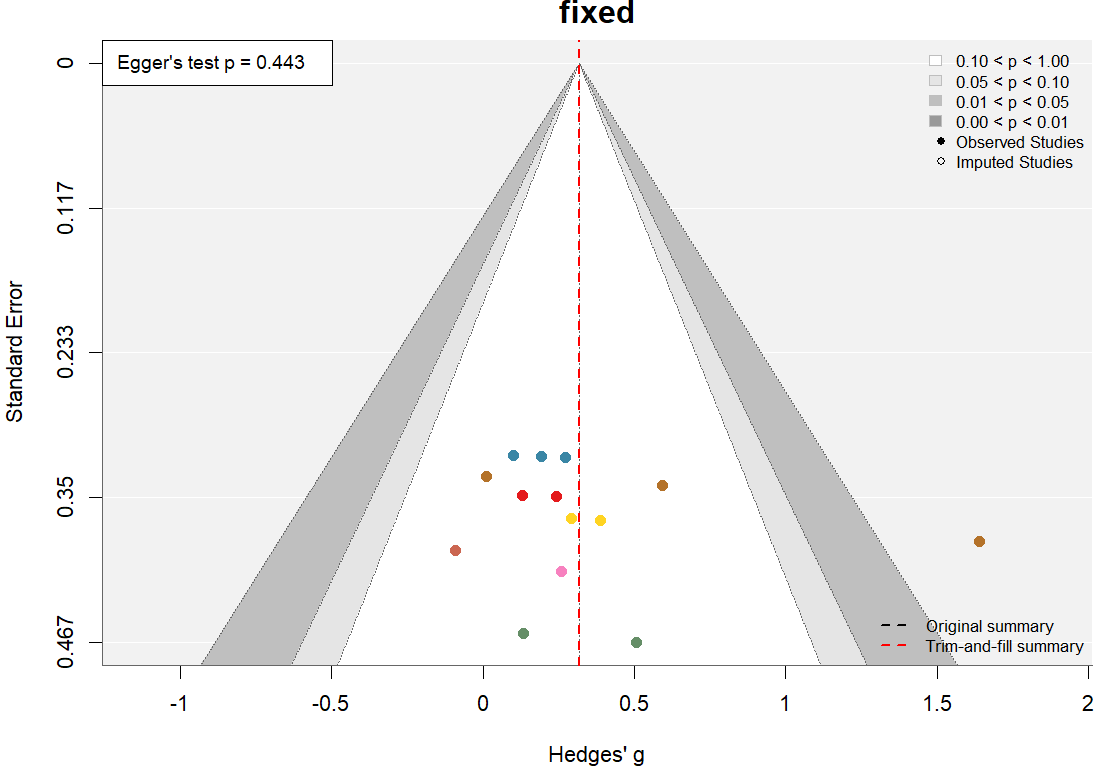 | 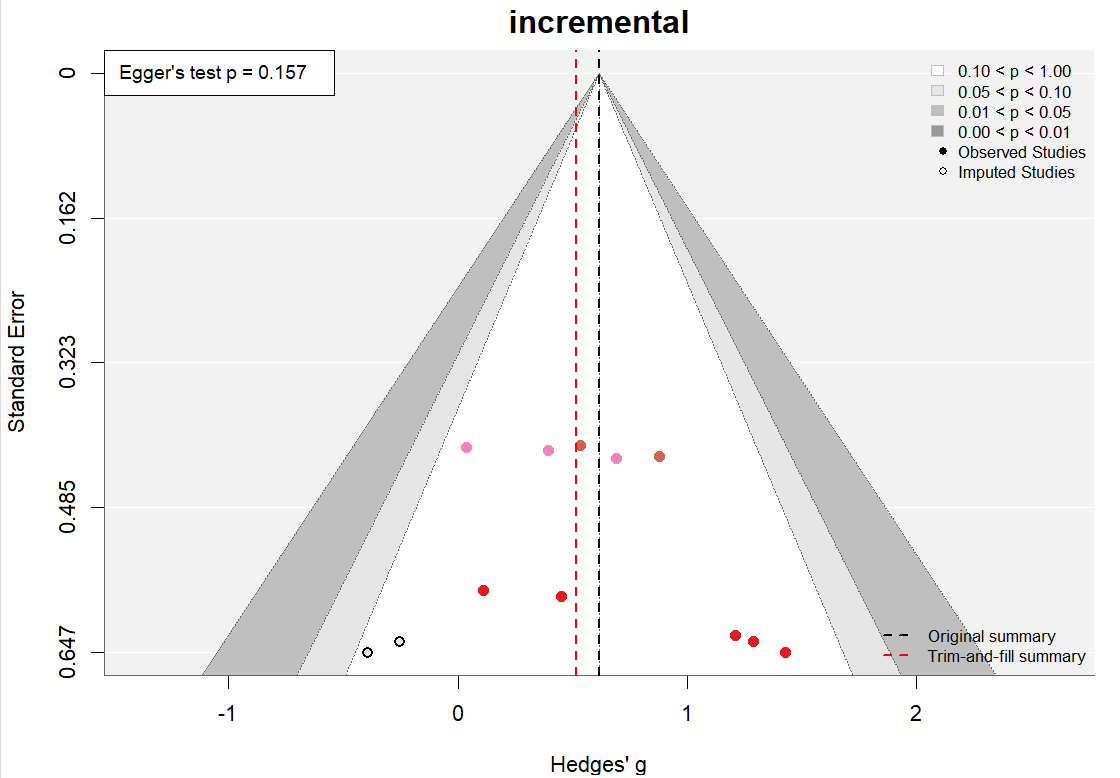 | 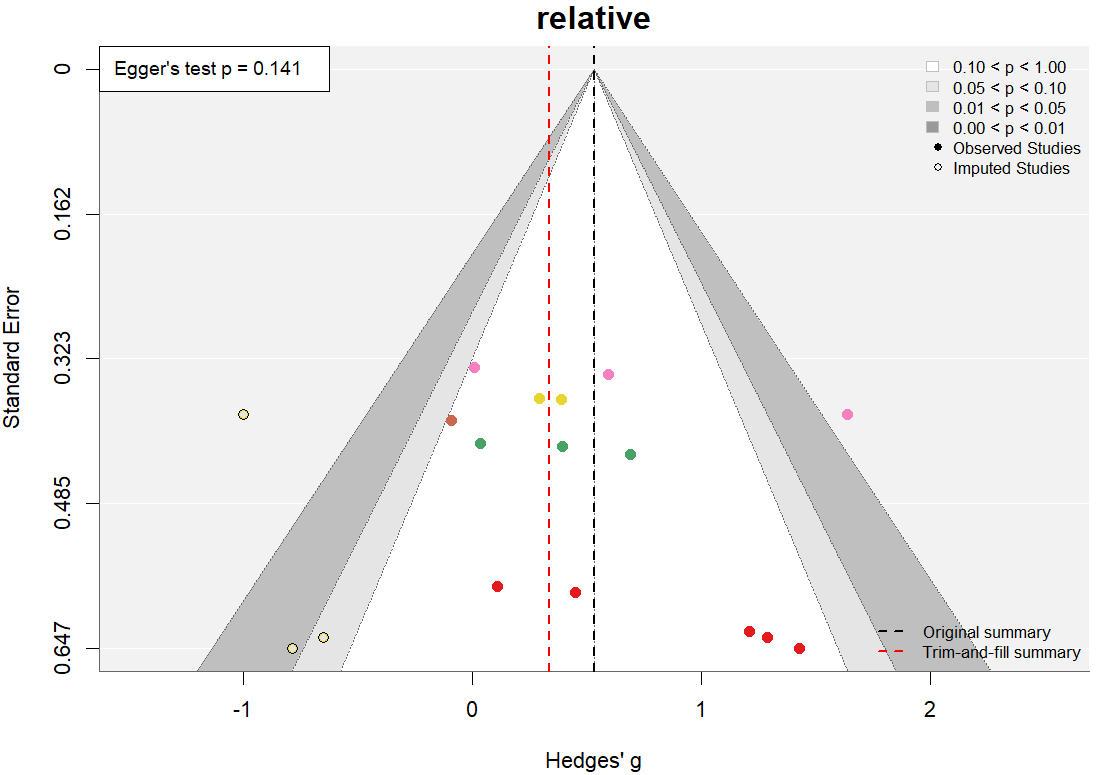 |
| 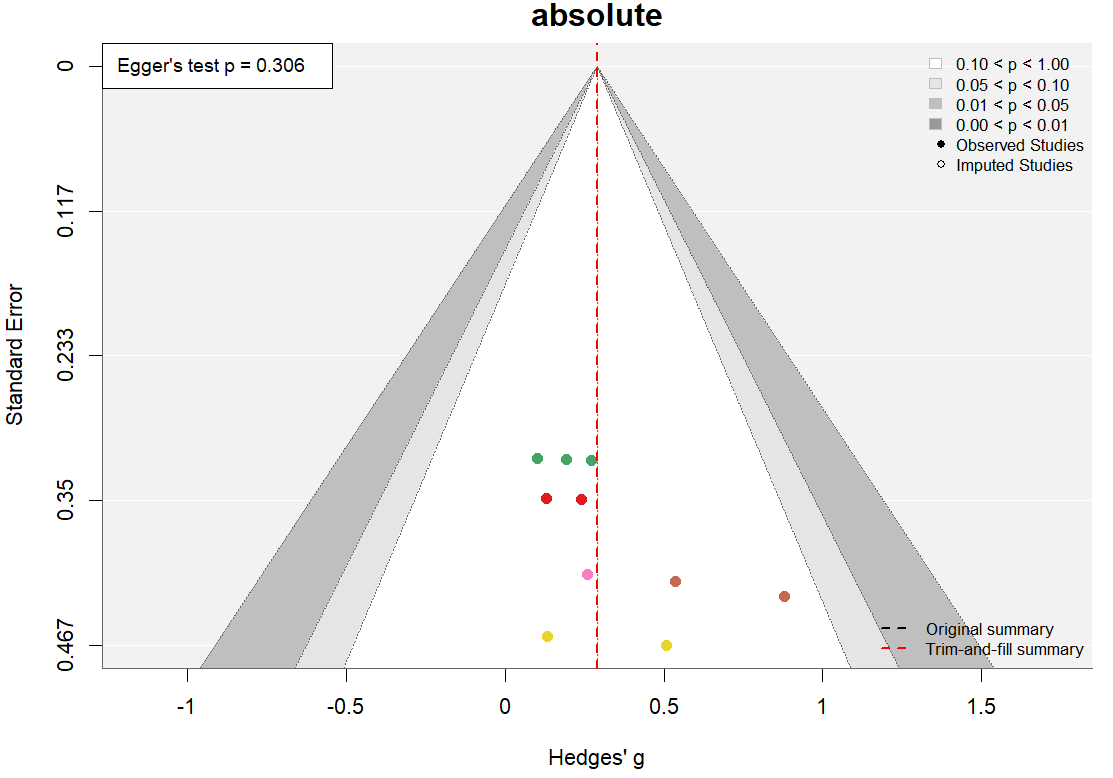 | 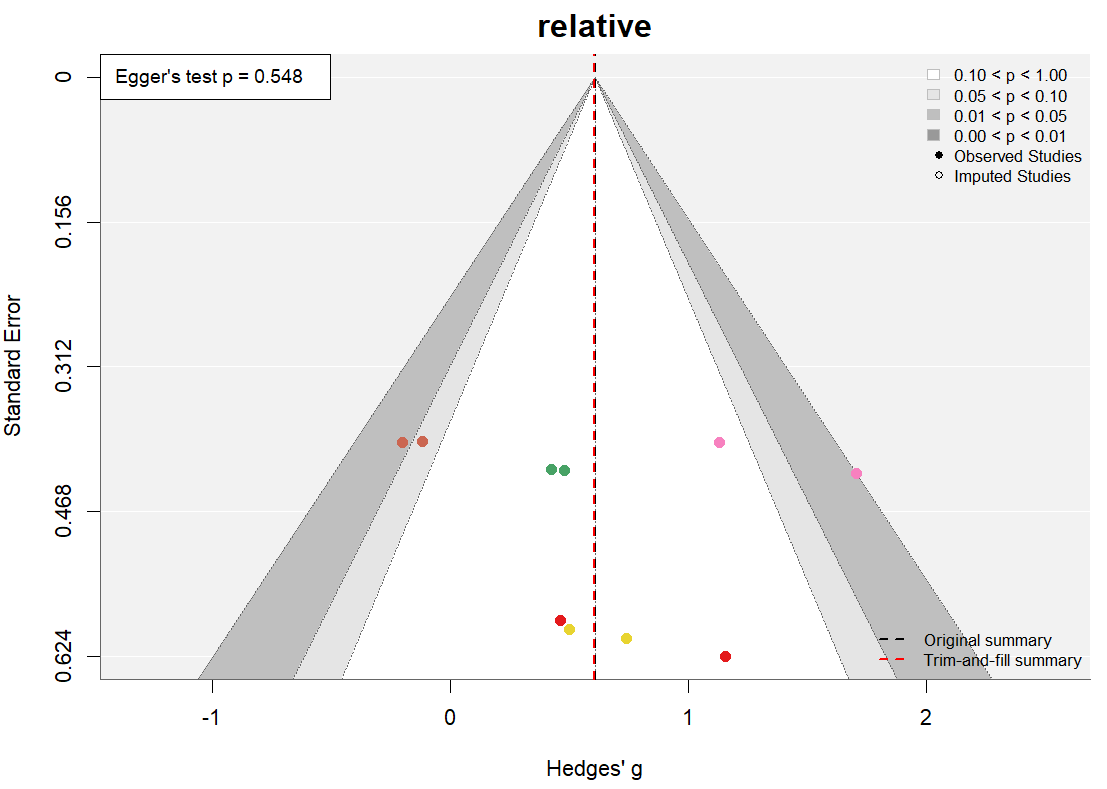 |  |
